# Drivers and implications of change in global ocean health over the past five years

**DOI:** 10.1371/journal.pone.0178267

**Published:** 2017-07-05

**Authors:** Benjamin S. Halpern, Melanie Frazier, Jamie Afflerbach, Casey O’Hara, Steven Katona, Julia S. Stewart Lowndes, Ning Jiang, Erich Pacheco, Courtney Scarborough, Johanna Polsenberg

**Affiliations:** 1 National Center for Ecological Analysis and Synthesis, University of California Santa Barbara, Santa Barbara, California, United States of America; 2 Bren School of Environmental Science & Management, University of California Santa Barbara, Santa Barbara, California, United States of America; 3 Imperial College London, Silwood Park Campus, Ascot, United Kingdom; 4 Conservation International, Arlington, Virginia, United States of America; Aristotle University of Thessaloniki, GREECE

## Abstract

Growing international and national focus on quantitatively measuring and improving ocean health has increased the need for comprehensive, scientific, and repeated indicators to track progress towards achieving policy and societal goals. The Ocean Health Index (OHI) is one of the few indicators available for this purpose. Here we present results from five years of annual global assessment for 220 countries and territories, evaluating potential drivers and consequences of changes and presenting lessons learned about the challenges of using composite indicators to measure sustainability goals. Globally scores have shown little change, as would be expected. However, individual countries have seen notable increases or declines due in particular to improvements in the harvest and management of wild-caught fisheries, the creation of marine protected areas (MPAs), and decreases in natural product harvest. Rapid loss of sea ice and the consequent reduction of coastal protection from that sea ice was also responsible for declines in overall ocean health in many Arctic and sub-Arctic countries. The OHI performed reasonably well at predicting near-term future scores for many of the ten goals measured, but data gaps and limitations hindered these predictions for many other goals. Ultimately, all indicators face the substantial challenge of informing policy for progress toward broad goals and objectives with insufficient monitoring and assessment data. If countries and the global community hope to achieve and maintain healthy oceans, we will need to dedicate significant resources to measuring what we are trying to manage.

## Introduction

With countries rapidly working to meet both Aichi Biodiversity Targets and newly-ratified UN Sustainable Development Goals (SDGs), governments, non-governmental organizations (NGOs), multilateral organizations, and the global community are eager to track countries’ progress towards achieving these ambitious environmental and social targets [1]. The need for adequate metrics to track this progress has led to the identification, consolidation, and, in some cases, development of indicators appropriate for this purpose. For example, both the Biodiversity Indicators Partnership, hosted by the United Nations Environment Program World Conservation Monitoring Centre (UNEP-WCMC) (unep-wcmc.org) and the Biodiversity Dashboard, hosted by NatureServe (dashboard.natureserve.org), provide platforms for vetting and sharing indicators for the 20 different Aichi Biodiversity targets. Further, the United Nations established an Inter-Agency Expert Group to identify and nominate indicators for use in tracking progress towards the 17 SDGs [2]. Additionally, many countries around the world have set their own policy goals for sustainable development and are developing or tracking indicators for those purposes (e.g., [3–6])

For indicators to be effective at tracking progress towards achieving these policy objectives they need to be assessed regularly and updated with the best available data and science [7]. These criteria, however, create significant challenges. Repeatability of measurements and assessments requires sufficient and committed resources to maintain data collection, management, processing, archiving, and distribution. In addition, while using the best available data and science in repeated assessments helps ensure indicators are as accurate and up-to-date as possible, incorporating or adapting to new and/or different types of data and science can lead to changes in the underlying indicator construct, making comparisons over time more challenging. Global indicators (such as for the Aichi Biodiversity targets and SDGs) must also be sufficiently inclusive of most, or ideally all, countries, which creates additional hurdles stemming from inevitable gaps in data [8].

The Ocean Health Index (OHI) [9], updated annually since 2012, is one of the indicators identified for use in tracking Aichi Target 10 and SDG 14. It is also being used widely at regional and local scales to assess ocean health and inform regional policy and decision making [10]; ohi-science.org/projects). Based on the premise that a healthy ocean sustainably delivers a range of benefits to people now and in the future, OHI measures how well countries are performing in achieving maximum sustainable flows of 10 key ocean benefits, called goals (Table 1). Success is expressed on a scale of 0 to 100 toward the achievement of a designated target, or reference point. Global assessments score individual goals in each coastal region (country or territory), and goal scores are averaged into an Index score for each region, which are then used to calculate global Index and goal scores (S2 File).

**Table 1:** Updates to status and trend data and models.

Goal/subgoal	Updates to data	Updates to data prep or model	Notes
Artisanal opportunities	*Need*: Additional years of data, and a slightly different version of the WorldBank dataset to control for inflation *Opportunity*: None	Reference point for “need” data is now the 95^th^ quantile among regions (rather than max value)	The change in reference point increased scores
Biodiversity: Species	*Assessed species*: Additional years of IUCN (species status and range) and Aquamaps (species range) data	Added bird species from BirdLife International Used a new threshold for determining presence/absence of species in Aquamaps data. In addition to informing our analysis this information will be useful to other researchers. The conclusions have been written up in a paper submitted to PLoS (O’Hara et al.)	The addition of bird data generally increased scores because there are several bird species that are widespread and at low extinction risk
Biodiversity: Habitats	*Sea ice edge*: Additional years of data (trend and condition updated) *Mangrove*: None *Saltmarsh*: None *Seagrass*: None *Coral*: None *Softbottom*: New SAUP fisheries data (trend and condition updated)	None	The National Snow & Ice Data Center updated their data, but this did not affect our scores in any significant way.
Food Provision: Fisheries	*Catch*: Improved Sea Around Us data (now provided as rasters at a 0.5 degree grid level and in categories such as commercial, subsistence, etc.); unfortunately, no additional years of data (2010 most recent year). *B/B*_*MSY*_: Now RAM B/B_MSY_ data used when possible (these data are based on formal stock assessments); updated values with new catch data using the data-limited catch-MSY method	Simplified method of calculating B/B_MSY_. Previously, model priors depended on the region’s fisheries resilience score (based on data from Mora et al. 2009). However, our analyses suggest this does not improve results. Modified the taxonomic penalty for catch not identified to species because these penalties were magnified due to the use of the geometric mean to estimate average stock condition. We explored several models (including ensemble approaches) besides the catch-MSY to generate B/B_MSY_ values, but preliminary analyses did not indicate these methods were better than the catch-MSY approach	2016 scores were very different than 2015 scores. This was due primarily to differences in the SAU catch data. One of the main differences is that, in some places, more catch is now identified as “marine fishes not identified”. When catch is not identified to the species level, it is penalized in the fisheries model because this is considered an indicator of poor management. This tended to decrease scores. Other variables affecting scores: Changes to the taxonomic penalty in the model (increased scores) Addition of RAM data for B/B_MSY_ scores Changes to catch-MSY calculations Better resolution data
Food Provision: Mariculture	*Production*: Additional years of FAO data *Sustainability*: None *Population*: Updated methods (estimates calculated using higher resolution spatial data)	None	Retroactive changes to FAO data resulted in some differences in scores
Coastal protection	*Sea ice shoreline*: Additional years of data (trend and condition updated) *Mangrove*: None *Saltmarsh*: None *Seagrass*: None *Coral*: None	None	The National Snow & Ice Data Center updated their data, but this did not affect our scores in any significant way.
Carbon storage	*Mangrove*: None *Saltmarsh*: None *Seagrass*: None	None	None
Clean waters	*Nutrient pollution*: Additional years of FAO fertilizer data (trend and pressure) *Chemical pollution*: Shipping and ports: None Land-based inorganic: None Land-based organic: Additional years of FAO pesticide data *Pathogens*: None *Trash*: None for pressure data, but trend data was updated with a new dataset	Previously we used population as a proxy for the trash trend. Now we use trends in plastic disposal.	Replacing the trash trend data had a very small effect on scores. On average clean water scores decreased slightly, but less than 5 points.
Sense of Place: Iconic species	*Assessed species*: Additional years of (species status and range) and Aquamaps (species range) data. Added additional region specific iconic species to master list.	Trend is now calculated using historical changes in IUCN risk category. This is a huge improvement over the previous method which relied on the IUCN population trend data.	Scores generally increased because the previous method overestimated trend effects. More accurate scores in Baltic regions due to changes in iconic species list.
Sense of Place: Lasting special places	*Area*: Additional years of WDPA data *Spatial data*: Improved estimates of offshore/inland areas	None	Retroactive changes to source data and changes to area altered scores slightly in most regions, although changes to WDPA data had a large effect on a handful of regions
Natural products	*Harvest*: Two additional years of FAO data	Corrected how fishery scores are integrated into score calculations (used as sustainability component of fish oil product)	Previously, the 2015 assessment used 2011 data, now it uses 2012 data Retroactive changes to source data changed scores somewhat Countries that have sporadic harvests have high yearly variation in scores Changes to fishery scores alter scores
Tourism and recreation	*Tourism sustainability*: None *Employment*: New year of WEF data *Travel warnings*: New year of data	Improved approach to dealing with travel warnings in the model	Retroactive changes to source data changed scores and there were a few changes to the travel warning classifications
Livelihoods & economies	*Jobs*: None *Wages*: None *GDP*: None	None	None

Description of updates to data and models used to calculate the status and trend scores for the global Ocean Health Index 2016 assessment.

After the second year of assessment [11], we found that global goal scores changed by -1.2 to +6.5 points while the entire global Index score increased by one point. Scores for individual regions (and goal scores within regions) often changed much more. Because these results were based on just two years of assessments, they offered only preliminary indications of how ocean health was changing. Now, with three additional years of assessment, improved methods and data, increased data availability, and a range of notable governance changes (e.g., dramatic increase in marine protected area (MPA) designations around the world), we are better positioned to assess how and why ocean health has changed recently, both globally and for every coastal region around the world. This fifth year of global assessment also offers an opportunity to evaluate how well the OHI framework tracks changes in ocean health, in particular with respect to sustainability, in quantifiable and repeatable ways.

With a fifth consecutive year of global ocean health assessment completed, we address three key questions in this paper. First, how have global and region-level (countries or territories) OHI scores changed in the last five years, and what are the possible causes and consequences of those changes? Second, what are the challenges and opportunities for composite indicators to incorporate the best available data and science each year? Finally, what have we learned by repeating and improving the OHI assessment each year?

## Methods

### Calculating OHI scores

The philosophy and structure of the OHI as well the methods for processing data and calculating global scores are provided in the supporting information (S2 File), as well as in previous publications [9,11] and on our project website (ohi-science.org/ohi-global). In brief, OHI measures the current status of the sustainable delivery of each goal relative to a target (i.e., reference point) and the likely future status, which is indicated by combining information on the current status, recent trends, existing pressures to the status, and ecological and governance measures in place that build resilience [9].

Many updates have been made since 2013, the year of the last published assessment [11], incorporating data and science that subsequently became available. These changes are summarized in Tables 1 and 2 and described below. All goals were updated with improved data and/or methods, except coastal livelihoods & economies due to termination of key data sets (addressed below in the Discussion). All analyses were done using R [12].

**Table 2:** Updates to pressure data and models.

Pressure	Updates to data	Updates to data prep or model	Notes
Social: World Governance Index	Additional years of data	Small improvement to gapfilling	Small changes in a handful of countries
Social: Social Progress Index	New pressure layer	New pressure layer	Tended to increase pressure scores because SPI scores tend to be higher than WGI scores (the other component of social resilience)
Climate change: Ocean acidification	Additional years of data	Improved rescaling method: values greater than biological threshold of 1 are rescaled based on their absolute change in aragonite saturation state weighted by distance to 1 (closer to 1, higher pressure value)	Pressure scores tended to decrease very slightly
Climate change: UV	Additional years of data; limited data to only one dataset (previous calculations used a different dataset for the reference point)	Improved reference point; New reference point is the 99.99^th^ quantile of the entire time series	Very slight decrease in pressure score
Climate change: Sea level rise	Improved data (higher temporal resolution) along with more recent data	Clip pressure to near offshore areas (rather than including entire EEZ, which is not biologically relevant); Improved reference point: 99.99^th^ quantile across the entire timeseries	Very small (<5 points) increase in pressure score
Climate change: Sea surface temperature	None	Improved reference point: 99.99^th^ quantile across the entire timeseries	Resulted in a slight increase in pressure scores (most regions < 2.5 points)
Pollution: Land-based nutrient pollution	Additional years of FAO fertilizer data	None	
Pollution: Chemical pollution	Organic land-based: additional years of FAO pesticide data Shipping ports: None Inorganic land-based: None	None	
Pollution: Trash	None	None	
Pollution: Pathogens	None	None	
Species: Genetic escapes	Additional years of data	None	
Species: Targeted harvest	Additional years of data	None	Retroactive changes to source data resulted in small changes to pressure score
Species: Invasive species	None	None	
Commercial fisheries: high bycatch	Improved Sea Around US data (now provided at raster spatial scale and by sector); Net primary productivity used to standardize catch was updated	Small change to reference point (99.99^th^ quantile across the entire time series) Artisanal catch removed (not possible previously because catch data was not categorized into types)	Relatively small (<10 points) increase in pressure score
Commercial fisheries: low bycatch	Improved Sea Around US data (now provided at raster spatial scale and by sector); Net primary productivity used to standardize catch was updated	Small change to reference point (99.99^th^ quantile across the entire time series) Artisanal catch removed (not possible previously because catch data was not categorized into types)	Relatively small (<10 points) increase in pressure score
Artisanal fisheries: low bycatch	Improved Sea Around US data (now provided at raster spatial scale); Net primary productivity used to standardize catch was updated	Catch includes: artisanal, subsistence, and recreational catch (SAUP catch data now categorized)	Relatively small (<5 points) increase in pressure score
Artisanal fisheries: high bycatch	None	Values now include blast and poison data (previously only included blast data) Values are now averaged over 3nm offshore area rather than the entire EEZ, which aligns better with where artisanal fishing occurs	Tended to increase pressures in a few regions
Habitat destruction: soft-bottom subtidal	Improved Sea Around Us data (now provided at raster spatial scale)	None	No large changes in scores
Habitat destruction: Intertidal (nearshore population used as proxy)	None	Improved estimate of population data using higher resolution spatial data New approach to rescaling data	Generally increased pressure scores (0–15 points)
Habitat destruction: subtidal hard-bottom	None	None	

Description of updates to data and methods used to calculate the pressure scores for the global Ocean Health Index 2016 assessment.

### Changes to goal status calculations

Each year’s OHI assessment includes any additional years of data, typically available for many but not all goals (Description of data layers section in S2 File). Additionally, higher-quality data and, occasionally, improved methods for specific goals were included where possible. Such changes were made for the following goals.

#### Food provision, fisheries

Three substantial changes and an additional smaller one were made to the data and analysis for this subgoal. First, the global catch data used to inform spatial patterns of catch were significantly improved through the catch reconstruction project led by *Sea Around Us*, providing catch in tonnes at half-degree grid resolution [13]. At the time of our analysis, catch data were only available through 2010 (in early 2017 data were released through 2013). Future assessments will be updated with the most recent catch data.

Second, we modified our approach to estimating global status of fisheries.

Previously, we used catch-MSY models for estimating stock status [14] in conjunction with fisheries governance scores to determine whether a uniform or constrained prior would be used per stock to inform the data-limited stock assessment model [11]. Further explorations of model outputs with and without the governance information revealed that the addition of a fisheries governance/resilience score did not improve the model outputs. Therefore, we simplified our model and consistently used catch-MSY [9,11] with constrained priors to estimate B/B_MSY_ for all stocks.

Third, where possible, we now replace catch-MSY estimates of stock status (B/B_MSY_) with those from the RAM Stock Assessment Legacy Database (version 3.0) [15]. These RAM data accounted for 13% of global catch in 2010.

Finally, as part of our process to include catches not identified to species level in the overall estimate of stock status within a region, we made a slight modification to how reports of unidentified catch were penalized. Catch is reported across six different levels of taxonomic precision, with the poorest quality being “miscellaneous”. Previously, we assigned a penalty factor of 0.01 to catch reported at this level, but this severely punishes regions with a substantial portion of their catch in this category (i.e., only 1% of this catch counted towards total biomass of fish available as sustainable food). To reduce the impact somewhat, we modified the penalty from 0.01 to 0.1 for catch reported as “miscellaneous”.

#### Biodiversity, species

We made three significant changes/updates to this goal, which relies on IUCN data for species status scores [16] and AquaMaps [17], IUCN [16], and BirdLife International [17][18] spatial data for species ranges. First, we were able to include 1717 more species than the 2013 assessment [11] for a total of 7797 species (2326 more than the 2015 assessment). Overall increase since 2015 was due to changes in the number of species covered by both IUCN (260 fewer species due to fixing previous duplicates) and AquaMaps (1730 new species) and to the inclusion of seabird data from BirdLife International Birds of the World (856 new species). Second, for AquaMaps data we changed the threshold for identifying a species’ range to have a ‘probability of occurrence’ greater than zero instead of the previous threshold of 40% to better approximate the IUCN ‘extent of occurrence’ to make the datasets more comparable [19]. This change led to broader predicted ranges for most species, such that total species count per region increased in most cases (which, in turn, increased the number of both at risk and least concern status species). Finally, where IUCN spatial data included distinct locations for subpopulations (some marine mammals and sea turtles), we assessed risk categories at the subpopulation level rather than for the species as a whole. This change impacted a small fraction of species (12 species with 28 spatially identified subpopulations; 0.15% of included species) but was done to ensure inclusion of the best available information on species status.

#### Sense of place, iconic species

All of the changes described above for species biodiversity were applied to iconic species as well, since iconic species uses a subset of the biodiversity species list. In addition, we supplemented the global list of per-country iconic species for countries bordering the Baltic Sea with lists developed as part of the Baltic Health Index project because the global list did not include species for the Baltic Sea. Furthermore, to calculate trends in species risk, we identified the history of extinction risk from past Red List threat assessments for each iconic species and applied a linear model across the most recent ten-year period to calculate the mean annual change in extinction risk. This latter change provides a much more accurate assessment of the trend in status than the previous approach, which used categorical values of decreasing (-0.5), stable (0.0), or increasing (0.5).

#### Clean waters

We were able to improve our estimate of trends in ocean pollution using newly-available data that estimates the amount of land-based plastic waste entering the ocean [20]. For previous assessments, we inferred trends indirectly from changes in coastal population density.

### Changes to goal trend calculations

We modified the calculation of goal trends to reflect the proportional change in status rather than the absolute change, which is more consistent with the way the overall OHI model is parameterized (Models: likely future status dimensions section in S2 File provides an example calculation that explains the logic). However, this change was relatively minor and had little overall effect on scores. As in previous assessments, we typically calculated trend by estimating the yearly change in status with a linear regression model (i.e., slope estimate) of the five most recent years of status data and multiplied this value by 5 to estimate the change in status five years into the future. In contrast to previous years’ calculations, for the 2016 assessment we divided the slope estimate by the earliest year of data used in the model to estimate proportional change.

### Changes to pressure calculations

Of the 20 pressures tracked in global OHI assessments, we added additional years of data for eight. Data for each pressure were processed into OHI ‘pressure layers’. We also made improvements to how data were processed for 11 pressures (4 of the 8 with additional years of data and 7 others; Table 2). In most cases, the improvements to methods were minor (e.g., small changes to reference points) and had little effect on scores. Furthermore, there were major improvements to source data for fisheries-related pressures and sea level rise. Below we describe changes to the climate change and fisheries pressure layers (additional details provided in Table 2 and S2 File). We also added a new social pressure layer, the Social Progress Index [21], to complement the Worldwide Governance Indicators [22].

#### Ocean acidification

Data used in the ocean acidification pressure layer for 2016 came from a global model of aragonite saturation state (Ω_arag_) at half degree resolution from 2005 through present [23]. Historical levels of Ω_arag_ were modeled from 1880–1889, providing a reference point to calculate change over time. We aggregated these to mean annual values for the years 2005 to 2015.

A biological threshold of Ω_arag_ < = 1 was used as a reference point to rescale the data, recognizing that impacts to marine organisms vary at different levels of Ω_arag_ [24]. All spatially-explicit values currently at or below 1 (i.e., undersaturation of aragonite) were set to 1. All other values were rescaled according to their distance from 1 and their change from historical levels, such that:

$$\Delta \Omega_{year}=\;\frac{\Omega_{base}-\;\Omega_{year}}{\Omega_{base}-1}$$

Note that the current mean annual value of Ω_arag_ (ΔΩ_*year*_) is subtracted from the baseline (mean value between 1880–1889). This causes a reduction in Ω_arag_ to be assigned a higher pressure value (closer to 1). It is then divided by the historical value minus 1 so that if a given pixel has seen a decrease in Ω_arag_, yet the current value remains far from the threshold, the resulting pixel value is indicative of a lower pressure than if the current value was near the threshold. If ΔΩ_*year*_ is negative, indicating a decrease in acidification between measured time periods, the pixel is assigned a pressure value of 0.

#### Sea level rise

The sea level rise pressure layer was derived from monthly time series data clipped to within three nautical miles (nm) of the coast. Previously, a region’s entire EEZ area was used to calculate this layer. Because the impacts of sea level rise were primarily limited to the coast, we have now clipped to three nm.

The main update to this pressure layer was the use of a dataset with higher temporal resolution. The data source remained the same (based on Nicholls and Cazenave [25]), but monthly gridded data of mean sea level anomalies from 1993 to 2015 were used. Previously a single dataset with mean rate of sea level rise from 1993–2012 was used. Monthly data were aggregated for each year from 1993 to 2015. For this most recent assessment, the maximum anomaly value across years 2011 to 2015 was used to rescale the layer from 0 to 1. This improvement allows us to track change over time.

#### Ultraviolet irradiance (UV)

We updated methods to rely on just one dataset that can be used long-term and is comparable through time rather than combining two separate datasets, only one of which is updated. This layer is now calculated in a manner very similar to the sea surface temperature layer (S2 File).

Data come from the Aura OMI Global Surface UVB Data Product [26]. Daily data on Local Noon Erythemal UV Irradiance (mW/m^2^), derived from satellite observations, were aggregated to weekly means for the years 2004 to 2015. Weekly values that were anomalous, i.e. greater than the mean plus one standard deviation, were counted for a total of 52 possible anomalous events per year for each 1-degree resolution raster cell. The total number of anomalous events over the past five years, 2011–2015, were summed and rescaled from 0 to 1 using the 99.99^th^ quantile of the total number of anomalous events over a 5-year period throughout the entire time series.

#### Fishing pressures

The fishing pressure layers have been updated with the new *Sea Around Us* data, which separates catch among four sectors; industrial, artisanal, subsistence and recreational. The two commercial fishing pressure layers (low and high bycatch gear) used spatialized industrial fishing catch data, while the artisanal fishing pressure layer was derived from artisanal, subsistence and recreational data. The commercial fishing catch raster layer was split into two gear rasters indicating catch caught by low and high bycatch gear. This was done by multiplying the industrial catch layer by the proportion of catch caught by low and high bycatch gears. The multiplier dataset was derived from the five fishing pressure layers in previous work [27].

#### Social Progress Index (SPI)

The inverse of the Social Progress Index (1 minus SPI) was added as a new social pressure [21]. The SPI includes several quality of life measures, and is calculated by averaging 3 dimensions, each of which is the average of 4 components. Each component includes several indicators that are scaled from 0 to 100. The SPI is not calculated for any region with 1 or more missing subcomponents. In these cases, we used a linear regression model of the subcomponents with data to estimate the missing subcomponent values. Regions without data were gapfilled with predicted values from a linear regression model that included UN geopolitical region and World Governance Index values as predictor variables. Uninhabited regions received no score.

### Changes to resilience calculations

The primary change to how resilience measures were calculated was to restructure the resilience data so the resilience layers within the regulatory category were each explicitly included in the resilience matrix and identified as addressing one of the five ecological pressure categories (pollution, alien species, habitat destruction, fishing pressure, and climate change; Models section in S2 File). Previously, the association between resilience and pressure layers was obscured because resilience variables within a category were averaged prior to calculating OHI scores and the pressures they addressed were not identified. These changes had minimal effects on the scores, but improved transparency and make calculations more easily understood. We also added the Social Progress Index as a resilience measure (described above). Only three of 14 resilience data sources had additional years of data available: species condition (used for artisanal opportunities and natural products goals and habitat, fisheries, and iconic species subgoals), marine protected areas (used for all goals except, clean waters, tourism and recreation, and livelihoods and economies), and World Governance Index.

### Patterns and trends in scores

To analyze global trends in Index and goal/subgoal scores, we used linear mixed effects models to evaluate the change in scores from 2012 to 2016, with region (country or territory) included as a random effect. We calculate average annual change in scores as the mean of region scores regardless of EEZ size (unweighted) or as an area-weighted average (weighted). Statistical analyses were performed using the nlme package [28]. Final models were computed using the restricted maximum likelihood (REML) estimation [29].

### OHI as an indicator

One major way that the Ocean Health Index framework incorporates sustainability into the assessment of each goal is by estimating where the status of the goal is likely to be in five years (termed ‘likely future status’). Likely future status is estimated by combining data on the recent trends in the status of a goal, current pressures on the goal from human activities, and existing governance and resilience measures that help support delivery of the goal (Models section in S2 File). For the first time we can begin to test how well this methodology performs by comparing the likely future status from the first year of assessment (2012) to the current status of the fifth year (2016). Limitations in the frequency with which input data are updated constrain these analyses, but we were still able to test this relationship in several ways. For overall Index scores and each goal/subgoal we used a series of linear regression models to evaluate which components of the OHI model help predict future status. Specifically, we compared the observed 2016 status to the predicted status in 2012; observed change in status (2016 minus 2012 status scores) to the predicted change (2012 likely future status minus 2012 status); and the observed change versus various combinations of components of the model (i.e., trend, pressure, resilience, and resilience minus pressure).

Given the popular focus on country rankings as an outcome of global indicator assessments, we also compared how region scores compare to region rankings over time and how changes in scores relate to changes in ranks. To better understand the relationship between rank score and absolute score, we used linear regression models to compare change in goal scores and goal ranks.

## Results

### Patterns and trends in scores

**Fig 1:**
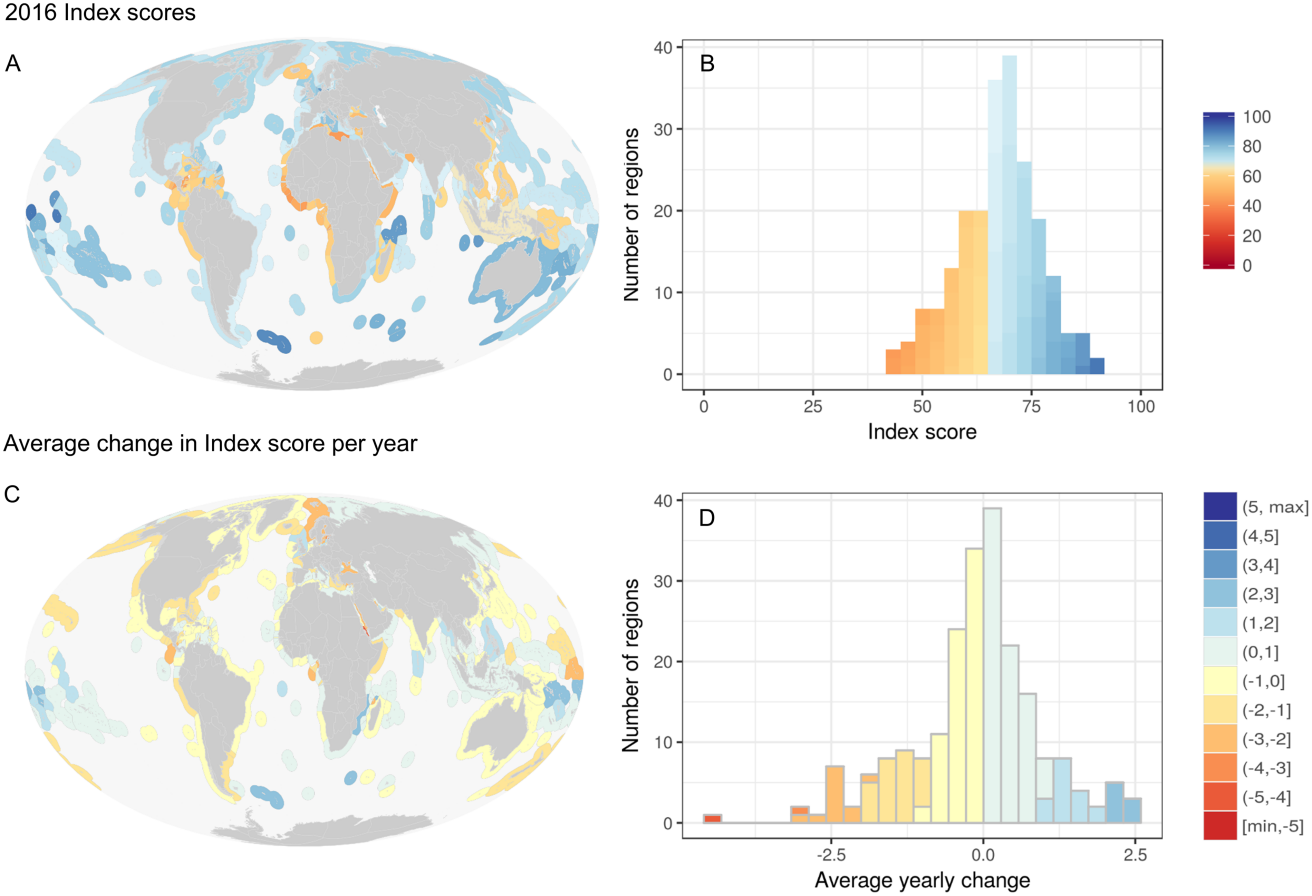
Map and distribution of OHI Index scores and average yearly change in scores. (A) Map of 2016 per-region scores shows lowest scores generally in tropical areas and highest scores generally in South Pacific and Southern Oceans. (B) Distribution of per-region scores is normally distributed around the global OHI score of 71. (C) Map of per-region average yearly change in Index scores from 2012 to 2016 (based on linear regression analysis of Index scores), and (D) distribution of average change among regions.

The global mean area-weighted Index score for 2016 was 71 out of 100 (median 68, per-region range 43–91; Fig 1B, Figure A in S1 File). The two top scoring regions, Jarvis Island and Howland & Baker Islands (both 91), are remote and uninhabited islands; the highest scoring populated areas were Germany (85) and Seychelles (84; Fig 1A, Datasets and additional information section in S1 File provides links to data). The lowest scoring regions were Libya and Sierra Leone (both 43). Nine of the 10 lowest scoring regions are in Africa and the other in Central America (Nicaragua). Global scores for goals and subgoals were highest for biodiversity and coastal protection and lowest for natural products and tourism & recreation, but nearly all goals (Figure B in S1 File) and subgoals (Figure C in S1 File) showed the full range of possible values among the 220 assessed regions.

**Fig 2:**
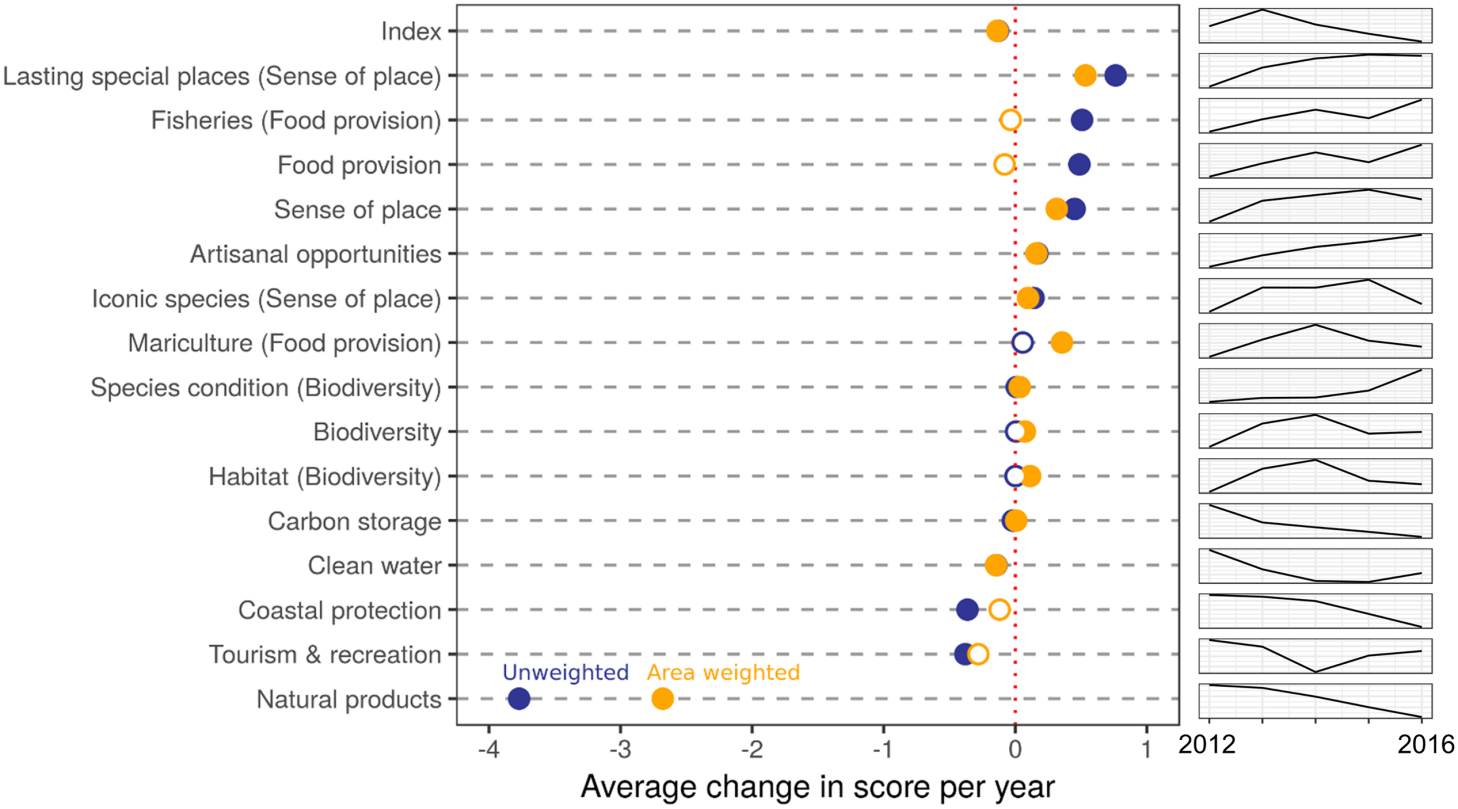
Global average yearly change in goal scores from 2012–2016. Average annual change in global status for each goal and subgoal, unweighted (blue dots) and weighted by size of EEZ (orange dots). Solid circles indicate trends significantly different from zero; open circles are non-significant. Plots on the right show change over time in the global goal score (y-axis scaled to the range of values for each goal). Large differences between unweighted and weighted values (e.g. natural products and fisheries) result from countries with large EEZs having scores significantly different from the global average.

The global score remained unchanged from previous years, although individual regions increased by up to 2.5 points per year (e.g., Mozambique, Samoa, Solomon Islands) or decreased by as much as 4.5 points per year (e.g., Eritrea -4.4, Estonia -3; Fig 1C), with a roughly normal distribution of values of per-region change (Fig 1D). Most goal scores changed significantly over the five-year period but with relatively small absolute changes (Fig 2). The largest increase was for the lasting special places goal (nearly +1 point increase per year, unweighted) and the largest decrease was for the natural products goal (nearly -4 point decrease per year, unweighted). Natural products, coastal protection, and carbon storage all saw steady declines over the five years (although coastal protection was not significant for the area-weighted average); lasting special places, fisheries, artisanal fishing opportunities and species biodiversity all saw steady increases over the five years (although fisheries was not significant for the area-weighted average); and the other goals varied over time (Fig 2).

**Fig 3:**
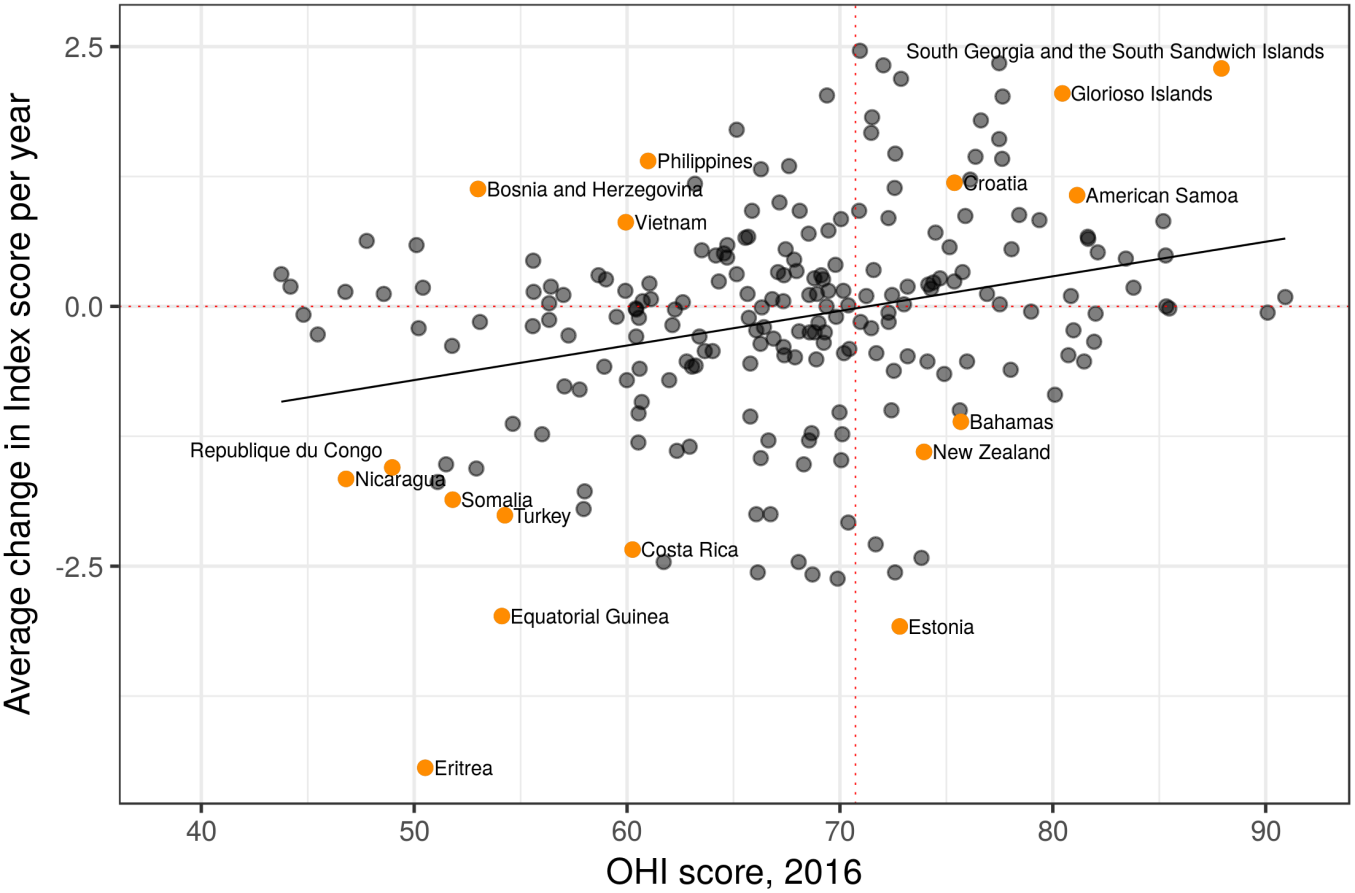
Relationship between score and annual change in score. OHI scores for 2016 versus the annual change in score over 5 years for each region. Red dashed lines indicate no change over time (horizontal line) and the mean Index score across regions (vertical line); dark black line is the linear regression slope. Regions with higher Index scores in 2016 that improved through time are in the top-right quadrant, and countries with lower scores that declined through time are in the bottom-left. Data points for labeled countries are colored orange for ease of identification.

**Fig 4:**
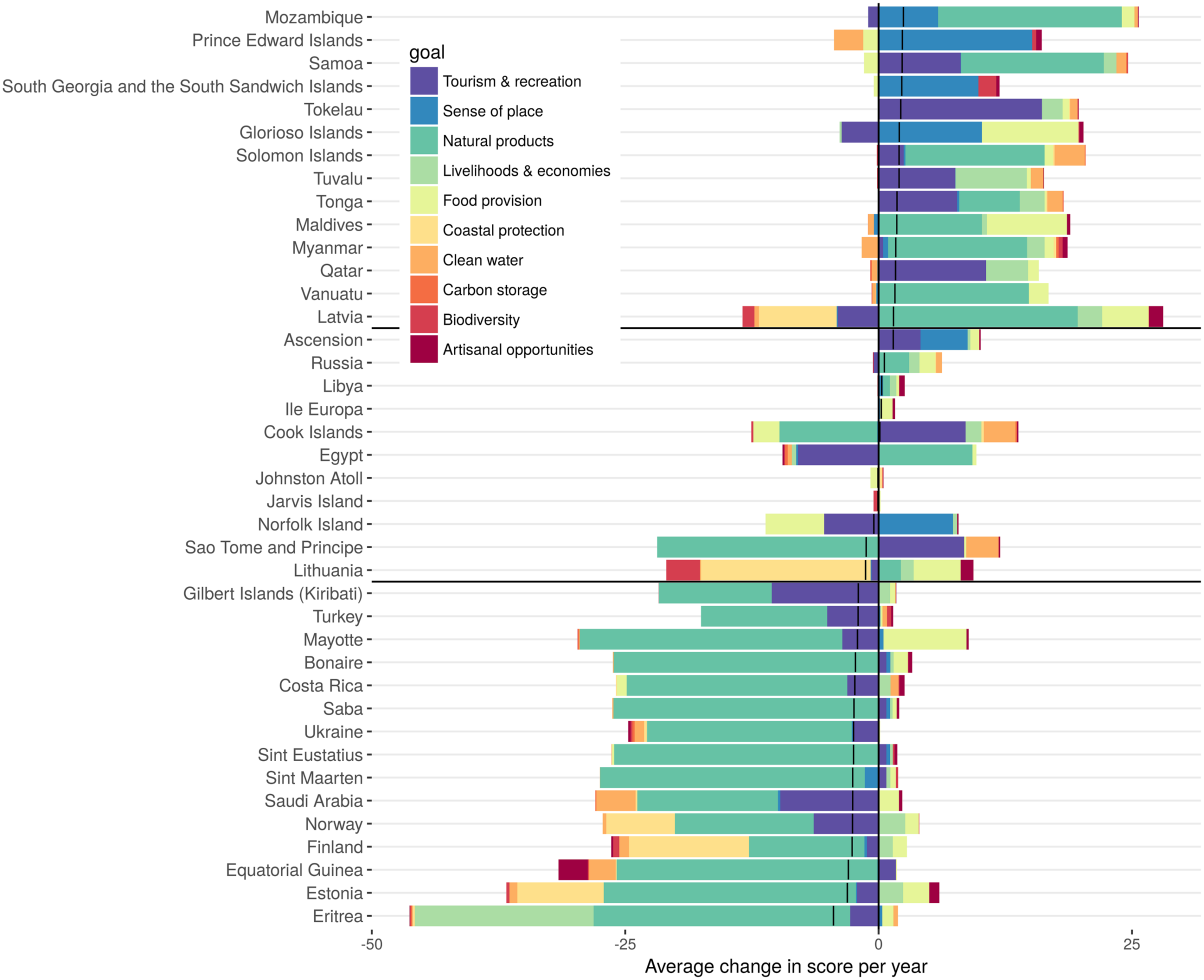
Drivers of change in OHI scores from 2012–2016 for a sampling of regions. Contribution of OHI goals to changes in annual Index scores for the 15 regions with largest increases and decreases in scores and 10 representative regions in between (separated by dark black lines). Light dark lines are the overall trend. Colored bars are the magnitude of change in each goal, either positive (to the right of the heavy black line) or negative (to the left). Note that minor change in an Index score can result from a wide range of possible combinations of changes in goal scores. See Figure G in S1 File for all countries.

In general, regions with higher Index scores in 2016 tended to have improved over time (i.e., the good get better) and regions with lower scores tended to have worsened (p<0.001, linear regression model of change per year ~ Index score), although there were plenty of exceptions (R^2^ was only 0.08; Fig 3). Very few regions started with low scores and improved dramatically or had high scores and declined dramatically. Roughly half of all regions had scores decline and half had scores increase (Fig 3). Improvements in natural products, sense of place and tourism & recreation drove overall improvement in the regions, with the highest increase in overall Index score, while declines in natural products and, to a lesser degree, tourism & recreation were the primary drivers of the greatest declines in regions (Fig 4; Figures D-G in S1 File). Minor changes in region-level Index scores were the result of a wide range of possible combinations of changes in goal scores. For example, a minimal change in all goals or large changes (but in opposite directions) of two or more goals (Fig 4, Figure G in S1 File) could both lead to only a minor change in a region’s overall score.

### OHI as an indicator

**Fig 5:**
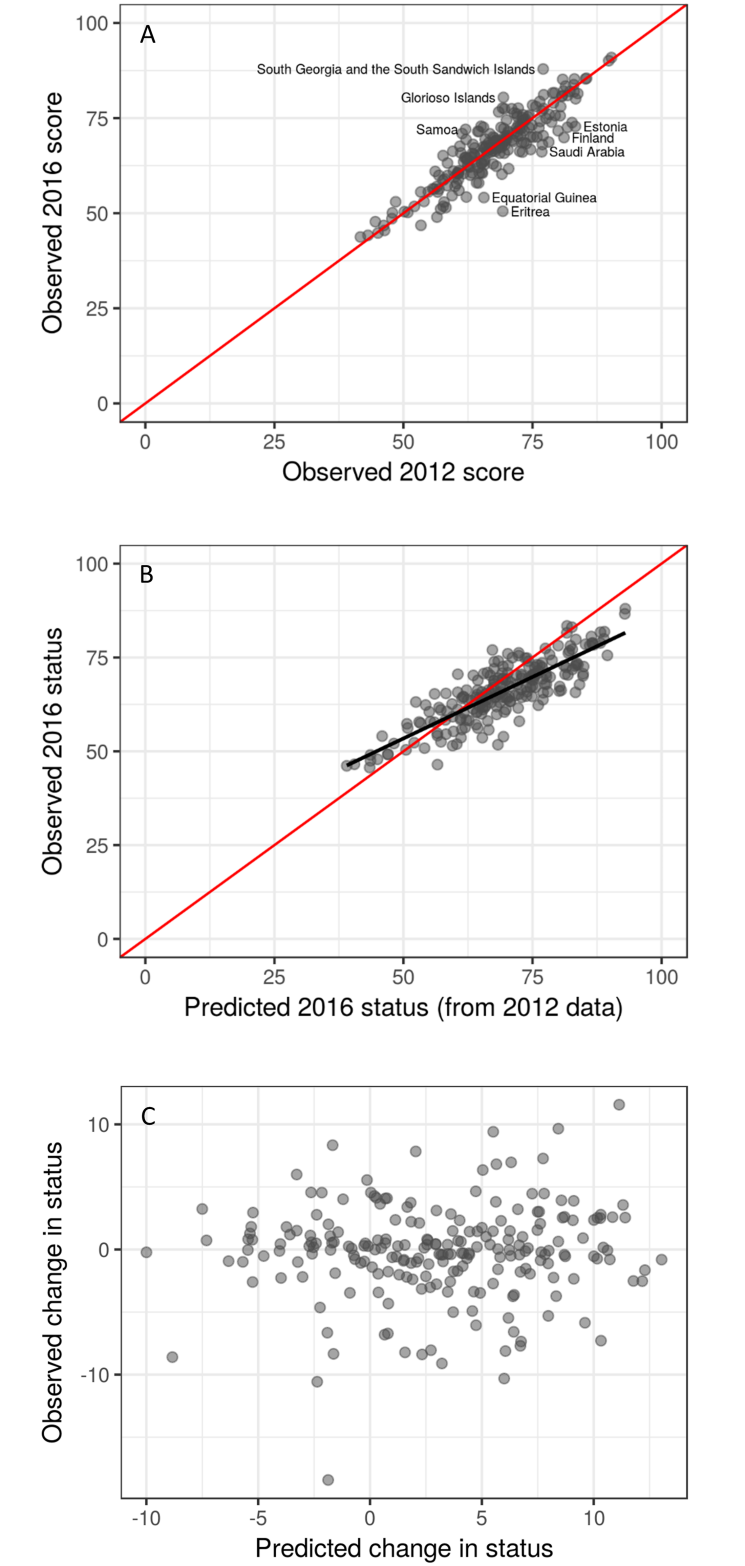
Evaluating the OHI model using 5 years of data. Relationship between different aspects of OHI scores. (A) OHI scores in 2012 versus 2016, showing past scores predict future scores; (B) ‘likely future status’ in 2012 (i.e., predicted status in 2016) versus observed status in 2016, with black line indicating the slope estimate from a linear regression model; and (C) expected change in status (OHI status minus ‘likely future status’ from 2012 scenario) and the observed change (status in 2016 minus status in 2012). Red lines indicate the one-to-one relationship.

Results were variable for how well different components of scores in 2012 predicted current status in 2016. Overall, there was a strong, highly significant relationship between overall scores in 2012 and 2016 (Fig 5A; linear regression model, p < 0.001, R^2^ = 0.79). In other words, past scores are a really good predictor of future scores. This makes sense as overall Index scores are not expected to change much from year to year. The few exceptions (South Georgia and the South Sandwich Islands, Glorioso Islands and Samoa had score changes ≥ 10 points; and Estonia, Finland, Saudi Arabia, Equatorial Guinea, and Eritrea had changes ≤ -10 points) resulted from very large changes in a few goals (see results described above). The relationship between the ‘likely future status’ in 2012 and ‘current status’ in 2016 was still strong and highly significant (Fig 5B; linear regression model, p < 0.001, R^2^ = 0.70), but weaker than the relationship between the overall scores, indicating that inclusion of the ‘likely future status’ reduces predictability. Higher-scoring regions tended to have overly optimistic predicted status (points below the red 1:1 line, Fig 5B), whereas lower scoring regions tended to underestimate future status (points above the red 1:1 line, Fig 5B). To directly address the ability of ‘likely future status’ to predict realized future ‘current status’, we compared predicted changes to observed changes (see Methods above) and found a non-significant, weak relationship (Fig 5C; linear regression model, p = 0.357).

Results at the goal and subgoal level were mixed (Table C and Figure H in S1 File). There was a significant relationship, based on linear regression models, between predicted and observed change in status for habitat biodiversity (p = 0.046), mariculture (p < 0.001), and tourism & recreation (p = 0.001), suggesting the overall OHI model was effective for these goals. In other cases, individual or combinations of the components used to predict likely future status (trend, pressure, resilience) were significantly correlated with observed change in current status, suggesting that the OHI model was partially effective in these cases. Trend alone was predictive of changes in fisheries current status (food provision goal; p < 0.001). Resilience was predictive of the change in natural products current status (p = 0.042). There was a significant relationship between trend and resilience for artisanal opportunities (p = 0.009 and 0.019, respectively). The difference between resilience and pressure measures was correlated with changes in lasting special places current status (p = 0.017). For many goals, however, insufficient temporal data precluded robust estimates of likely future status. This was demonstrated by no observed change in status from 2012 to 2016 for the carbon storage goal and species biodiversity subgoal, and a change in status for only a few regions for the habitat subgoal (only sea ice and soft-bottom habitat data change) and the coastal protection subgoal (only sea ice habitat data change). Furthermore, there were no changes to the data for the livelihoods and economies goal after 2013. It was, thus, impossible to evaluate the model in these cases.

**Fig 6:**
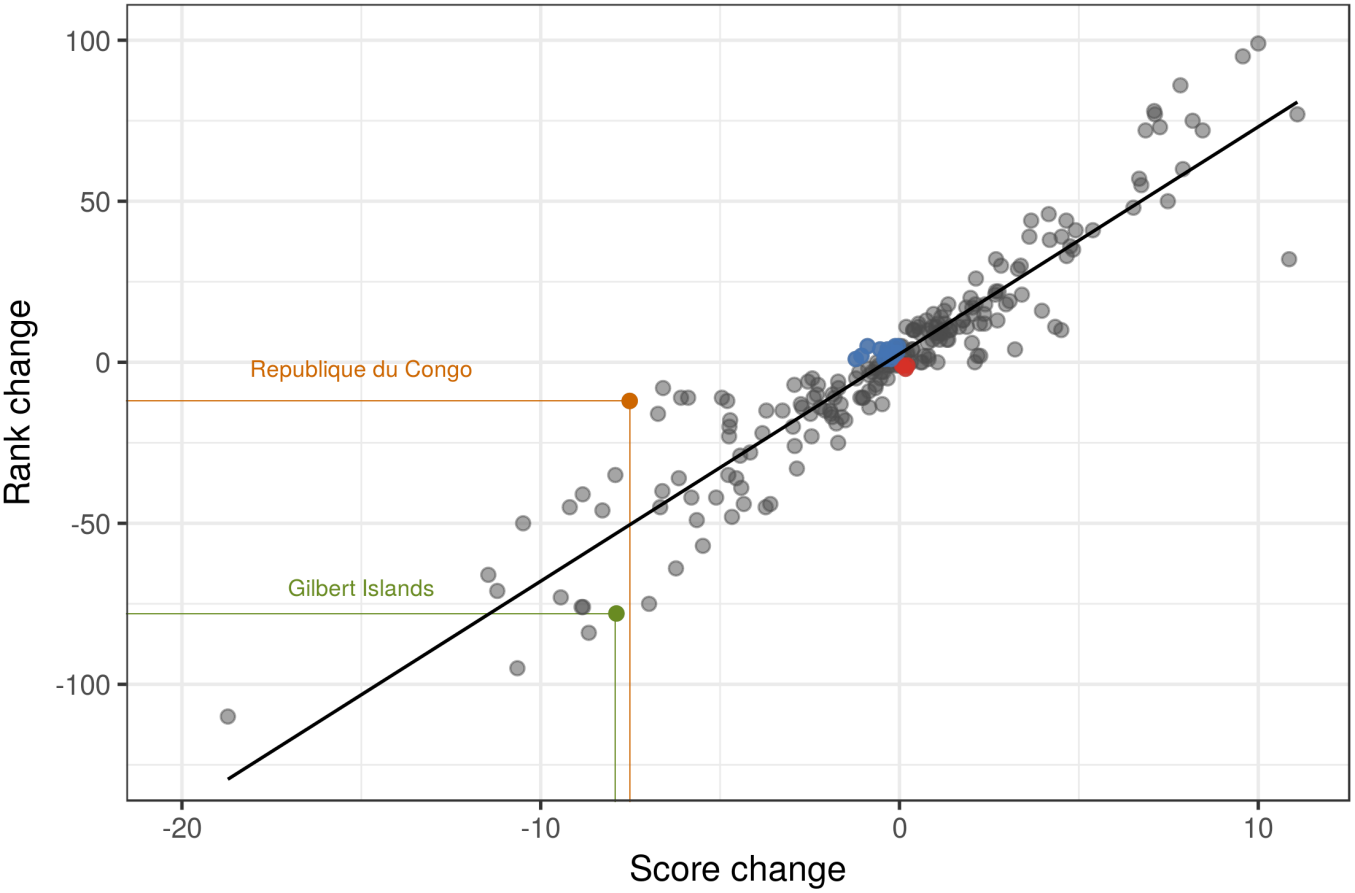
Relationship between change in OHI score and rank. The change in each country’s OHI score and rank was calculated from 2012 to 2016. Black line is the linear regression slope estimate. A comparison of the Republique du Congo and Gilbert Islands (Kiribati) illustrates how roughly the same change in score (-7.52 vs. -7.89, respectively) can result in a very different change in rank (-12 vs. -78); note also cases where the same change in rank can result from very different changes in scores (not highlighted). Blue points indicate regions that had a decrease in score but an increase in rank (N = 12); and red points indicate an increase in score and decrease in rank (N = 4).

Changes in scores were strongly correlated with changes in rank order, with greater deviation from predicted shifts with more positive or negative changes (Fig 6, Figures I and J in S1 File). However, many regions had large shifts in either score or rank but not the other. For example, South Georgia and South Sandwich Islands increased in OHI score by nearly 11 points but only increased its rank 31 steps (much less than the relationship predicts). The fact that the same change in score can result in very different changes in rank is in part due to the underlying distribution of scores not being uniform and further highlights how rank order is not an ideal indicator of ocean health and can be misleading. For example, five different regions had Index scores decrease by roughly 7 points but had rank order decline by very different amounts: 8 (Nicaragua), 16 (North Korea), 40 (Faeroe Islands), 45 (Mayotte), and 74 (Sweden). Furthermore, score and rank can change in opposite directions (see Fig 6), for example in Sudan absolute scores declined -1 while the rank improved +4.

## Discussion

### Patterns and trends in scores

The last five years have seen notable improvements in some aspects of ocean health and worrisome declines in others, with substantial variation among different regions (countries or territories) around the world. As expected, most regions with the greatest increase (>1 point per year on average) or decrease (>-2 points per year on average) in scores over the five-year period were those with mid-range scores, i.e., in the 60s or 70s (Fig 3). Regions with higher scores have less room to improve due in part to diminishing returns from efforts and generally have sustainability measures (regulatory, ecological, and social resilience) in place to maintain high scores, whereas regions with very low scores have less room to worsen and likely have fewer institutional systems in place to help improve conditions. The absence of regions in the upper left and lower right corners of Fig 3 further emphasizes this point.

Most of the biggest changes in scores (both negatively and positively) were driven in large part by changes in scores for the natural products goal (Fig 4, Figure G in S1 File). This goal measures the sustainable harvest of natural products within a region. Globally we track this goal with harvest data on any of six products reported to the Food and Agriculture Organization (FAO) of the United Nations (sponges, shells, fish oil, corals, ornamental fish and seaweeds). Estimates of the sustainability of harvest of any product are sensitive to the quality of data reported to FAO, as well as to the underlying assumption that the highest harvested quantity over the time series of extraction is a reasonable proxy for maximum sustainable yield (this assumption is borrowed from fisheries models on catch data).

Many regions are extracting fewer natural resources relative to their historical peak. For example, the natural products score of the United States decreased by an average of about 9 points per year from 2012 to 2016. This was primarily due to a substantial decrease in fish oil harvest (e.g., 183,290 tons were harvested in 1983 and 65,849 in 2013), however, there were also decreases in seaweed, sponge, and coral products. Costa Rica’s natural products score decreased by an average of about 22 points per year due to consistent declines in the extraction of ornamental species. Many regions may be extracting relatively fewer natural products because peak extraction levels were unsustainable, resulting in increased extraction costs or improvements in regulations. Alternatively, regions may be optimizing other, more profitable goals, such as tourism and recreation if there are trade-offs between these goals and the natural products goal. Some regions had large decreases (or increases) in the natural product goal simply because product extraction is small, making the sustainable harvest model sensitive to small changes. We included several measures to mitigate this model behavior (e.g., only including products with at least 4 years of extraction data, using a 35% buffer for the reference point, and using a 5-year running average), but could not fully address this model sensitivity. For example, Eritrea’s natural product score went from 100 in 2012 to 5 in 2016 due to decreased extraction of ornamentals. However, the extraction of ornamentals from this region has been very low (average of about 0.5 tons since first record in 1995) and sporadic (about 50% of the years have 0 extraction according to FAO data).

Notable changes in goal scores also occurred with increases in the lasting special places subgoal (sense of place goal) and the fisheries subgoal (food provision goal) and decreases in the coastal protection and tourism & recreation goals (Fig 2). Increases in the lasting special places subgoal were due to substantial numbers of MPAs having been created around the world in the last five years, largely in response to commitments to the United Nations to protect at least 10% of waters within each country’s exclusive economic zone (EEZ) by 2020 [30].

Increases in the fisheries subgoal, while modest (0.5 point per year on average), represent another global success story for ocean health because fisheries, especially in developed countries where stocks are being harvested more sustainably. Of the 220 regions we assessed, 142 had increasing fishery status scores, which is driven by improving sustainability scores (e.g., B/B_MSY_). Status scores can improve for a region if B/B_MSY_ values increase over time for stocks with B/B_MSY_ values less than 1 (indicating potential overharvesting) and/or decrease for stocks with B/B_MSY_ values greater than 1 (indicating under-harvesting). Increases in Norway’s and Canada’s fishery status scores were driven by both increasing B/B_MSY_ values when low and decreasing B/B_MSY_ values when high. Increases in Vietnam’s and Madagascar’s status scores were driven primarily by decreases in the B/B_MSY_ values of under-harvested stocks. Substantial challenges remain for reforming management and harvest of fisheries in many parts of the world, particularly in developing countries, but the improvements we found echo the message of other studies that concerted and enforced management of sustainable catch and effort levels can produce abundant and sustainable seafood.

Declines in scores for coastal protection were primarily driven by the substantial loss of coastal sea ice in sub-Arctic coastlines. Sea ice is one of the five assessed habitats that help protect coastlines from storm damage and erosion (the others are seagrasses, coral reefs, mangroves and salt marshes), and it is the only one for which high spatial- and temporal-resolution data on habitat extent exist. Six sub-Arctic countries (Lithuania, Sweden, Finland, Estonia, Latvia, Norway) had coastal protection scores drop by more than 25 points from 2012 to 2016 due to sea ice loss, and these changes in turn drove the decline in the global score for coastal protection.

Global declines in scores for tourism & recreation are not as easy to explain. In the absence of more direct global data on the number of tourists visiting coastal areas of each region, we used employment data from the travel and tourism industries to model likely changes in coastal tourism. Declines in such jobs over the past five years are likely real, but the causes of those declines and how they might relate to, or impact, the tourism and recreation industries are much less certain. Ideally, the tourism & recreation goal would be assessed by information more directly connected to the benefits people enjoy through access to marine tourism and recreation, such as with data on participation in tourism and recreation activities [31], but these data are not available at a global scale and employment data is already a more direct measure than was used in previous assessments [9].

One of the key strengths of the OHI framework is the ability to break scores into their component, comparable parts, which facilitates exploration and understanding of potential tradeoffs, synergies or other interactions among those parts while still allowing one to combining data into a single measure. For example, most regions with the greatest increase in overall score had nearly all goal scores increase (Fig 3), but Latvia, for example, had several goal scores increase substantially while others decreased notably. This combination led to only a small overall increase in OHI score. Interestingly, for many regions a wide range of possible combinations of goal score changes led to little change in OHI scores (Fig 4), highlighting how different interactions among goals can produce the same overall score. For example, the score for Norfolk Island stayed the same across 5 years (81), but during this time, the sense of place goal increased from 57 to 94 due to the creation of a large MPA while sustainable harvest of fisheries declined (due to decreases in median B/Bmsy scores), which lowered the food provision score 20 points, and tourism and recreation scores decreased by 19 points due to decreases in sustainability and employment metrics for tourism.

### OHI as an indicator

One of the main challenges for indicators that aim to address sustainability is the need to account for future conditions (a prerequisite for something to be ‘sustainable’) while not actually being able to model the future since an indicator, by definition, indicates current conditions. The OHI framework addresses this challenge by incorporating data on recent trends and existing pressures and resilience measures to suggest where things are likely headed in the near future. For the first time we could evaluate how well this aspect of the framework performs.

As expected, past scores generally predict future scores (Fig 5A). In other words, scores changed slowly over relatively short time periods, primarily because overall ocean health is not something that generally can change rapidly. An unexpected pattern that emerged when comparing the predicted status for 2016 (i.e., the ‘likely future status’ in the 2012 assessment) to the observed current status (measured in 2016) was that the predicted status for high-scoring regions tended to be overestimated but for low-scoring regions was underestimated (Fig 5B). This suggests our model for likely future status generally overestimates the predicted amount of change in future scores. This result is in part driven by the result that higher-scoring regions tended to get better over the five years of assessment while the lower-scoring regions tended to get worse (Fig 3), which in turn creates large positive or negative trends, respectively. Trend scores in turn have a strong influence on the likely future status score.

Because the OHI framework gives equal weight to the likely future status and current status, inclusion of an estimate of the likely future status into indicator scores creates the potential for initial changes in goal scores to overestimate future changes in goal scores, especially as scores approach limits (e.g., best or worst possible scores). This effect is most evident in our most recent 2016 assessments of the lasting special places subgoal, which is primarily driven by changes in total area protected within MPAs. When regions create an MPA, especially a large one, the current status for the subgoal goes up the next year, creating a positive trend that is used in the Index calculations to indicate the likely future status (which assumes that the trend will continue the following years). If a region does not create additional MPAs the following years (or they reach the target reference value), then the trend will overpredict the likely future status. Over time, as the trend flattens out (i.e., when the current status is stable at the reference value), the likely future status equilibrates with the current status, such that this issue ‘resolves’ itself.

For habitat biodiversity, mariculture, fisheries, tourism & recreation, and artisanal fishing opportunities, there was a significant relationship between the 2016 assessment’s observed change in status (current status in 2016 versus 2012) and the 2012 assessment’s predicted change in status (which includes trend, pressures and resilience measures). For artisanal opportunities and fisheries, trend was correlated with the change in status (and resilience for artisanal opportunities), and for lasting special places resilience and pressure were correlated (Table C and Figure H in S1 File). These goals/subgoals had sufficient temporal data to allow a robust test of the relationships, and the results suggest the method works when data are available. The OHI model did not perform well for the clean waters, iconic species, and natural products goal, a result that requires additional research to understand but one that is likely due to issues with the quality of input data. Unfortunately, the other goals and subgoals (species condition, coastal protection, carbon storage, and livelihoods and economies) simply do not have sufficient temporal data to test these relationships. For example, the same species risk status data, which is the main component of species biodiversity score, is used for every scenario year.

Finally, some critique of the OHI has focused on unexpected order of country rankings, especially for the fisheries subgoal [32]. Absolute values for goal scores are a more useful indicator of condition than rank order, especially when tracking change over time, but to understand the relationship between the two we made several comparisons (Fig 6; Figures I and J in S1 File). As expected, a change in score positively correlates with a change in rank, but the number of outlier points and the fact that the same absolute change in score can result in dramatically different changes in rank order suggest caution when focusing on rank order. These mismatches are particularly stark when comparing changes in scores to changes in ranks for many of the specific goals and subgoals (Figures I and J in S1 File). Importantly, rank order is a less useful indicator for decision-makers than absolute change in score, especially within a specific country context, and can even give false information on the effectiveness of decision-making if changes in rank go the opposite direction of changes in scores.

### Lessons learned from repeated indicator assessments

For indicators to be useful tools for tracking progress towards policy objectives, they need to be evaluated regularly and updated with the best available data and science. For indicators to be rigorous and defensible scientifically, the underlying data and the methods used to analyze those data need to be fully transparent. These needs create substantial practical challenges for indicators, especially global indicators, which have to be based on existing data that are freely and repeatedly available. Our efforts to calculate the OHI each year over half a decade help highlight specific issues related to these challenges.

Many data sources are updated regularly (annually or biannually), such as country level statistics reported to FAO, making them ideal for use in global indicators. However, data sources often change over time. For example, changes in technology (e.g., new satellites with different sensors) can lead to a new, higher-quality data source that ideally should be incorporated into indicator assessments, but the new data often cannot be directly compared to the old data, limiting or even prohibiting comparisons across time. We encountered this situation with the change in satellite measuring UV radiation, which created a new data format that could not easily be compared to past UV measurements. Should an updated assessment of an indicator switch to using the new, better data (i.e., the best available science) but forego the ability to track change over time, or should it forego using the newest, best data in order to be able to continue tracking change over time? In some cases, the choice is forced, as when the old data are discontinued once the new data become available.

More problematic is when existing, useful data are simply discontinued without any meaningful replacement being available. We encountered this challenge with the FAO data on commercial fishing and mariculture employment (last provided for 2012 from personal communication, but last publically available for 1996) and fishing revenue data (last provided for 2007 fisheries). The marine wage data from the International Labour Organization that we used to calculate the livelihoods subgoal was also discontinued in 2014, and replaced with information too coarse to be able to determine ocean-related wages. Unfortunately, no other data exist for national level statistics on commercial fishing and mariculture livelihoods & economies, prohibiting us from being able to update these calculations in 2015 and 2016 assessments. Since such data are pertinent to national politics and economics, it is imperative that more data be collected and tracked. Such data would enable future OHI assessments to better reflect changes in livelihoods and economies.

Another key data challenge is that many of the ‘regularly updated’ data are not updated annually or do not include all regions. We address these gaps in data with various methods [8], all of which require assumptions and well-informed, but ultimately subjective, decisions that sufficient information exists to move forward (or, conversely, to abandon potentially useful data if it seems that too many gaps exist). These issues of data inconsistency (or absence) are profoundly important because agencies and organizations cannot effectively manage what has not been measured. For regions that have gap-filled data, regional and global patterns could conceal local problems that need mitigation or local successes that should be acknowledged and built upon.

A less obvious but equally challenging problem with repeating and updating assessments is that new data, or even just additional years of data, can change our understanding or assessment of what the sustainable reference point for a given goal should be [33], either for objective reasons (e.g., improved understanding of biophysical constraints to sustainable delivery of a goal) or subjective reasons (e.g., modified societal goals for ideal goal status). These reference points have a large influence on the scores of individual regions because they determine what a score of 100 means and are thus what all scores are scaled to. Many examples of this issue exist within OHI.

With emerging fisheries, where catch is increasing each year, data limited stock assessment models (based on catch data) update B/B_MSY_ values each year. This same issue exists for the natural products goal for each of the six natural products assessed. In other words, new data showing higher catch/harvest values are used to re-calculate population parameters (e.g., B/B_MSY_) that drive reference points. For global pressure data, such as sea surface temperature anomalies or land-based pollution run-off, reference points are set to the highest observed value over the time series (or some other high quantile value) under the assumption that this is the highest possible value that could ever be observed. Thus, additional years of data can create new maximum values that require rescaling all previous values. In these cases, pressure scores would appear to decrease, not because pressures had actually decreased but because they were being rescaled to a higher reference point based on our increased understanding of the maximum possible pressure. Incorporating these changes is appropriate and important to do, but they create unique challenges in communicating how and why scores are changing.

Finally, repeated assessments require sustained support for analytical teams to produce consistent and comparable results. Continuity of team members and the institutional memory they create can help make these assessments efficient, but efficiency can also be achieved if collective memory is captured directly into reproducible analytical workflows, allowing new (and veteran) team members to pick up where others left off [10]. These workflows also help promote transparency, which is critical for indicators to be adopted and used by policy makers.

## Conclusions

Globally, observed changes in OHI scores do not suggest the oceans are dying, but nor are they thriving. Thus, there is cause for optimism, but also for concern. The OHI provides one key tool for understanding how ocean health is changing in a given region as well as indications of why those changes are happening, what their implications are for natural and human systems, and what actions might be taken to improve ocean health. Smaller-scale, tailored assessments at the scale of decision making that leverage the best available science and data offer even higher quality information and guidance [10] (ohi-science.org).

Our results emphasize the central importance of good governance to ocean health. Regions with stable and effective governance tend to score much higher than regions where corruption, dictatorship, civil strife, war and poverty have been chronic (Table D and Figure L in S1 File). This finding underscores the fact that marine scientists, managers and policy makers cannot alone improve ocean health. Doing so will require efforts from all sectors to promote peace, justice, gender equality, socially-responsible business and other aspects of civil health, because progress in those areas makes it much easier for communities and nations to improve the environmental and economic conditions needed to boost ocean health.

## Supporting information

S1 File Supporting results. Additional analyses, figures, and tables.
(HTML)

S2 File Supporting methods. Description of OHI philosophy and methods.
(HTML)
