# Supplementary material for: Drivers and implications of change in global ocean health over the past five years
Source: PLoS One. 2017 Jul 5;12(7):e0178267. doi: 10.1371/journal.pone.0178267 (PMC5497940; doi:10.1371/journal.pone.0178267)
Supplement: S1 File — Additional analyses, figures, and tables. (HTML) [file pone.0178267.s001.html]

S1 Results


OHI Science | Citation policy


Code 

- Show All Code
- Hide All Code

# S1 Results

### Overview of goals and subgoals

##### Table A. Description of 10 goals used to calculate OHI scores.

In several tables and figures we refer to the goals and subgoals by their abbreviation. Goals and subgoals have a 2 and 3 character abbreviation, respectively.

| Goal | Abbreviation | Description |
| --- | --- | --- |
| Food Provision | FP | The sustainable harvest of seafood from wild-caught fisheries and mariculture |
| Artisanal Fishing Opportunity | AO | The opportunity for small-scale fishers to supply catch for their families, members of their local communities, or sell in local markets |
| Natural Products | NP | The natural resources that are sustainably extracted from living marine resources |
| Carbon Storage | CS | The condition of coastal habitats that store and sequester atmospheric carbon |
| Coastal Livelihoods and Economies | LE | Coastal and ocean-dependent livelihoods (job quantity and quality) and economies (revenues) produced by marine sectors |
| Tourism and Recreation | TR | The value people have for experiencing and enjoying coastal areas through activities such as sailing, recreational fishing, beach-going, and bird watching |
| Sense of Place | SP | The conservation status of iconic species (e.g., salmon, whales) and geographic locations that contribute to cultural identity |
| Clean Waters | CW | The degree to which ocean regions are free of contaminants such as chemicals, eutrophication, harmful algal blooms, disease pathogens, and trash |
| Biodiversity | BD | The conservation status of native marine species and key habitats that serve as a proxy for the suite of species that depend upon them |
| Coastal Protection | CP | The amount of protection provided by marine and coastal habitats serving as natural buffers against incoming waves |

##### Table B. Description of subgoals used to calculate OHI scores.

The following goals are comprised of 2 subgoals:biodiversity, food provision, sense of place, livelihoods and economies goals.

| Subgoal | Goal | Abbreviation | Description |
| --- | --- | --- | --- |
| Fisheries | Food provision | FIS | The sustainable harvest of seafood from wild-caught fisheries |
| Mariculture | Food provision | MAR | The sustainable harvest of seafood from mariculture practices |
| Habitat | Biodiversity | HAB | The status of key habitats that serve as a proxy for the suite of species that depend upon them |
| Species condition | Biodiversity | SPP | The conservation status of native marine species |
| Iconic species | Sense of place | ICO | The conservation status of iconic species (e.g., salmon, whales) that contribute to cultural identity |
| Lasting special places | Sense of place | LSP | The conservation status of geographic locations that contribute to cultural identity |
| Livelihoods | Coastal livelihoods and economies | LIV | Coastal and ocean-dependent livelihoods (job quantity and quality) produced by marine sectors |
| Economies | Coastal livelihoods and economies | ECO | Coastal and ocean-dependent economies (revenues) produced by marine sectors |

### Datasets and additional information

For more information about the models used to calculate OHI scores as well as the underlying data see the S1 Methods document.

CSV formatted data from the 2016 assessment are available. These data include index and goal/subgoal scores (as well as the dimensions used to calculate scores: status, trend, pressure, resilience) for all 5 assessment years (2012-2016).

Simplified versions of the full dataset, which include only the final index scores, are available for the 2012-2016 scenarios: 2016, 2015, 2014, 2013, and 2012.

The data layers (described in section 6 in S2 Methods) and functions used to calculate scores can be downloaded from Github: ohi-global v2016.1: Global scenarios data for Ocean Health Index.

The files used to prepare data layers for the ohi-global assessment can be downloaded from Github: ohiprep v2016.1: Preparation of data for 2016 Ocean Health Index global assessment.

The core functions used to calculate OHI scores can be downloaded as a package from Github, using the following code in the R:

```
install.packages('devtools')
library(devtools)

install_github('ohi-science/ohicore')
library(ohicore)
```

### Summary of score results

##### Figure A. Map and flowerplot of 2016 OHI index scores.

The map (top) describes the overal index scores for each OHI region. The flowerplot (bottom) describes the global average scores for each goal/subgoal, weighted by each region’s eez area.

##### Figure B. Maps and histograms of 2016 OHI goal scores.

The scores for each of the 10 OHI goals displayed for each region (left) and as a histogram (right).

##### Figure C. Maps and histograms of 2016 OHI subgoal scores.

The subgoal scores for the following goals: biodiversity, foob provision, sense of place, and livelihoods and economies. The scores are displayed as a map (left) and histogram (right).

### Summary of average annual change in scores

##### Figure D. Average annual change in OHI Index scores.

Map of the slope estimates from a linear regression model of the Index scores from 2012 to 2016 for each region.

##### Figure E. Average annual change in OHI goal scores.

Map of the slope estimates from a linear regression model of the 10 goal scores from 2012 to 2016 for each region.

##### Figure F. Average annual change in OHI subgoal scores.

Map of the slope estimates from a linear regression model of the 10 subgoal scores from 2012 to 2016 for each region. The subgoal scores for the following goals: biodiversity, foob provision, sense of place, and livelihoods and economies. The scores are displayed as a map (left) and histogram (right).

##### Figure G. Barplot of average annual change in goal trends for each region.

The values are the slope estimate from a linear regression model of the scores from 2012 to 2016 for each region and goal.

### Evaluating the OHI model

##### Table C. Statistics used to evaluate OHI model performance for each goal/subgoal

Results (p-values) from linear regression models to determine how well various model components predict the *observed change in status*. Model 1 describes how well the overall model performs by comparing the observed change in status (2016 minus 2012 status scores) to the predicted change (2012 likely future status minus 2012 status). Statistically significant values here are ideal. Model 2 describes how well trend and “resilience minus pressure” (which reflects how the resilience and pressure components are incorported into the calculation of the likely future status score) predict the observed change in status. Model 3 describes how well trend, pressure, and resilience components predict the observed change in status. Significance in these components indicates certain aspects of the model are useful, even if the full model does not perform well. Green values indicate statistical significance (p < 0.05) in the predicted direction, and red values indicate a significant trend in the wrong direction. We were unable to evaluate (\*) species condition (subgoal of biodiversity), carbon storage, and livelihoods and economies because data sources were not updated. Results for (\*\*) habitat (subgoal of biodiversity) ansd coastal protection should be viewed skeptically because only a small portion of the data for these goals/subgoals could be updated.

##### Figure H. Evaluating OHI model performance for each goal

Relationship between different aspects of OHI scores for each goal. (Left) OHI scores in 2012 versus 2016, showing past scores predict future scores; (Middle) ‘likely future status’ in 2012 (i.e., predicted status in 2016) versus observed status in 2016; and (Right) expected change in status (OHI status minus ‘likely future status’ from 2012 scenario) and the observed change (status in 2016 minus status in 2012). Red lines indicate a one to one fit, and black lines indicate fit from a linear model.

**Artisanal opportunities**

**Species condition (biodiversity)**

**Habitat (biodiversity)**

**Coastal protection**

**Carbon storage**

**Clean waters**

**Fisheries (food provision)**

**Mariculture (food provision)**

**Iconic species (sense of place)**

**Lasting special places (sense of place)**

**Natural products**

**Tourism and recreation**

### Comparison of scores vs. ranks

##### Figure I. Change in score vs. rank for the 10 OHI goals

Change is determined by subtracting the 2012 data (ranks or scores) from the 2016 data. The changes in rank and scores are strongly correlated, but in some instances, small (probably non-significant) changes in scores can lead to large changes in rank.

##### Figure J. Change in score vs. rank for OHI subgoals

Data for subgoals of biodiversity, food provision, sense of place, and livelihoods and economies. Change is determined by subtracting the 2012 data (ranks or scores) from the 2016 data. The changes in rank and scores are strongly correlated, but in some instances, small (probably non-significant) changes in scores can lead to large changes in rank.

### Relationship between goal scores

##### Figure K. Pairwise comparison of goal scores.

Two-letter codes in the diagonal are goal abbreviations (FP = food provision, AO = artisanal fishing opportunity, NP = natural products, CS = carbon storage, CP = coastal protection, LE = coastal livelihoods and economies, TR = tourism and recreation, SP = sense of place, CW = clean water, and BD = biodiversity). Values in the upper right are correlation coefficients for each comparison, with larger font sizes indicating larger coefficients. Plots in lower left are scatterplots of the data with locally-weighted polynomial regression (LOWESS) fits shown in red.

### Relationship between OHI Index scores and social variables

We explored several models to determine whether there was a relationship between OHI scores and the Human Development Index, Cumulative Human Impacts to marine systems, and coastal population (ln population, 10 miles from shoreline).

OHI scores tended to be higher for regions with higher Human Development Index scores and lower for regions with more marine pressures (i.e., cumulative human impacts).

##### Table D. Relationship between Index scores and social variables

Model comparison to determine the relationship between OHI Index scores and the Human Development Index (HDI), Cumulative Human Impacts (CHI), and coastal population. The model with the lowest AIC score is best supported. All the best supported models included the HDI.

| Model | df | AIC | R2 (adj) |
| --- | --- | --- | --- |
| CHI | 3 | 1027 | 0.05 |
| HDI | 3 | 989 | 0.27 |
| ln(pop) | 3 | 1035 | <0.01 |
| CHI + HDI | 4 | 978 | 0.33 |
| ln(pop) + HDI | 4 | 990 | 0.27 |
| ln(pop) + CHI | 4 | 1029 | 0.041 |
| ln(pop) + CHI + HDI | 5 | 980 | 0.32 |

##### Figure L. Relationship between OHI Index and social variables

Higher OHI Index scores are associated with higher Human Development Index scores (HDI, the three best performing models included this variable). Lower OHI Index scores are associated with higher cumulative human impacts (CHI) and coastal populations, although the best model only included CHI. This relationship is difficult to resolve given there is a positive relationship between CHI and coastal population.
