# Supplementary material for: Drivers and implications of change in global ocean health over the past five years
Source: PLoS One. 2017 Jul 5;12(7):e0178267. doi: 10.1371/journal.pone.0178267 (PMC5497940; doi:10.1371/journal.pone.0178267)
Supplement: S2 File — Description of OHI philosophy and methods. (HTML) [file pone.0178267.s002.html]

S2 Methods


OHI Science | Citation policy


Code 

- Show All Code
- Hide All Code

# S2 Methods

Compiled on Thu May 11 10:00:00 2017.

# 1 The Ocean Health Index

We measure ocean health as the sustainable delivery of ten widely-held public goals for ocean ecosystems (Table 1.1). These goals represent the full suite of benefits that people want and need from the ocean, including the traditional ‘goods and services’ people often consider (e.g., fish to eat, coastal protection from nearshore habitats) as well as benefits less commonly accounted for, such as cultural values or biodiversity. The Index recognizes that people are part of coastal and ocean systems and thus values both conservation and extractive use.

**Table 1.1. The 10 goals of the Ocean Health Index**

| Goal | Description |
| --- | --- |
| Artisanal Fishing Opportunity | The opportunity for small-scale fishers to supply catch for their families, members of their local communities, or sell in local markets |
| Biodiversity | The conservation status of native marine species and key habitats that serve as a proxy for the suite of species that depend upon them |
| Carbon Storage | The condition of coastal habitats that store and sequester atmospheric carbon |
| Clean Waters | The degree to which ocean regions are free of contaminants such as chemicals, eutrophication, harmful algal blooms, disease pathogens, and trash |
| Coastal Livelihoods and Economies | Coastal and ocean-dependent livelihoods (job quantity and quality) and economies (revenues) produced by marine sectors |
| Coastal Protection | The amount of protection provided by marine and coastal habitats serving as natural buffers against incoming waves |
| Food Provision | The sustainable harvest of seafood from wild-caught fisheries and mariculture |
| Natural Products | The natural resources that are sustainably extracted from living marine resources |
| Sense of Place | The conservation status of iconic species (e.g., salmon, whales) and geographic locations that contribute to cultural identity |
| Tourism and Recreation | The value people have for experiencing and enjoying coastal areas through activities such as sailing, recreational fishing, beach-going, and bird watching |

The global Ocean Health Index has been assessed every year since 2012. The primary goal of each yearly assessment is to calculate a new year of scores using the most recent data released by the data sources. Often, in addition to incorporating an additional year of data, we make improvements to models or data sources. To ensure that scores for all years are calculated using the same methods and data sources, and can thus be compared, we recalculate scores for previous scenario years during each assessment. For the 2016 assessment, for example, in addition to calculating new 2016 scores, we include recalculated scores from 2012 to 2015. Consequently, comparisons among years should always be performed using data from the same assessment year so trends in scores reflect changes to ocean health rather than changes to methods.

Not all data layers are reported through the most current assessment year, and consequently, the OHI scores are calculated using the most recent year of available data. Details on which years are used for each data layer are provided in Table 6.2.

The Index is designed to be flexible to accommodate different scales and geographies of interest and different and new types of data (Lowndes *et al.* 2015, http://ohi-science.org). For more information about the philosophy of the Ocean Health Index and model development see Halpern et al. (2012, 2015) and http://ohi-science.org/ohi-global/, which includes information about downloading global ocean health data.

# 2 Data inclusion and data gaps

Ideally, regional and local assessments should use the best available data, but this decision limits the ability to compare across scales. For direct comparisons among locations to be valid, they must use consistent data. For this reason, we focused on using global datasets so differences in Index scores across regions would be driven by differences in ocean health rather than variation in the data. Although, in reality, many global datasets are compilations of local or regional datasets and their quality varies spatially. In some cases data for a particular component or dimension of a goal were available for most but not all countries. Gaps in these data were known to not be true zero values. Rather than exclude these data layers, we employed several different methods to fill these data gaps (Frazier *et al.* 2016).

These guidelines both motivated and constrained our methods. The development of the model frameworks for each goal (including reference points) was heavily dictated by the availability of global datasets. And, ultimately, several key elements related to ocean health could not be included due to lack of existing or appropriate global datasets. As new and better data become available in the future, details of how goals or dimensions are modeled will likely change, although the framework we have developed can accommodate these changes.

For Index scores to be comparable, every region must have a value for each data layer included in the analysis, unless it is known to not be relevant to a region. In other words, missing data are not acceptable (Burgass *et al.* 2017). Adhering to this criterion is critical to avoid influencing the Index score simply because of inclusion (or absence) of a particular data layer for any reporting region.

Gaps in data are common; many developing countries lack the resources to gather detailed datasets, and even developed, data-rich countries have inevitable data gaps. We use a variety of methods to estimate missing data, including: averages of closely related groups (e.g., regions sharing ecological, spatial, political attributes; taxonomic groups; etc.), spatial or temporal interpolation (e.g., raster or time-series data), and predictive models (e.g., regression analysis, machine learning, etc.). Gapfilling is a major source of uncertainty, especially for certain goals and regions. Given how common gaps in data are, clear documentation of gapfilling is a critical step of index development because it provides a measure of the reliability of index scores.

One of the ongoing goals of the Ocean Health Index (OHI) has been to improve our approach to dealing with missing data, by quantifying the potential influence of gapfilled data on index scores, and developing effective methods of tracking, quantifying, and communicating this information (Frazier *et al.* 2016).

# 3 Regions

For the global assessment, we calculate Ocean Health Index scores for 220 coastal countries and territorial regions (Table 3.1). Regions are based on Exclusive Economic Zone boundaries (EEZ, Claus *et al.* 2012). However, we aggregate some EEZ regions to the level of country (e.g., Hawaii is estimated as part of the larger U.S.). We have also modified some boundaries (Halpern *et al.* 2012, 2015b). We do not estimate OHI values for disputed or unclaimed areas.

**Table 3.1. Global regions**

| region | region ID | ISO code | type | administrative country |
| --- | --- | --- | --- | --- |
| Albania | 82 | ALB | country |  |
| Algeria | 84 | DZA | country |  |
| American Samoa | 151 | ASM | territory | United States |
| Amsterdam Island and Saint Paul Island | 92 | ATF | territory | France |
| Andaman and Nicobar | 26 | IND | territory | India |
| Angola | 200 | AGO | country |  |
| Anguilla | 118 | AIA | territory | United Kingdom |
| Antigua and Barbuda | 120 | ATG | country |  |
| Argentina | 172 | ARG | country |  |
| Aruba | 250 | AW | territory | Netherlands |
| Ascension | 85 | ASC | territory | United Kingdom |
| Australia | 16 | AUS | country |  |
| Azores | 55 | PRT | territory | Portugal |
| Bahamas | 110 | BHS | country |  |
| Bahrain | 52 | BHR | country |  |
| Bangladesh | 204 | BGD | country |  |
| Barbados | 124 | BRB | country |  |
| Bassas da India | 34 | ATF | territory | France |
| Belgium | 59 | BEL | country |  |
| Belize | 164 | BLZ | country |  |
| Benin | 99 | BEN | country |  |
| Bermuda | 108 | BMU | territory | United Kingdom |
| Bonaire | 245 | BQ | territory | Netherlands |
| Bosnia and Herzegovina | 232 | BIH | country |  |
| Bouvet Island | 105 | BVT | territory | Norway |
| Brazil | 171 | BRA | country |  |
| British Indian Ocean Territory | 38 | IOT | territory | United Kingdom |
| British Virgin Islands | 117 | VGB | territory | United Kingdom |
| Brunei | 247 | BRN | country |  |
| Bulgaria | 71 | BGR | country |  |
| Cambodia | 24 | KHM | country |  |
| Cameroon | 197 | CMR | country |  |
| Canada | 218 | CAN | country |  |
| Canary Islands | 58 | ESP | territory | Spain |
| Cape Verde | 56 | CPV | country |  |
| Cayman Islands | 113 | CYM | territory | United Kingdom |
| Chile | 224 | CHL | country |  |
| China | 209 | CHN | country |  |
| Christmas Island | 2 | CXR | territory | Australia |
| Clipperton Island | 107 | CPT | territory | France |
| Cocos Islands | 1 | CCK | territory | Australia |
| Colombia | 132 | COL | country |  |
| Comoro Islands | 28 | COM | country |  |
| Cook Islands | 153 | COK | territory | New Zealand |
| Costa Rica | 130 | CRI | country |  |
| Croatia | 187 | HRV | country |  |
| Crozet Islands | 91 | ATF | territory | France |
| Cuba | 112 | CUB | country |  |
| Curacao | 244 | CW | territory | Netherlands |
| Cyprus | 81 | CYP | country |  |
| Democratic Republic of the Congo | 199 | COD | country |  |
| Denmark | 175 | DNK | country |  |
| Djibouti | 46 | DJI | country |  |
| Dominica | 123 | DMA | country |  |
| Dominican Republic | 115 | DOM | country |  |
| East Timor | 231 | TLS | country |  |
| Ecuador | 137 | ECU | country |  |
| Egypt | 214 | EGY | country |  |
| El Salvador | 134 | SLV | country |  |
| Equatorial Guinea | 104 | GNQ | country |  |
| Eritrea | 45 | ERI | country |  |
| Estonia | 70 | EST | country |  |
| Faeroe Islands | 141 | FRO | territory | Denmark |
| Falkland Islands | 95 | FLK | territory | United Kingdom |
| Fiji | 18 | FJI | country |  |
| Finland | 174 | FIN | country |  |
| France | 179 | FRA | country |  |
| French Guiana | 169 | GUF | territory | France |
| French Polynesia | 147 | PYF | territory | France |
| Gabon | 198 | GAB | country |  |
| Gambia | 65 | GMB | country |  |
| Georgia | 74 | GEO | country |  |
| Germany | 176 | DEU | country |  |
| Ghana | 106 | GHA | country |  |
| Gibraltar | 60 | GIB | territory | United Kingdom |
| Glorioso Islands | 30 | ATF | territory | France |
| Greece | 80 | GRC | country |  |
| Greenland | 145 | GRL | territory | Denmark |
| Grenada | 125 | GRD | country |  |
| Guadeloupe and Martinique | 140 | GP-MQ | territory | France |
| Guatemala | 136 | GTM | country |  |
| Guernsey | 228 | GGY | territory | United Kingdom |
| Guinea | 194 | GIN | country |  |
| Guinea Bissau | 193 | GNB | country |  |
| Guyana | 167 | GUY | country |  |
| Haiti | 114 | HTI | country |  |
| Heard and McDonald Islands | 94 | HMD | territory | Australia |
| Honduras | 133 | HND | country |  |
| Howland Island and Baker Island | 158 | UMI | territory | United States |
| Iceland | 143 | ISL | country |  |
| Ile Europa | 35 | ATF | territory | France |
| Ile Tromelin | 36 | ATF | territory | France |
| India | 203 | IND | country |  |
| Indonesia | 216 | IDN | country |  |
| Iran | 191 | IRN | country |  |
| Iraq | 192 | IRQ | country |  |
| Ireland | 181 | IRL | country |  |
| Israel | 79 | ISR | country |  |
| Italy | 184 | ITA | country |  |
| Ivory Coast | 195 | CIV | country |  |
| Jamaica | 166 | JAM | country |  |
| Jan Mayen | 144 | SJM | territory | Norway |
| Japan | 210 | JPN | country |  |
| Jarvis Island | 149 | UMI | territory | United States |
| Jersey | 227 | JEY | territory | United Kingdom |
| Johnston Atoll | 159 | UMI | territory | United States |
| Jordan | 215 | JOR | country |  |
| Juan de Nova Island | 33 | ATF | territory | France |
| Kenya | 43 | KEN | country |  |
| Kerguelen Islands | 93 | ATF | territory | France |
| Kiribati | 212 | KIR | country |  |
| Kuwait | 51 | KWT | country |  |
| Latvia | 69 | LVA | country |  |
| Lebanon | 78 | LBN | country |  |
| Liberia | 97 | LBR | country |  |
| Libya | 67 | LBY | country |  |
| Line Group | 148 | KIR | territory | Kiribati |
| Lithuania | 189 | LTU | country |  |
| Macquarie Island | 4 | AUS | territory | Australia |
| Madagascar | 42 | MDG | country |  |
| Madeira | 57 | PRT | territory | Portugal |
| Malaysia | 206 | MYS | country |  |
| Maldives | 39 | MDV | country |  |
| Malta | 68 | MLT | country |  |
| Marshall Islands | 11 | MHL | country |  |
| Mauritania | 64 | MRT | country |  |
| Mauritius | 37 | MUS | country |  |
| Mayotte | 29 | MYT | territory | France |
| Mexico | 135 | MEX | country |  |
| Micronesia | 9 | FSM | country |  |
| Monaco | 185 | MCO | country |  |
| Montenegro | 186 | MNE | country |  |
| Montserrat | 121 | MSR | territory | United Kingdom |
| Morocco | 62 | MAR | country |  |
| Mozambique | 41 | MOZ | country |  |
| Myanmar | 205 | MMR | country |  |
| Namibia | 101 | NAM | country |  |
| Nauru | 10 | NRU | country |  |
| Netherlands | 177 | NLD | country |  |
| New Caledonia | 5 | NCL | territory | France |
| New Zealand | 162 | NZL | country |  |
| Nicaragua | 131 | NIC | country |  |
| Nigeria | 196 | NGA | country |  |
| Niue | 154 | NIU | territory | New Zealand |
| Norfolk Island | 3 | NFK | territory | Australia |
| Northern Mariana Islands and Guam | 13 | MNP | territory | United States |
| Northern Saint-Martin | 221 | MAF | territory | France |
| North Korea | 21 | PRK | country |  |
| Norway | 223 | NOR | country |  |
| Oecussi Ambeno | 237 | TLS | territory | East Timor |
| Oman | 48 | OMN | country |  |
| Pakistan | 53 | PAK | country |  |
| Palau | 8 | PLW | country |  |
| Palmyra Atoll | 150 | UMI | territory | United States |
| Panama | 129 | PAN | country |  |
| Papua New Guinea | 17 | PNG | country |  |
| Peru | 138 | PER | country |  |
| Philippines | 15 | PHL | country |  |
| Phoenix Group | 157 | KIR | territory | Kiribati |
| Pitcairn | 146 | PCN | territory | United Kingdom |
| Poland | 178 | POL | country |  |
| Portugal | 183 | PRT | country |  |
| Prince Edward Islands | 90 | ZAF | territory | South Africa |
| Puerto Rico and Virgin Islands of the United States | 116 | PRI | territory | United States |
| Qatar | 190 | QAT | country |  |
| Republique du Congo | 100 | COG | territory | R\_publique du Congo |
| Reunion | 32 | REU | territory | France |
| Romania | 72 | ROU | country |  |
| Russia | 73 | RUS | country |  |
| Saba | 248 | BES | territory | Netherlands |
| Saint Helena | 86 | SHN | territory | United Kingdom |
| Saint Kitts and Nevis | 119 | KNA | country |  |
| Saint Lucia | 122 | LCA | country |  |
| Saint Pierre and Miquelon | 219 | SPM | territory | France |
| Saint Vincent and the Grenadines | 127 | VCT | country |  |
| Samoa | 152 | WSM | country |  |
| Sao Tome and Principe | 103 | STP | country |  |
| Saudi Arabia | 50 | SAU | country |  |
| Senegal | 66 | SEN | country |  |
| Seychelles | 31 | SYC | country |  |
| Sierra Leone | 96 | SLE | country |  |
| Singapore | 208 | SGP | country |  |
| Sint Eustatius | 249 | ANT | territory | Netherlands |
| Sint Maarten | 220 | SXM | territory | Netherlands |
| Slovenia | 188 | SVN | country |  |
| Solomon Islands | 7 | SLB | country |  |
| Somalia | 44 | SOM | country |  |
| South Africa | 102 | ZAF | country |  |
| South Georgia and the South Sandwich Islands | 89 | SGS | territory | United Kingdom |
| South Korea | 20 | KOR | country |  |
| Spain | 182 | ESP | country |  |
| Sri Lanka | 40 | LKA | country |  |
| Sudan | 49 | SDN | country |  |
| Suriname | 168 | SUR | country |  |
| Sweden | 222 | SWE | country |  |
| Syria | 77 | SYR | country |  |
| Taiwan | 14 | TWN | country |  |
| Tanzania | 202 | TZA | country |  |
| Thailand | 25 | THA | country |  |
| Togo | 98 | TGO | country |  |
| Tokelau | 156 | TKL | territory | New Zealand |
| Tonga | 155 | TON | country |  |
| Trinidad and Tobago | 126 | TTO | country |  |
| Tristan da Cunha | 88 | TAA | territory | United Kingdom |
| Tunisia | 61 | TUN | country |  |
| Turkey | 76 | TUR | country |  |
| Turks and Caicos Islands | 111 | TCA | territory | United Kingdom |
| Tuvalu | 19 | TUV | country |  |
| Ukraine | 75 | UKR | country |  |
| United Arab Emirates | 54 | ARE | country |  |
| United Kingdom | 180 | GBR | country |  |
| United States | 163 | USA | country |  |
| Uruguay | 173 | URY | country |  |
| Vanuatu | 6 | VUT | country |  |
| Venezuela | 139 | VEN | country |  |
| Vietnam | 207 | VNM | country |  |
| Wake Island | 12 | UMI | territory | United States |
| Wallis and Futuna | 161 | WLF | territory | France |
| Western Sahara | 63 | ESH | territory | Morocco |
| Yemen | 47 | YEM | country |  |

# 4 Models

## 4.1 Regional and global Index scores

The overall index score for each region (\(I\_{region}\)) is calculated as a weighted average of all the scores (\(G\)), for each goal (\(g\)) such that:

\[
{ I\_{region} }\quad =\quad \frac { \displaystyle\sum\_{ g=1 }^{ N }{ { w }\_{ g }{ G }\_{ g } } }{ \displaystyle\sum \_{ g=1 }^{ N }{ { w }\_{ g } } }, (Eq. 4.1)
\]

where, \(w\_{g}\) is the weight for each goal.

For the global assessment, the goal weights (\(w\_{g}\)) were assumed to be equal, even though we know this assumption does not hold for most individuals or across individuals within communities. Ideally these weights would be derived empirically, but such an effort would require surveying a full spectrum of people from every single country. This was beyond the scope of this project, but may be possible in a future application of the Index.

In many places certain goals are not relevant, for example, production-focused goals typically do not apply to uninhabited islands, and the coastal protection or carbon storage goals will not apply to regions without the relevant coastal ecosystems.

The overall global index score (\(I\_{global}\)) is calculated as the area weighted average of the index scores (\(I\_{region}\)) for each region (\(i\)):

\[
{ I }\_{ global }\quad =\quad \frac { \displaystyle\sum \_{ i=1 }^{ N }{ { a }\_{ i }{ I }\_{ region,i } } }{ \displaystyle\sum \_{ i=1 }^{ N }{ { a }\_{ i } } }, (Eq. 4.2)
\]

where, \(a\_{i}\) is each region’s ocean area, based on the EEZ area.

## 4.2 Goal scores

Each goal score is the average of its current status and likely future status (Figure 4.1). The Index assesses the current status of each goal relative to a reference point. Likely future status is estimated using: recent trends in current status; pressures that can stress the system and threaten future delivery of benefits; and resilience to such pressures, due to governance, institutional and ecological factors.

**Figure 4.1. Pie chart describing the contribution of each dimension to the goal score**

Each goal score, \(G\), is the average of its present status, \(x\), and its likely near-term future status, \(\hat x\_{F}\):

\[
G \quad =\quad \frac {x \quad +\quad \hat x\_{F} }{ 2 }, (Eq. 4.3)
\]

The present status of goal, \(x\), is its present state, \(X\), relative to a reference point, \(X\_{R}\), uniquely chosen for each goal:

\[
{ x }\_{ i }\quad =\quad \frac { X }{ X\_{R} }, (Eq. 4.4)
\]

The reference point, \(X\_{R}\), can be determined mechanistically using a production function (e.g., maximum sustainable yield, MSY, for fisheries), spatially by means of comparison with another region (e.g., country X represents the best possible known case), temporally using a past benchmark (e.g., historical habitat extent), or in some cases via known (e.g., zero pollution) or established (e.g., 30% of waters set aside in MPAs) targets. Past benchmarks can either be a fixed point in time or a moving target (e.g., five years prior to most current data). The type of reference point can have important implications for interpretations of how a goal is doing in any given country.

For each region, the estimate of a goal’s likely near-term future status is a function of its present status, \(x\) modified by: recent trends, \(T\), in status; current cumulative pressures, \(p\), acting on the goal; and social and ecological resilience, \(r\), to pressures given the governance and social institutions in place to protect or regulate the system and the ecological condition of the system:

\[
\hat {x} \_{F} \quad = \quad \left[ 1 \quad + \quad \beta T \quad + \quad \left( 1\quad -\quad \beta \right) \left( r \quad - \quad p \right) \right] x, (Eq. 4.5)
\]

where, \(\beta\) represents the relative importance of the trend versus the resilience and pressure terms in determining the likely trajectory of the goal status into the future. We assume \(\beta = 0.67\), which makes trend twice as important as the pressure/resilience component. We chose this value because we believe the direct measure of trend is a better indicator of future (i.e., in five years) condition than indirect measures of pressure and resilience.

The role of the resilience and pressure dimensions is to improve our understanding of the likely near-term future condition by incorporating additional information beyond that provided by the recent trend. Pressure or resilience measures that were in existence in the past may have a cumulative effect that has not yet manifested itself in trend (e.g., fishing pressure may have increasingly negative impacts as successive year classes of fish become increasingly less abundant; resilience due to establishment of a marine protected area (MPA) may require a number of years before its benefits become apparent). In addition, the recent trend does not capture the effect of current levels of resilience and pressures. The expectation of a likely future condition suggested by the trend will become more or less optimistic depending on the resilience and pressure dimensions. If the effects are equal they cancel each other out.

Both resilience and pressure dimensions are scaled from 0 to 1, and trend is constrained to -1.0 ≤ \(T\) ≤ 1.0 (i.e., values outside this range are clamped to range end values).

The likely future status cannot exceed the maximum possible value of the status for each goal, which is 1.0. In reality data are rarely perfect, creating potential situations where likely future condition exceeds 1.0. To address these cases, we implemented two rules. First, if current status = 1.0, then trend is set = 0.0, since any trend > 0.0 in those cases must be due to incomplete or imperfect data. Second, status and likely future status scores were constrained to maximum value of 1.

## 4.3 Likely future status dimensions

Three dimensions are used to calculate likely future status: trends, pressure, and resilience. This section describes the calculations underlying these three dimensions.

### 4.3.1 Trend

Trend is the proportional change in status predicted to occur in 5 years, based on recent status data. In most cases, this is calculated by estimating the yearly change in status using a linear regression model (i.e., slope estimate) of the five most recent years of status data and multiplying this value by 5 to estimate the change five years into the future. To determine proportional change, we divide the slope estimate by the status value of the earliest year of data used in the trend calculation.

In OHI assessments prior to 2016, we calculated trend as the *absolute* change in status predicted to occur in 5 years. In 2016, we began calculating the *proportional* change by dividing the slope estimate by the status of the earliest year used in the trend calculation. Although this change rarely had a large effect on trend, or ultimate score values, this method is more consistent with how trend data is incorporated into the likely future status model (Eq. 4.5). If the \(\beta\), pressure (\(p\)), and resilience (\(r\)) components of the likely future status model are ignored (this assumes the pressure and resilience components fully cancel each other out), the equation becomes:

\(x(1 + trend)\),

where, \(x\) is the current status. Given this, if \(x=50\), and we expect trend to increase by 10% over 5 years, then likely future status would be: \(50(1 + 0.10) = 55\).

Trends indicate proportinal change in status, so they typically range from -100% to +100% (or, -1.0 to +1.0), therefore we constrained values to this range.

For all goals we included the trend estimate, even if the linear model was not statistically significant (i.e., P<0.05). We chose to include these values for two key reasons: 1) we were not trying to predict the future but instead only indicate likely condition. 2) in nearly all cases we did not have sufficient data to conduct more rigorous trend analyses.

In some cases, we were not able to estimate trend using status data due to data limitations. In these cases, we used alternative methods to estimate trend. For example, for the species condition subgoal, we converted IUCN trend information (e.g., “increasing”, “decreasing”, etc.) to numeric values and averaged these for each region. Specific details about trend calculations for each goal are provided in section 5.

We recognize several possible shortcomings in using past trends to estimate likely future status. We assume a simple linear trend, but this is not always the case due to a variety of variables such as altered pressures and resilience responses, nonlinear patterns in system response, stochastic environmental and biological variability, and simple bounding conditions (status cannot go below zero or above 1.0, and so the trend must level off as it approaches these values). Also, it is important to note that the same trend value could reflect many different processes. For example, declines due to unsustainable harvest of a resource can look identical to declines due to restrictions placed on resource users to allow the resource to recover. It also may be too short a time frame to determine true trends or the causes of those trends, but the intent here is more about informing the likely near-term trajectory.

### 4.3.2 Pressure

The pressure score, \(p\), describes the cumulative pressures acting on a goal which suppress the goal score. Pressure scores range from 0 to 1, and they are calculated for each goal and region and include both ecological (\(p\_{E}\)) and social pressures (\(p\_{S}\)) (Table 4.1, Figure 4.2), such that:

\[
{ p }\quad =\quad \gamma \*{ p }\_{ E }\quad +\quad (1-\gamma )\*{ p }\_{ S }, (Eq. 4.6)
\]

where \(\gamma\) is the relative weight for ecological vs. social pressures and equals 0.5 for the global assessment. At global scales, little evidence exists to support unequal weighting of ecological and social pressures for most goals; furthermore, unequal weighting would require unique values for each goal and there is currently no empirical work to guide such decisions. At local or regional scales there may be clear evidence for unequal weights per goal and \(\gamma\) should be adjusted accordingly.

**Figure 4.2. Pressure components** Pressure is calculated using both social and ecological pressures. Ecological pressures include 5 subcategories (fishing pressure, habitat destruction, climate change, water pollution, and species/genetic introductions).

**Table 4.1. Pressure data and categories** Description of the stressor data layers used to calculate overall pressure for each goal and region for the global assessment (descriptions of pressure data in section 6). Each data layer is assigned to an ecological or social category, and ecological data are assigned to one of five subcategories.

| Data | Short name | Category | Subcategory | Description |
| --- | --- | --- | --- | --- |
| Chemical pollution | po\_chemicals | ecological | pollution | Modeled chemical pollution within EEZ from commercial shipping traffic, ports and harbors, land-based pesticide use (organic pollution), and urban runoff (inorganic pollution) |
| Coastal chemical pollution | po\_chemicals\_3nm | ecological | pollution | Modeled chemical pollution within 3nm of coastline from commercial shipping traffic, ports and harbors, land-based pesticide use (organic pollution), and urban runoff (inorganic pollution) |
| Pathogen pollution | po\_pathogens | ecological | pollution | Percent of population without access to improved sanitation facilities as a proxy for pathogen pollution |
| Nutrient pollution | po\_nutrients | ecological | pollution | Modeled nutrient pollution within 3nm of coastline based on fertilizer consumption |
| Coastal nutrient pollution | po\_nutrients\_3nm | ecological | pollution | Modeled nutrient pollution within EEZ based on fertilizer consumption |
| Marine plastics | po\_trash | ecological | pollution | Global marine plastic pollution |
| Nonindigenous species | sp\_alien | ecological | alien species | Measure of harmful invasive species |
| Genetic escapes | sp\_genetic | ecological | alien species | Introduced mariculture species (Mariculture Sustainability Index) as a proxy for genetic escapes |
| Subtidal soft bottom habitat destruction | hd\_subtidal\_sb | ecological | habitat destruction | Demersal destructive commercial fishing practices (i.e., trawling) in soft bottom habitat as a proxy for soft bottom habitat destruction |
| Subtidal hardbottom habitat destruction | hd\_subtidal\_hb | ecological | habitat destruction | High bycatch artisanal fishing practices (blast fishing) as a proxy for subtidal hard bottom habitat destruction |
| Intertidal habitat destruction | hd\_intertidal | ecological | habitat destruction | Coastal population density (25 mi from shore) as a proxy for intertidal habitat destruction |
| High bycatch due to commercial fishing | fp\_com\_hb | ecological | fishing pressure | Modeled destructive commercial fishing practices by 5 gear types and scaled by Net Primary Productivity |
| Low bycatch due to commercial fishing | fp\_com\_lb | ecological | fishing pressure | Modeled destructive commercial fishing practices by 2 gear types and scaled by Net Primary Productivity |
| Low bycatch due to artisanal fishing | fp\_art\_lb | ecological | fishing pressure | Extent of artisanal fishing (including: artisanal, subsistence, and recreational catch) |
| High bycatch due to artisanal fishing | fp\_art\_hb | ecological | fishing pressure | The presence of destructive artisanal blast and poison (cyanide) fishing. |
| Targeted harvest of cetaceans and marine turtles | fp\_targetharvest | ecological | fishing pressure | Targeted harvest of cetaceans and marine turtles |
| Sea surface temperature | cc\_sst | ecological | climate change | Sea surface temperature anomalies |
| Ocean acidification | cc\_acid | ecological | climate change | Ocean acidification pressure scaled using biological thresholds |
| UV radiation | cc\_uv | ecological | climate change | Modeled UV radiation |
| Sea level rise | cc\_slr | ecological | climate change | Sea level rise pressure |
| Weakness of governance | ss\_wgi | social | social | Inverse of World Governance Indicators (WGI) six combined scores |
| Weakness of social progress | ss\_spi | social | social | Inverse of Social Progress Index scores |

#### 4.3.2.1 Ecological pressure

We assessed five broad, globally-relevant categories of ecological stressors: fishing pressure, habitat destruction, climate change (including ocean acidification), water pollution, and species introductions (invasive species and genetic escapes). The five categories are intended to capture known pressures to the social-ecological system associated with each goal. Each pressure category may include several stressors. The intensity of each stressor within each OHI region is scaled from 0 to 1, with 1 indicating the highest stress (e.g., example of one of these data layers is sea surface temperature).

We determined the rank sensitivity of each goal/subgoal to each stressor (or, when possible, an element of the goal, such as a specific habitat). We ranked ecological pressures as having ‘high’ (score = 3), ‘medium’ (score = 2), ‘low’ (score = 1), or ‘no’ (score = NA) impact (Table 4.2). Wherever possible we relied on peer-reviewed literature to establish these rankings, and relied on our collective expert judgment in cases with no available literature (Table S28 in Halpern et al. 2012). The pressure ranks are based on a rough estimate of the global average intensity and frequency of the stressor. We recognize that this will create over- and under-estimates for different places around the planet, but to address such variance in a meaningful way would require a separate weighting matrix for every single region on the planet, which is not feasible at this time.

**Table 4.2. Pressure matrix** Rank sensitivity of each goal (or, goal element) to each stressor.

| goal | element | cc\_acid | cc\_slr | cc\_sst | cc\_uv | fp\_art\_hb | fp\_art\_lb | fp\_com\_hb | fp\_com\_lb | fp\_targetharvest | hd\_intertidal | hd\_subtidal\_hb | hd\_subtidal\_sb | po\_chemicals | po\_chemicals\_3nm | po\_nutrients | po\_nutrients\_3nm | po\_pathogens | po\_trash | sp\_alien | sp\_genetic | ss\_spi | ss\_wgi |
| --- | --- | --- | --- | --- | --- | --- | --- | --- | --- | --- | --- | --- | --- | --- | --- | --- | --- | --- | --- | --- | --- | --- | --- |
| AO |  |  |  |  |  | 3 |  | 2 | 1 |  | 1 | 3 | 1 |  | 1 |  | 1 |  |  | 1 |  | 1 | 1 |
| CP | coral | 1 | 2 | 3 | 1 |  |  |  |  |  |  | 3 |  |  | 1 |  | 2 |  |  | 1 |  | 1 | 1 |
| CP | mangrove |  | 2 |  |  |  |  |  |  |  | 3 |  |  |  | 1 |  | 1 |  |  |  |  | 1 | 1 |
| CP | saltmarsh |  | 3 |  |  |  |  |  |  |  | 3 |  |  |  | 1 |  | 2 |  |  | 1 |  | 1 | 1 |
| CP | seagrass | 1 | 3 | 2 |  |  |  |  |  |  | 3 |  |  |  | 2 |  | 3 |  |  | 1 |  | 1 | 1 |
| CP | seaice shoreline |  | 2 | 3 |  |  |  |  |  |  |  |  |  |  |  |  |  |  |  |  |  | 1 | 1 |
| CS | mangrove |  | 1 |  |  |  |  |  |  |  | 3 |  |  |  | 1 |  | 1 |  |  |  |  | 1 | 1 |
| CS | saltmarsh |  | 2 |  |  |  |  |  |  |  | 3 |  |  |  | 1 |  | 2 |  |  | 1 |  | 1 | 1 |
| CS | seagrass | 1 | 2 | 2 |  |  |  |  |  |  | 3 |  |  |  | 2 |  | 3 |  |  | 1 |  | 1 | 1 |
| CW |  |  |  |  |  |  |  |  |  |  |  |  |  |  | 3 |  | 3 | 3 | 3 |  |  | 1 | 1 |
| ECO | Aquarium Trade Fishing | 1 |  | 1 |  | 3 | 1 |  |  |  |  | 3 |  | 2 |  | 1 |  |  |  | 1 |  | 1 | 1 |
| ECO | Commercial Fishing |  |  |  |  | 2 | 1 | 3 | 1 |  | 1 | 2 | 2 | 2 |  | 1 |  |  |  | 1 | 1 | 1 | 1 |
| ECO | Mariculture |  | 1 |  |  |  |  |  |  |  |  |  |  | 2 |  |  | 3 |  |  |  |  | 1 | 1 |
| ECO | Marine Mammal Watching |  |  |  |  |  |  | 1 |  |  |  |  |  |  |  |  |  |  | 1 |  |  | 1 | 1 |
| ECO | Tourism |  | 2 |  |  |  |  |  |  |  |  |  |  | 3 |  |  | 3 | 3 | 3 |  |  | 1 | 1 |
| ECO | Wave & Tidal Energy |  | 1 |  |  |  |  |  |  |  |  |  |  |  |  |  |  |  |  |  |  | 1 | 1 |
| FIS |  |  |  |  |  | 2 | 1 | 3 | 1 |  | 1 | 2 | 2 | 1 |  | 1 |  |  |  | 1 | 1 | 1 | 1 |
| HAB | coral | 1 | 1 | 3 | 1 | 3 |  |  |  |  |  | 3 |  |  | 1 |  | 2 |  |  | 1 |  | 1 | 1 |
| HAB | mangrove |  | 1 |  |  |  |  |  |  |  | 3 |  |  |  | 1 |  | 1 |  |  |  |  | 1 | 1 |
| HAB | saltmarsh |  | 2 |  |  |  |  |  |  |  | 3 |  |  |  | 1 |  | 2 |  |  | 1 |  | 1 | 1 |
| HAB | seagrass | 1 | 2 | 2 |  |  |  |  |  |  | 3 |  |  |  | 2 |  | 3 |  |  | 1 |  | 1 | 1 |
| HAB | seaice edge |  | 1 | 3 |  |  |  |  |  |  |  |  |  |  |  |  |  |  |  |  |  | 1 | 1 |
| HAB | soft bottom |  |  |  |  |  | 1 | 3 | 1 |  |  |  | 3 |  | 2 |  | 2 |  |  | 1 |  | 1 | 1 |
| ICO |  | 1 |  | 1 |  | 2 |  | 2 |  | 2 | 3 | 2 |  |  | 3 |  | 1 |  | 1 | 1 |  | 1 | 1 |
| LIV | Commercial Fishing |  |  |  |  | 2 | 1 | 3 | 1 |  | 1 | 2 | 2 | 2 |  | 1 |  |  |  | 1 | 1 | 1 | 1 |
| LIV | Mariculture |  | 1 |  |  |  |  |  |  |  |  |  |  | 2 |  |  | 3 |  |  |  |  | 1 | 1 |
| LIV | Marine Mammal Watching |  |  |  |  |  |  | 1 |  |  |  |  |  |  |  |  |  |  | 1 |  |  | 1 | 1 |
| LIV | Ports & Harbors |  | 2 |  |  |  |  |  |  |  |  |  |  |  |  |  |  |  |  | 1 |  | 1 | 1 |
| LIV | Ship & Boat Building |  |  |  |  |  |  |  |  |  |  |  |  |  |  |  |  |  |  |  |  | 1 | 1 |
| LIV | Tourism |  | 2 |  |  |  |  |  |  |  |  |  |  | 3 |  |  | 3 | 3 | 3 |  |  | 1 | 1 |
| LIV | Transportation & Shipping |  |  |  |  |  |  |  |  |  |  |  |  |  |  |  |  |  |  | 1 |  | 1 | 1 |
| LIV | Wave & Tidal Energy |  | 1 |  |  |  |  |  |  |  |  |  |  |  |  |  |  |  |  |  |  | 1 | 1 |
| LSP |  |  | 1 |  |  |  |  |  |  |  | 3 | 2 |  |  | 2 |  | 2 |  | 3 | 1 |  | 1 | 1 |
| MAR |  |  | 1 |  |  |  |  |  |  |  |  |  |  | 2 |  |  | 3 |  |  |  |  | 1 | 1 |
| NP | corals | 1 | 1 | 3 | 1 |  |  |  |  |  |  | 3 |  | 1 |  | 2 |  |  |  | 1 |  | 1 | 1 |
| NP | fish oil |  |  | 1 |  |  |  |  | 2 |  |  |  | 2 | 2 |  | 1 |  |  |  | 1 |  | 1 | 1 |
| NP | ornamentals |  |  | 1 |  | 3 | 1 |  |  |  |  | 3 |  | 2 |  | 1 |  |  |  | 1 |  | 1 | 1 |
| NP | seaweeds |  |  | 1 |  |  |  |  |  |  | 1 |  |  | 2 |  | 2 |  |  |  | 1 |  | 1 | 1 |
| NP | shells | 1 |  | 1 |  |  |  |  |  |  | 1 |  | 2 |  |  | 1 |  |  |  | 1 |  | 1 | 1 |
| NP | sponges | 1 |  | 1 |  | 1 |  |  |  |  |  |  | 3 |  |  | 1 |  |  |  | 1 |  | 1 | 1 |
| SPP |  | 1 |  | 1 | 1 | 2 | 1 | 3 | 1 | 1 | 2 | 2 | 3 | 2 |  | 3 |  |  | 1 | 1 | 1 | 1 | 1 |
| TR |  |  | 2 |  |  |  |  |  |  |  |  |  |  |  | 3 |  | 3 | 3 | 3 |  |  | 1 | 1 |

To estimate the cumulative effect of the ecological pressures, \(P\_E\), we first determined the cumulative pressure, \(p\), within each ecological category, \(i\) (e.g., pollution, fishing, etc.):

\[
{ p }\_{ i }\quad =\quad \frac { \displaystyle\sum \_{ i=1 }^{ N }{ { w }\_{ i }{ s }\_{ i } } }{ 3 }, (Eq. 4.7)
\]

Where \(w\_i\) is the sensitivity ranks (Table 4.2) describing the relative sensitivity of each goal to each stressor, and \(s\_i\) is intensity of the stressor in each region on a scale of 0-1. We divided by the maximum weighted intensity that could be achieved by the worst stressor (max = 3.0).

If \(p\_i\) > 1.0, we set the value equal to 1.0. This formulation assumes that any cumulative pressure load greater than the maximum intensity of the worst stressor is equivalent to maximum stressor intensity.

For the goals for which sensitivity ranks were assigned for specific habitats or livelihood sectors (i.e., goal elements), we calculated the weighted sum of the pressures for only those habitats or sectors that were present in the country.

The overall ecological pressure, \(p\_E\), acting on each goal and region was calculated as the weighted-average of the pressure scores, \(p\), for each category, \(i\), with weights set as the maximum rank in each pressure category (\(w\_{i\\_max}\)) for each goal, such that:

\[
{ p }\_{ E }\quad =\quad \frac { \displaystyle\sum \_{ i=1 }^{ N }{ { (w }\_{ i\\_ max }\*{ p }\_{ i }) } }{ \displaystyle\sum\_{ i=1 }^{ N } { { w }\_{ i\\_ max } } }, (Eq. 4.8)
\]

Stressors that have no impact drop out rather than being assigned a rank of zero, which would affect the average score.

#### 4.3.2.2 Social pressures

Social pressures describe the lack of effectiveness of government and social institutions. Social stressors are described for each region on a scale of 0 to 1 (with one indicating the highest pressure). Social pressure is then calculated as the average of the social stressors:

\[
{ p }\_{ S }\quad =\quad \frac { \displaystyle\sum \_{ i=1 }^{ N }{ z\_{ i } } }{ N }, (Eq. 4.9)
\]

where \(z\_{i}\) are the social pressure measures specific to the goal. Unequal weighting may be appropriate in some cases but is difficult to assess currently, particularly at the global scale.

#### 4.3.2.3 Caveats

There were a number of ecological pressures not included in our assessment, including altered sediment regimes, noise and light pollution, toxic chemicals from point sources, nutrient pollution from atmospheric deposition and land-based sources other than fertilizer application to agricultural land. In all cases, global data do not exist in a format that would allow for adequate comparisons within and among countries. Future global or regional iterations of the Index could include these data as they become available.

The calculation of ecological pressures is sensitive to the number of stressors within each category (but not to the number of categories). Inclusion of additional stressors within categories would require careful calibration of ranks so that the cumulative effect of a larger number of stressors does not overestimate pressure.

A key assumption in our assessment of ecological pressures is that each goal has a linear and additive response to increases in intensity of the stressors. Clearly many ecosystems respond non-linearly to increased stressor intensity, exhibiting threshold responses, and there are likely nonlinear interactions among stressors. Unfortunately little is known about the nature of these types of nonlinearities and interactions so we could not include them in any meaningful way.

### 4.3.3 Resilience

To calculate resilience for each goal and region, \(r\), we assess three resilience categories (Table 4.3, Figure 4.3): ecological integrity, \(Y\_{E}\), regulatory efforts that address ecological pressures, \(Y\_R\), and social integrity, \(Y\_{S}\). The first two measures address ecological resilience while the third addresses social resilience. When all three aspects are relevant to a goal, resilience is calculated as:

\[
r\quad =\quad \gamma \*(\frac { { Y }\_{ E }+ {Y}\_{R} }{ 2 } )+(1-\gamma )\*{ Y }\_{ S }, (Eq. 4.10)
\]

We chose \(\gamma = 0.5\) so the weight of resilience components that address ecological systems (ecosystem and regulatory) vs. social systems would be equivalent to the proportions used in the model to calculate pressure.

**Figure 4.3. Resilience components** Resilience includes both ecological and social resilience categories. Ecological resilience includes an ecosystem and regulatory category. The regulatory category includes 5 subcategories that mirror the pressure categories (fishing pressure, habitat destruction, climate change, water pollution, and species/genetic introductions) as well as a goal-specific category.

Each resilience category is composed of 1 or more data layers (Table 4.3) with values scaled from 0-1, reflecting the magnitude of resilience, for each region (an example of one of these data layers describes tourism regulations that preserve biodiversity). Each resilience data layer is assigned a weight of 0.5 or 1 (Table 4.3) that is applied equally across all the goals (or, goal elements) influenced by the resilience layer (i.e., resilience matrix, Table 4.4). This information is used to calculate a score for each resilience category. The weight reflects information about governance.

**Table 4.3. Resilience categories and weights** The data layers used to calculate resilience for each goal and region for the global assessment (descriptions of data layers and sources are in section 6). Each data layer is assigned to an ecological or social category. The ecological category is broken into an ecosystem and regulatory category type.

| Data | Short name | Category | Category type | Subcategory | Weight |
| --- | --- | --- | --- | --- | --- |
| Measure of ecological integrity | species\_diversity\_eez | ecological | ecosystem | ecological | 1.0 |
| Measure of coastal ecological integrity | species\_diversity\_3nm | ecological | ecosystem | ecological | 1.0 |
| Management of nonindigenous species | sp\_alien\_species | ecological | regulatory | alien species | 1.0 |
| CITES signatories | g\_cites | ecological | regulatory | goal | 0.5 |
| Coastal protected marine areas (fishing preservation) | fp\_mpa\_coast | ecological | regulatory | fishing pressure | 1.0 |
| EEZ protected marine areas (fishing preservation) | fp\_mpa\_eez | ecological | regulatory | fishing pressure | 1.0 |
| Management of habitat to protect fisheries biodiversity | fp\_habitat | ecological | regulatory | fishing pressure | 1.0 |
| Commercial fishing management | fp\_mora | ecological | regulatory | fishing pressure | 1.0 |
| Artisanal fisheries management effectiveness | fp\_mora\_artisanal | ecological | regulatory | fishing pressure | 1.0 |
| Management of habitat to protect habitat biodiversity | hd\_habitat | ecological | regulatory | habitat destruction | 1.0 |
| Coastal protected marine areas (habitat preservation) | hd\_mpa\_coast | ecological | regulatory | habitat destruction | 1.0 |
| EEZ protected marine areas (habitat preservation) | hd\_mpa\_eez | ecological | regulatory | habitat destruction | 1.0 |
| Management of mariculture to preserve biodiversity | g\_mariculture | ecological | regulatory | goal | 1.0 |
| Mariculture Sustainability Index | g\_msi\_gov | ecological | regulatory | goal | 1.0 |
| Management of tourism to preserve biodiversity | g\_tourism | ecological | regulatory | goal | 1.0 |
| Management of waters to preserve biodiversity | po\_water | ecological | regulatory | pollution | 1.0 |
| Global Competitiveness Index (GCI) scores | li\_gci | social | social | social | 1.0 |
| Economic diversity | li\_sector\_evenness | social | social | social | 1.0 |
| Strength of governance | wgi\_all | social | social | social | 1.0 |
| Social Progress Index | res\_spi | social | social | social | 1.0 |

**Table 4.4. Resilience matrix** Describes which goals/subgoals (and goal elements) are influenced by the resilience data layers.

| goal | element | po\_water | hd\_mpa\_coast | hd\_mpa\_eez | hd\_habitat | sp\_alien\_species | fp\_mpa\_coast | fp\_mpa\_eez | fp\_habitat | fp\_mora | fp\_mora\_artisanal | g\_tourism | g\_mariculture | g\_msi\_gov | g\_cites | species\_diversity\_3nm | species\_diversity\_eez | wgi\_all | res\_spi | li\_gci | li\_sector\_evenness |
| --- | --- | --- | --- | --- | --- | --- | --- | --- | --- | --- | --- | --- | --- | --- | --- | --- | --- | --- | --- | --- | --- |
| AO |  |  | x |  | x |  | x |  | x | x |  |  |  |  |  | x |  | x | x |  |  |
| CP | coral | x | x |  | x |  |  |  |  |  |  |  |  |  |  |  |  | x | x |  |  |
| CP | mangrove |  | x |  | x |  |  |  |  |  |  |  |  |  |  |  |  | x | x |  |  |
| CP | saltmarsh | x | x |  | x |  |  |  |  |  |  |  |  |  |  |  |  | x | x |  |  |
| CP | seagrass | x | x |  | x |  |  |  |  |  |  |  |  |  |  |  |  | x | x |  |  |
| CP | seaice\_shoreline |  |  |  |  |  |  |  |  |  |  |  |  |  |  |  |  | x | x |  |  |
| CS | mangrove |  | x |  | x |  |  |  |  |  |  |  |  |  |  |  |  | x | x |  |  |
| CS | saltmarsh | x | x |  | x |  |  |  |  |  |  |  |  |  |  |  |  | x | x |  |  |
| CS | seagrass | x | x |  | x |  |  |  |  |  |  |  |  |  |  |  |  | x | x |  |  |
| CW |  | x |  |  |  |  |  |  |  |  |  |  |  |  |  |  |  | x | x |  |  |
| ECO |  |  |  |  |  |  |  |  |  |  |  |  |  |  |  |  |  | x | x | x |  |
| FIS |  |  |  | x | x |  |  | x | x | x | x |  |  |  |  |  | x | x | x |  |  |
| HAB | coral | x | x |  | x |  | x |  | x |  | x | x | x |  |  | x |  | x | x |  |  |
| HAB | mangrove |  | x |  | x |  |  |  |  |  |  | x | x |  |  | x |  | x | x |  |  |
| HAB | saltmarsh | x | x |  | x |  |  |  |  |  |  | x | x |  |  | x |  | x | x |  |  |
| HAB | seagrass | x | x |  | x |  |  |  |  |  |  | x | x |  |  | x |  | x | x |  |  |
| HAB | seaice\_edge |  |  |  |  |  |  |  |  |  |  | x | x |  |  |  | x | x | x |  |  |
| HAB | soft\_bottom | x |  | x | x |  |  | x | x | x |  | x | x |  |  |  | x | x | x |  |  |
| ICO |  | x |  | x | x |  |  | x | x | x | x |  |  |  | x |  | x | x | x |  |  |
| SPP |  | x |  | x | x |  |  | x | x | x | x | x | x |  | x |  |  | x | x |  |  |
| LIV |  |  |  |  |  |  |  |  |  |  |  |  |  |  |  |  |  | x | x | x | x |
| LSP |  | x |  |  | x |  |  |  |  |  |  |  |  |  |  |  |  | x | x |  |  |
| MAR |  | x |  |  |  |  |  |  |  |  |  |  | x | x |  |  |  | x | x |  |  |
| NP | corals | x | x |  | x |  |  |  |  |  |  |  |  |  | x | x |  | x | x |  |  |
| NP | fish\_oil | x |  | x | x |  |  | x | x | x |  |  |  |  | x |  | x | x | x |  |  |
| NP | ornamentals | x | x |  | x |  | x |  | x |  | x |  |  |  | x | x |  | x | x |  |  |
| NP | seaweeds | x |  |  |  |  |  |  |  |  |  |  |  |  | x | x |  | x | x |  |  |
| NP | shells |  | x |  | x |  |  |  |  |  |  |  |  |  | x | x |  | x | x |  |  |
| NP | sponges |  | x |  | x |  |  |  |  |  |  |  |  |  | x | x |  | x | x |  |  |
| TR |  | x |  |  |  |  |  |  |  |  |  |  |  |  |  |  |  | x | x |  |  |

#### 4.3.3.1 Ecological resilience

##### 4.3.3.1.1 Ecosystem integrity

Ecosystem integrity, e.g., food web integrity, is measured as relative condition of assessed species in a given location (scores from the species subgoal were used to estimate ecosystem integrity). For some goals, there is little evidence that our index of ecosystem integrity directly affects the value of the goal (or subgoal). In these instances, ecological integrity falls out of the resilience model.

For the global assessments, we only have one data layer describing ecosystem integrity, however, if there were multiple layers the overall score for ecosystem integrity would be a weighted mean of all the data layers, \(i\), that describe ecosystem integrity (\(y\_{E,i}\)) and influence the goal:

\[
{ Y }\_{ E }\quad =\quad \frac { \displaystyle\sum \_{ i=1 }^{ N }{ { w }\_{ i }{ y\_E}\_{i} } }{ \displaystyle\sum \_{ i=1 }^{ N }{ { w }\_{ i }}}, (Eq. 4.11)
\]

##### 4.3.3.1.2 Regulatory resilience

Regulatory resilience (\(Y\_R\)) describes the institutional measures (e.g., rules, regulations, and laws) designed to address ecological pressures. The regulatory resilience datasets are grouped into five categories that address the 5 pressure categories: fishing pressure, habitat destruction, climate change (including ocean acidification), water pollution, and species introductions (invasive species and genetic escapes). There is also an additional category for goal-specific regulations that apply to a goal or goals, but do not address a larger pressure category.

Weights were based effectiveness of governance. Governance is a function of 1) institutional structures that address the intended objective, 2) a clear process for implementing the institution is in place, and 3) whether the institution has been effective at meeting stated objectives. At global scales it is very difficult to assess these three elements; we usually only had information on whether institutions exist. However, in some cases we had detailed information on institutions that enabled us to assess whether they would contribute to effective management, and thus, increased ocean health. In those latter cases, we gave more weight to those measures (Table 4.3).

For each region and goal, we calculated a score for each regulatory category, \(y\_{R, i}\), as a weighted mean of the resilience data layers, \(r\_{i}\), that influence the goal (Table 4.4):

\[
y\_{R, i}\quad =\quad \frac { \displaystyle\sum \_{ i=1 }^{ N }{ { w }\_{ i }{ r }\_{ i } } }{ \displaystyle\sum \_{ i=1 }^{ N }{w\_i} }, (Eq. 4.12)
\]

where, \(w\_i\) is the weight in Table 4.3.

To calculate the overall regulatory resilience, \(Y\_{R}\), we averaged the scores for each regulatory category.

#### 4.3.3.2 Social integrity resilience

Social integrity is intended to describe those processes internal to a community that affect its resilience. It is a function of a wide range of aspects of social structure. Social Integrity per goal for each region, \(Y\_{S}\), is therefore:

\[
Y\_{ S}\quad =\quad \frac { \displaystyle\sum \_{ i=1 }^{ N }{ { y }\_{ S,i } } }{ N }, (Eq. 4.13)
\]

where \(y\_{S,i}\) are the social integrity measures specific to the goal.

Ideally, assessments of social resilience would include state and federal level rules and other relevant institutional mechanisms as well. However, such information is extremely difficult to access for every single country, and so we relied on global datasets that focus on international treaties and assessments. Another key gap is information on social norms and community (and other local-scale) institutions (such as tenure or use rights) that influence resource use and management in many settings. Information on these institutions is also extremely difficult to find at a global scale, although the World Governance Indicator (Kaufmann *et al.* 2010) partly measures their effectiveness through its inclusion of corruption indices.

# 5 Goal models and data

In this section we describe how the current status and trend of each goal was calculated. We also indicate which data layers were used to calculate current status, trend (if different from current status), pressure, and resilience.

The R code used to calculate the goal model is located here (scroll to the appropriate goal function). To learn more about the data layers used in the model calculations see Section 6: Description of data layers. Table 6.1 includes links to the code and data used to create the data layers (current calculations are in the folder with the most recent year). Table 6.2 describes the data sources used to create the data layers.

## 5.1 Artisanal opportunities

Artisanal fishing, often also called small-scale fishing, provides a critical source of food, nutrition, poverty alleviation and livelihood opportunities for many people around the world, in particular in developing nations (Allison & Ellis 2001). Artisanal fishing refers to fisheries involving households, cooperatives or small firms (as opposed to large, commercial companies) that use relatively small amounts of capital and energy and small fishing vessels (if any), make relatively short fishing trips, and use fish mainly for local consumption or trade. These traits differ from commercial scale fisheries that serve the global fish trade, and commercial and artisanal scale fisheries also differ in how they are valued by many communities around the world.

Artisanal fisheries contribute over half of the world’s marine and inland fish catch, nearly all of which is used for direct human consumption (United Nations 2010). They employ over 90 percent of the world’s more than 35 million capture fishers and support another approximate 90 million people employed in jobs associated with fish processing, distribution and marketing (United Nations 2010). Artisanal fisheries also are distinguished by the role they play in shaping and sustaining human cultures around the world; this role contributes to their distinct value (McGoodwin 2001). For this reason, we designate artisanal fishing opportunities as a distinct public goal. In some countries like the U.S.A., artisanal fishing may happen under a commercial license (e.g., a family run lobster boat or individual shellfish harvesting permit), or under a recreational fishing permit (e.g., families fishing with rods for fish to eat); the food provided by these activities should ideally be captured under the food provision goal, whereas the opportunity to pursue artisanal fishing is captured here. The goal is not about recreational fishing for sport, which is captured in food provision (if it provides food) and tourism and recreation.

The livelihood and household economy provided by fishing are considered part of the coastal livelihoods and economies goal, although similar to food provision from artisanal fishing it is currently impossible to measure on a global scale. Our focus is on the opportunity to conduct this kind of fishing. What is intended by the idea of ‘opportunity’ is the ability to conduct sustainable artisanal-scale fishing when the need is present, rather than the actual amount of catch or household revenue that is generated. Although this may seem nuanced on the value and intent of artisanal fishing, the opportunity to conduct this fishing is clearly of great importance to many people (McGoodwin 2001). Status for this goal is a function of need for artisanal fishing opportunities and whether or not the opportunity is permitted and/or encouraged institutionally and done sustainability. This need could potentially be driven by any number of socio-economic factors, but perhaps the simplest and most directly tied to this need is the percent of the population that is below the poverty level. Data on how many people live below the poverty level are not available for many countries. Therefore, we used an analogous proxy that is more complete globally: per capita gross domestic product (pcGDP) adjusted by the purchasing power parity (PPP). This metric translates the average annual income (pcGDP) into its local value (PPP). These data correlate with UN data on the percent of a population living below the $2/day international poverty standard (linear: R2 = 0.61, p <0.001; logarithmic regression: R2 = 0.76, p < 0.001). Because the relationship is a better fit with the ln-linear regression, we ln-transform the PPPpcGDP scores.

#### 5.1.0.1 Current status

Status for this goal (\(x\_{ao}\)) is therefore measured by unmet demand (\(D\_u\)), which includes measures of opportunity for artisanal fishing (\(O\_{ao}\), defined below) and the sustainability of the methods used (\(S\_{ao}\)):

\[
x\_{ao} = (1 – D\_u) \* S\_{ao}, (Eq. 5.1)
\]

where \(S\_{ao}\) indicates whether artisanal fishing is done in a sustainable manner, and is set to 1 because no data or information exist globally on the proportion of fishermen using sustainable versus unsustainable gear, \(S\_{ao}\). And, \(D\_u\) is calculated as:

\[
D\_{u} = (1 – PPPpcGDP) \* (1 – O\_{ao}), (Eq. 5.2)
\]

where, \(PPPpcGDP\) is the ln-transformed, rescaled purchasing power parity adjusted per capita GDP, and \(O\_{ao}\) is the access to artisanal-scale fishing determined by Mora et al. (2009).

We rescaled the ln-transformed \(PPPpcGDP\) values from 0-1 by dividing by the value corresponding to the 99th quantile across all regions and years from 2005 to 2015 (values > 1 were capped at 1). Developed countries with lower demand for artisanal scale fishing (i.e., low poverty indicated by high PPPpcGDP) score high, regardless of the opportunity made available (since it would not matter to many), and developing countries with high demand and opportunity would also score high.

To assess the opportunity or ability to meet this demand, \(O\_{ao}\), we used data from Mora et al. (2009), which scores countries on the institutional measures that support or facilitate artisanal and small-scale fishing. The data come from Figure S4 in Mora et al. (2009), which is based on two survey questions focused on recreational and artisanal fishing (Table 5.1) and are on a scale from 0 to 100 (which we then rescale 0-1), where higher scores indicate better management. We extracted the data from the color codes on the map in Figure S4 in Mora et al. (2009). There may be some small errors far a few countries due to difficulty of distinguishing between the two red colors at the lowest end of the scale.

The sustainability of artisanal fishing practices could be approximated by the percent of fishermen that use sustainable gear such as hook and line versus unsustainable methods such as dynamite, cyanide and, arguably, gill net fishing. Unfortunately data on proportion of gear type used within a country is scarce at best and so we were unable to include this term in the calculation of this goal; we present it here for conceptual completeness. We considered using the information and data contained in Pitcher et al. (2006) which looks at compliance of 53 countries with the UN’s Code of Conduct for responsible fisheries. These results are strongly correlated with the data from Mora et al. (2009) (p < 0.001; R2 = 0.22), and thus we used the Mora et al.

Several issues and datasets relevant to artisanal fishing opportunities were not included in our calculations for a number of reasons. High unemployment can lead to a greater demand for artisanal fishing opportunities (Cinner *et al.* 2009), but unemployment is not a good measure of potential ‘demand’ for most developing countries since many people not working do not get recorded in unemployment statistics, even though it may be relevant for developed countries. Regardless, it is very difficult to set an arbitrary cut-off for developing versus developed countries, and so there is no clear way to use unemployment data for this goal.

Another potential driver of demand for artisanal fishing opportunities is local preference for seafood and/or access to other sources of protein. Previous analyses have shown that seafood consumption (a proxy for preference for seafood) does not correlate well with national-level artisanal catch statistics (Halpern *et al.* 2008) and access to other sources of protein is difficult to measure, and so we did not use either of these measures here.

**Table 5.1. Artisanal access**. Questions from Mora et al. (2009) that were used to evaluate access to artisanal scale fishing.

- If recreational fishing exists to any extent, which of the following apply?
  - Are recreational fishermen required to have a fishing license?
  - Are there regulations to the size of fish caught?
  - Are there regulations to the number of fish caught?
  - Are there regulations to the number of fishermen allowed to fish?
  - Are there statistics being collected for this sort of fishing? \*If artisanal fishing exists to any extent, which of the following apply?
  - Are there regulations to the size of fish caught?
  - Are there regulations to the number of fish caught?
  - Are there regulations to the number of fishermen allowed to fish?
  - Are there statistics being collected for this sort of fishing?

#### 5.1.0.2 Trend

Trend was calculated as described in section 4.3.1. Because we only have one value for \(O\_{ao}\), the trend becomes the change over time in the PPPpcGDP, i.e., how ‘unmet demand’ is changing over time.

#### 5.1.0.3 Data

*Status and trend*

Artisanal fisheries opportunity (ao\_access): The opportunity for artisanal and recreational fishing based on the quality of management of the small-scale fishing sector

Economic need for artisanal fishing (ao\_need): Per capita purchasing power parity (PPP) adjusted gross domestic product (GDP): GDPpcPPP as a proxy for subsistence fishing need

*Pressure*

Coastal chemical pollution (po\_chemicals\_3nm): Modeled chemical pollution within 3nm of coastline from commercial shipping traffic, ports and harbors, land-based pesticide use (organic pollution), and urban runoff (inorganic pollution)

Coastal nutrient pollution (po\_nutrients\_3nm): Modeled nutrient pollution within EEZ based on fertilizer consumption

High bycatch due to artisanal fishing (fp\_art\_hb): The presence of destructive artisanal blast and poison (cyanide) fishing.

High bycatch due to commercial fishing (fp\_com\_hb): Modeled destructive commercial fishing practices by 5 gear types and scaled by Net Primary Productivity

Intertidal habitat destruction (hd\_intertidal): Coastal population density (25 mi from shore) as a proxy for intertidal habitat destruction

Low bycatch due to commercial fishing (fp\_com\_lb): Modeled destructive commercial fishing practices by 2 gear types and scaled by Net Primary Productivity

Nonindigenous species (sp\_alien): Measure of harmful invasive species

Subtidal hardbottom habitat destruction (hd\_subtidal\_hb): High bycatch artisanal fishing practices (blast fishing) as a proxy for subtidal hard bottom habitat destruction

Subtidal soft bottom habitat destruction (hd\_subtidal\_sb): Demersal destructive commercial fishing practices (i.e., trawling) in soft bottom habitat as a proxy for soft bottom habitat destruction

Weakness of governance (ss\_wgi): Inverse of World Governance Indicators (WGI) six combined scores

Weakness of social progress (ss\_spi): Inverse of Social Progress Index scores

*Resilience*

Coastal protected marine areas (fishing preservation) (fp\_mpa\_coast): Protected marine areas within 3nm of coastline (lasting special places goal status score)

Coastal protected marine areas (habitat preservation) (hd\_mpa\_coast): Protected marine areas within 3nm of coastline (lasting special places goal status score)

Commercial fishing management (fp\_mora): Regulations and management of commerical fishing

Management of habitat to protect fisheries biodiversity (fp\_habitat): Survey responses by country to the Convention on Biological Diversity (CBD) Third National Report: habitat related questions

Management of habitat to protect habitat biodiversity (hd\_habitat): Survey responses by country to the Convention on Biological Diversity (CBD) Third National Report: habitat related questions

Measure of coastal ecological integrity (species\_diversity\_3nm): Marine species condition (same calculation and data as the species subgoal status score) calculated within 3 nm of shoreline as a proxy for ecological integrity

Social Progress Index (res\_spi): Social Progress Index scores

Strength of governance (wgi\_all): World Governance Indicators (WGI) six combined scores

## 5.2 Biodiversity

People value biodiversity in particular for its existence value. The risk of species extinction generates great emotional and moral concern for many people. As such, this goal assesses the conservation status of species based on the best available global data through two sub-goals: species and habitats. Species were assessed because they are what one typically thinks of in relation to biodiversity. Because only a small proportion of marine species worldwide have been mapped and assessed, we also assessed habitats as part of this goal, and considered them a proxy for condition of the broad suite of species that depend on them. For the species sub-goal, we used species risk assessments from the International Union for Conservation of Nature (IUCN 2016a) for a wide range of taxa to provide a geographic snapshot of how total marine biodiversity is faring, even though it is a very small sub-sample of overall species diversity (Mora *et al.* 2011). We calculate each of these subgoals separately and weight them equally when calculating the overall goal score.

### 5.2.1 Habitat (subgoal of biodiversity)

The habitat subgoal measures the average condition of marine habitats present in each region that provide critical habitat for a broad range of species (mangroves, coral reefs, seagrass beds, salt marshes, sea ice edge, and subtidal soft bottom). This subgoal is considered a proxy for the condition of the broad suite of marine species.

Data availability remains a major challenge for species and habitat assessments. We compiled and analyzed the best available data in both cases, but key gaps remain. Although several efforts have been made in recent years to create or compile the data necessary to look at the status and trends of marine habitats, most efforts are still hampered by limited geographical and temporal sampling (Joppa *et al.* 2016), although mangroves (Giri *et al.* 2011) and sea ice (Cavalieri *et al.* 1996) data are exceptions. In addition, most marine habitats have only been monitored since the late 1970s at the earliest, many sites were only sampled over a short period of time, and very few sites were monitored before the late 1990s so establishing reference points was difficult. Salt marshes and seagrasses were the most data-limited of the habitats included in the analysis.

#### 5.2.1.1 Current status

The status of the habitat sub-goal, \(x\_{hab}\), was assessed as the average of the condition estimates, \(C\), for each habitat, \(k\), present in a region; measured as the loss of habitat and/or % degradation of remaining habitat, such that:

\[
x\_{hab} = \frac { \displaystyle\sum \_{ k=1 }^{ N }{ { C }\_{ k } } }{ N}, \quad \quad (Eq. 5.3)
\]

where, \(C\_{k} = C\_{c}/C\_{r}\) and \(N\) is the number of habitats in a region. \(C\_{c}\) is the current condition and \(C\_{r}\) is the reference condition specific to each \(k\) habitat present in the region (Table 5.2). This formulation ensures that each country is assessed only for those habitats that can exist (e.g., Canada is not assessed on the status of its nonexistent coral reefs). We generally considered the reference years to be between 1980-1995 and the current years to be between 2001-2010, although these varied by habitat due to data availability.

**Table 5.2. Habitat data** Description of condition, extent, and trend calculations for habitat data (Note: extent is not used to calculate the habitat subgoal, but is used for the coastal protection and carbon storage goals). More information about the sources used to generate these values is located in Section 6 and Table 6.1.

| Habitat | Condition | Extent | Trend |
| --- | --- | --- | --- |
| Seagrass | Current % cover or hectares of habitat divided by reference % cover or hectares | Seagrass extent per oceanic region (vector based) | Calculated across data from 1975-2010 |
| Coral reefs | Current % cover divided by reference % cover | Coral reef extent per oceanic region (500 m resolution) | Calculated across data from 1975-2006 |
| Mangroves | Current hectares divided by reference hectares, for coastal mangroves only | Mangrove extent per oceanic region (raster based) | Calculated using 5 most recent years of data |
| Salt marsh | Increasing or stable trend assigned condition = 1.0; decreasing trend assigned condition = 0.5 | Salt marsh extent per oceanic region | Categorical trend assessments (increasing = 0.5, stable = 0, or decreasing = -0.5) |
| Sea ice edge | Current % cover of sea ice with 10-50% cover divided by reference % cover average from 1979-2000 | Same as condition | Calculated from the fitted slope of % deviation from reference per year of most recent 5 years of data (each year of data is based on 3-year average) |
| Soft bottom | Soft-bottom destructive fishing practices rescaled to 0-1 using the 99.99th quantile at the 0.5\(^{\circ}\) scale and averaged per region | Halpern et al. (2008) | Calculated using 5 most recent years of condition data |

A significant amount of pre-processing of the habitat data was needed to fill data gaps and resolve data quality issues (Section 6). Because consistent habitat monitoring data was unavailable for many countries, anomalous values can occur. This is particularly true for highly variable habitats like seagrasses or coral reefs which can have significant site-to-site and year-to-year differences in extent and condition (Orth *et al.* 2006; Bruno & Selig 2007). Biases may also have been introduced from spatial (e.g., protected or impacted sites) and temporal (e.g., directly after a disturbance event) selections in sampling. In regions where we had a limited number of surveys in a particular country, overall status can be under- or overestimated because of these fluctuations.

#### 5.2.1.2 Trend

Trend in habitat data were calculated as the linear trend in extent or condition with slight variations depending on habitat type. Coral reef habitat trends were calculated on a per country basis, using all available data. For seagrasses we calculated trends on a per site basis. For mangroves we used the rate of change in areal extent over the most recent 5 years of available data. For sea ice we calculated the slope across the most recent 5 years of data, where each year of data is based on a three-year moving averages to smooth out potential climate variation. For soft bottom habitat we simply calculated the slope of the recent change in condition over the past five years, i.e., the change in proportion of catch from trawl fishing per unit area of habitat within a region.

#### 5.2.1.3 Data

*Status and trend*

Habitat condition (hab\_health): Current condition of habitat relative to historical condition

Habitat condition trend (hab\_trend): Estimated change in habitat condition

Habitat extent (hab\_extent): Area of habitats: mangrove, saltmarsh, seagrass, soft bottom, seaice, coral

*Pressure*

Coastal chemical pollution (po\_chemicals\_3nm): Modeled chemical pollution within 3nm of coastline from commercial shipping traffic, ports and harbors, land-based pesticide use (organic pollution), and urban runoff (inorganic pollution)

Coastal nutrient pollution (po\_nutrients\_3nm): Modeled nutrient pollution within EEZ based on fertilizer consumption

High bycatch due to artisanal fishing (fp\_art\_hb): The presence of destructive artisanal blast and poison (cyanide) fishing.

High bycatch due to commercial fishing (fp\_com\_hb): Modeled destructive commercial fishing practices by 5 gear types and scaled by Net Primary Productivity

Intertidal habitat destruction (hd\_intertidal): Coastal population density (25 mi from shore) as a proxy for intertidal habitat destruction

Low bycatch due to artisanal fishing (fp\_art\_lb): Extent of artisanal fishing (including: artisanal, subsistence, and recreational catch)

Low bycatch due to commercial fishing (fp\_com\_lb): Modeled destructive commercial fishing practices by 2 gear types and scaled by Net Primary Productivity

Nonindigenous species (sp\_alien): Measure of harmful invasive species

Ocean acidification (cc\_acid): Ocean acidification pressure scaled using biological thresholds

Sea level rise (cc\_slr): Sea level rise pressure

Sea surface temperature (cc\_sst): Sea surface temperature anomalies

Subtidal hardbottom habitat destruction (hd\_subtidal\_hb): High bycatch artisanal fishing practices (blast fishing) as a proxy for subtidal hard bottom habitat destruction

Subtidal soft bottom habitat destruction (hd\_subtidal\_sb): Demersal destructive commercial fishing practices (i.e., trawling) in soft bottom habitat as a proxy for soft bottom habitat destruction

UV radiation (cc\_uv): Modeled UV radiation

Weakness of governance (ss\_wgi): Inverse of World Governance Indicators (WGI) six combined scores

Weakness of social progress (ss\_spi): Inverse of Social Progress Index scores

*Resilience*

Artisanal fisheries management effectiveness (fp\_mora\_artisanal): Quality of management of small-scale fishing for artisanal and recreational purposes

Coastal protected marine areas (fishing preservation) (fp\_mpa\_coast): Protected marine areas within 3nm of coastline (lasting special places goal status score)

Coastal protected marine areas (habitat preservation) (hd\_mpa\_coast): Protected marine areas within 3nm of coastline (lasting special places goal status score)

Commercial fishing management (fp\_mora): Regulations and management of commerical fishing

EEZ protected marine areas (fishing preservation) (fp\_mpa\_eez): Protected marine areas within EEZ (lasting special places calculation applied to the entire EEZ)

EEZ protected marine areas (habitat preservation) (hd\_mpa\_eez): Protected marine areas within EEZ (lasting special places calculation applied to the entire EEZ)

Management of habitat to protect fisheries biodiversity (fp\_habitat): Survey responses by country to the Convention on Biological Diversity (CBD) Third National Report: habitat related questions

Management of habitat to protect habitat biodiversity (hd\_habitat): Survey responses by country to the Convention on Biological Diversity (CBD) Third National Report: habitat related questions

Management of mariculture to preserve biodiversity (g\_mariculture): Survey responses by country to the Convention on Biological Diversity (CBD) Third National Report: mariculture related questions

Management of tourism to preserve biodiversity (g\_tourism): Survey responses by country to the Convention on Biological Diversity (CBD) Third National Report: tourism related questions

Management of waters to preserve biodiversity (po\_water): Survey responses by country to the Convention on Biological Diversity (CBD) Third National Report: tourism related questions

Measure of coastal ecological integrity (species\_diversity\_3nm): Marine species condition (same calculation and data as the species subgoal status score) calculated within 3 nm of shoreline as a proxy for ecological integrity

Measure of ecological integrity (species\_diversity\_eez): Marine species condition (species subgoal status score) as a proxy for ecological integrity

Social Progress Index (res\_spi): Social Progress Index scores

Strength of governance (wgi\_all): World Governance Indicators (WGI) six combined scores

### 5.2.2 Species condition (subgoal of biodiversity)

This goal aims to assess the average condition of the marine species within each region based on IUCN status (IUCN 2016a). The target for the species subgoal is to have all species at a risk status of Least Concern.

#### 5.2.2.1 Current status

Species status was calculated as the area and status-weighted average of assessed species within each region. Marine species distribution and threat category data mostly came from IUCN Red List of Threatened Species, and we limited data to all species having IUCN habitat system of “marine” (IUCN 2016a). Seabird distributions data came from Birdlife International (‘Bird species distribution maps of the world’ 2015). When neither IUCN nor BirdLife International distributions were available, we supplemented with AquaMaps (Kaschner *et al.* 2015) using any non-zero probability of occurrence to indicate species presence to create a binary range map. Range maps were analyzed at the 0.5 degree scale.

We scaled the lower end of the biodiversity goal to be 0 when 75% species are extinct, a level comparable to the five documented mass extinctions (Barnosky *et al.* 2011) and would constitute a catastrophic loss of biodiversity.

Threat weights, \(w\_{i}\), were assigned based on the IUCN threat categories status of each \(i\) species, following the weighting schemes developed by Butchart et al. (2007) (Table 5.3). For the purposes of this analysis, we included only data for extant species for which sufficient data were available to conduct an assessment. We did not include the Data Deficient classification as assessed species following previously published guidelines for a mid-point approach (Schipper *et al.* 2008; Hoffmann *et al.* 2010).

We first calculated each the region’s area-weighted average species risk status, \(\bar R\_{spp}\). For each 0.5 degree grid cell within a region, \(c\), the risk status, \(w\), for each species, \(i\), present is summed and multiplied by cell area \(A\_c\), to get an area- and count-weighted species risk for each cell. This value is then divided by the sum of count-weighted area \(A\_c \times N\_c\) across all cells within the region. The result is the area-weighted mean species risk across the entire region.

\[
\bar R\_{spp} = \frac { \displaystyle\sum\_{ c=1 }^{ M } \left( \displaystyle\sum \_{ i=1 }^{N\_c} w\_i \right) \times A\_c }
{ \displaystyle\sum\_{ c=1 }^{ M } A\_c \times N\_c }, (Eq. 5.4)
\] To convert \(\bar R\_{spp}\) into a score, we set a floor at 25% (representing a catastrophic loss of biodiversity, as noted above) and then rescaled to produce a \(x\_{spp}\) value between zero and one.

\[
x\_{spp} = max \left( \frac { \bar R\_{SPP} - .25 }{ .75 }, 0 \right), (Eq. 5.5)
\]

**Table 5.3. Weights for assessment of species status based on IUCN risk categories**

| Risk Category | IUCN code | Weight |
| --- | --- | --- |
| Extinct | EX | 0.0 |
| Critically Endangered | CR | 0.2 |
| Endangered | EN | 0.4 |
| Vulnerable | VU | 0.6 |
| Near Threatened | NT | 0.8 |
| Least Concern | LC | 1.0 |

#### 5.2.2.2 Trend

We calculated trend as the average of the population trend assessments for all species within a region, with species’ trends assigned a value of 0.5 for increasing, 0 for stable, and -0.5 for decreasing using the population trend data associated with the species assessment conducted by IUCN.

#### 5.2.2.3 Data

*Status and trend*

Average species condition (spp\_status): Species condition based on average of IUCN threat categories

Average species condition trend (spp\_trend): Species trends based on average of IUCN population trend data

*Pressure*

Chemical pollution (po\_chemicals): Modeled chemical pollution within EEZ from commercial shipping traffic, ports and harbors, land-based pesticide use (organic pollution), and urban runoff (inorganic pollution)

Genetic escapes (sp\_genetic): Introduced mariculture species (Mariculture Sustainability Index) as a proxy for genetic escapes

High bycatch due to artisanal fishing (fp\_art\_hb): The presence of destructive artisanal blast and poison (cyanide) fishing.

High bycatch due to commercial fishing (fp\_com\_hb): Modeled destructive commercial fishing practices by 5 gear types and scaled by Net Primary Productivity

Intertidal habitat destruction (hd\_intertidal): Coastal population density (25 mi from shore) as a proxy for intertidal habitat destruction

Low bycatch due to artisanal fishing (fp\_art\_lb): Extent of artisanal fishing (including: artisanal, subsistence, and recreational catch)

Low bycatch due to commercial fishing (fp\_com\_lb): Modeled destructive commercial fishing practices by 2 gear types and scaled by Net Primary Productivity

Marine plastics (po\_trash): Global marine plastic pollution

Nonindigenous species (sp\_alien): Measure of harmful invasive species

Nutrient pollution (po\_nutrients): Modeled nutrient pollution within 3nm of coastline based on fertilizer consumption

Ocean acidification (cc\_acid): Ocean acidification pressure scaled using biological thresholds

Sea surface temperature (cc\_sst): Sea surface temperature anomalies

Subtidal hardbottom habitat destruction (hd\_subtidal\_hb): High bycatch artisanal fishing practices (blast fishing) as a proxy for subtidal hard bottom habitat destruction

Subtidal soft bottom habitat destruction (hd\_subtidal\_sb): Demersal destructive commercial fishing practices (i.e., trawling) in soft bottom habitat as a proxy for soft bottom habitat destruction

Targeted harvest of cetaceans and marine turtles (fp\_targetharvest): Targeted harvest of cetaceans and marine turtles

UV radiation (cc\_uv): Modeled UV radiation

Weakness of governance (ss\_wgi): Inverse of World Governance Indicators (WGI) six combined scores

Weakness of social progress (ss\_spi): Inverse of Social Progress Index scores

*Resilience*

Artisanal fisheries management effectiveness (fp\_mora\_artisanal): Quality of management of small-scale fishing for artisanal and recreational purposes

CITES signatories (g\_cites): Convention on International Trade in Endangered Species of Wild Fauna and Flora (CITES) signatories

Commercial fishing management (fp\_mora): Regulations and management of commerical fishing

EEZ protected marine areas (fishing preservation) (fp\_mpa\_eez): Protected marine areas within EEZ (lasting special places calculation applied to the entire EEZ)

EEZ protected marine areas (habitat preservation) (hd\_mpa\_eez): Protected marine areas within EEZ (lasting special places calculation applied to the entire EEZ)

Management of habitat to protect fisheries biodiversity (fp\_habitat): Survey responses by country to the Convention on Biological Diversity (CBD) Third National Report: habitat related questions

Management of habitat to protect habitat biodiversity (hd\_habitat): Survey responses by country to the Convention on Biological Diversity (CBD) Third National Report: habitat related questions

Management of mariculture to preserve biodiversity (g\_mariculture): Survey responses by country to the Convention on Biological Diversity (CBD) Third National Report: mariculture related questions

Management of tourism to preserve biodiversity (g\_tourism): Survey responses by country to the Convention on Biological Diversity (CBD) Third National Report: tourism related questions

Management of waters to preserve biodiversity (po\_water): Survey responses by country to the Convention on Biological Diversity (CBD) Third National Report: tourism related questions

Social Progress Index (res\_spi): Social Progress Index scores

Strength of governance (wgi\_all): World Governance Indicators (WGI) six combined scores

## 5.3 Coastal protection

This goal aims to assess the amount of protection provided by marine and coastal habitats to coastal areas that people value, both inhabited (homes and other structures) and uninhabited (parks, special places, etc.). At local and regional scales data may exist on all these variables at a high enough resolution to map and calculate exactly which habitats are providing how much protection to which coastal areas. At global scales, such data do not exist and so we focused on EEZ-scale assessments, even though this scale does not allow one to account for the spatial configuration of habitats relative to coastal areas and human populations. Consequently, we assumed that all coastal areas have value (and equal value) and assessed the total area and condition of key habitats within each EEZ (without regard to their precise location relative to coastal areas). The habitats that provide protection to coastal areas for which we have global data include mangroves, coral reefs, seagrasses, salt marshes (Table 5.2), and coastal sea ice (shoreline pixels with >15% ice cover).

#### 5.3.0.1 Current status

The status of this goal, \(x\_{cp}\), was calculated to be a function of the relative health of the habitats, \(k\), within a region that provide shoreline protection, weighted by their area and protectiveness rank (Table 5.4), such that:

\[
x\_{cp} = \frac { \displaystyle\sum \_{ k=1 }^{ N }{ { (h }\_{ k } } \times { w }\_{ k }\times { A }\_{ k }) }{ \displaystyle\sum \_{ k=1 }^{ N }{ { (w }\_{ k }\times { A }\_{ k }) } }, (Eq. 5.6)
\]

where, \(w\) is the rank weight of the habitat’s protective ability, \(A\) is the area within a region for each \(k\) habitat type, and \(h\) is a measure of each habitat’s condition:

\[
h = \frac { C\_{ c } }{ { C }\_{ r } }
\]

where, \(C\_c\) is current condition and \(C\_r\) is reference condition.

**Table 5.4. Coastal protectiveness ranks** Scores range from 1-4, with 4 being the most protective (Tallis *et al.* 2011).

| Habitat | Protectiveness rank (\(w\)) |
| --- | --- |
| mangroves | 4 |
| salt marshes | 4 |
| coastal sea ice | 4 |
| coral reefs | 3 |
| seagrasses | 1 |

The reference area for each habitat is treated as a fixed value; in cases where current area might exceed this reference value (e.g., through restoration), we cap the score at the maximum value (1.0). Although this does not give credit for restoration, data tend to be of poor quality making it difficult to determine true increases, and in general habitat restoration beyond reference values is extremely unlikely. Rank weights for the protective ability of each habitat (\(w\_{k}\)) come from previous work (Tallis *et al.* 2011).

#### 5.3.0.2 Trend

The trend for this goal is calculated using different methods for each habitat due to data availability (Table S8, with sea ice shoreline following the same general methods as sea ice edge).

#### 5.3.0.3 Data

*Status and trend*

Habitat condition (hab\_health): Current condition of habitat relative to historical condition

Habitat condition trend (hab\_trend): Estimated change in habitat condition

Habitat extent (hab\_extent): Area of habitats: mangrove, saltmarsh, seagrass, soft bottom, seaice, coral

*Pressure*

Coastal chemical pollution (po\_chemicals\_3nm): Modeled chemical pollution within 3nm of coastline from commercial shipping traffic, ports and harbors, land-based pesticide use (organic pollution), and urban runoff (inorganic pollution)

Coastal nutrient pollution (po\_nutrients\_3nm): Modeled nutrient pollution within EEZ based on fertilizer consumption

Intertidal habitat destruction (hd\_intertidal): Coastal population density (25 mi from shore) as a proxy for intertidal habitat destruction

Nonindigenous species (sp\_alien): Measure of harmful invasive species

Ocean acidification (cc\_acid): Ocean acidification pressure scaled using biological thresholds

Sea level rise (cc\_slr): Sea level rise pressure

Sea surface temperature (cc\_sst): Sea surface temperature anomalies

Subtidal hardbottom habitat destruction (hd\_subtidal\_hb): High bycatch artisanal fishing practices (blast fishing) as a proxy for subtidal hard bottom habitat destruction

UV radiation (cc\_uv): Modeled UV radiation

Weakness of governance (ss\_wgi): Inverse of World Governance Indicators (WGI) six combined scores

Weakness of social progress (ss\_spi): Inverse of Social Progress Index scores

*Resilience*

Coastal protected marine areas (habitat preservation) (hd\_mpa\_coast): Protected marine areas within 3nm of coastline (lasting special places goal status score)

Management of habitat to protect habitat biodiversity (hd\_habitat): Survey responses by country to the Convention on Biological Diversity (CBD) Third National Report: habitat related questions

Management of waters to preserve biodiversity (po\_water): Survey responses by country to the Convention on Biological Diversity (CBD) Third National Report: tourism related questions

Social Progress Index (res\_spi): Social Progress Index scores

Strength of governance (wgi\_all): World Governance Indicators (WGI) six combined scores

## 5.4 Carbon storage

The present-day pelagic ocean sink for anthropogenic carbon dioxide, estimated at approximately 2000 TgC yr-1, accounts for about a quarter of total anthropogenic CO2 emissions to the atmosphere and helps mitigate a key driver of global climate change (Le Quéré *et al.* 2009; Sabine & Tanhua 2010). The physical-chemical mechanisms driving the ocean sink are well understood but are not directly amenable to human management. Highly productive coastal wetland ecosystems (e.g., mangroves, salt marshes, seagrass beds) have substantially larger areal carbon burial rates than terrestrial forests, and “Blue Carbon” has been suggested as an alternate, more manageable carbon sequestration approach. The rapid destruction of these coastal habitats may release large amounts of buried carbon back into the ocean-atmosphere system. Donato and colleagues (2011), for example, estimate that mangrove deforestation generates emissions of 20-120 TgC yr-1. Our focus here, therefore, is on coastal habitats because they are threatened, have large amounts of stored carbon that would rapidly be released with further habitat destruction, have the highest per-area sequestration rates of any habitat on the planet, and are amenable to management, conservation, and restoration efforts. We refer to this goal as carbon storage but intend its meaning to include sequestration.

We focused on three coastal habitats known to provide meaningful amounts of carbon storage (Table 5.2): mangroves, seagrasses, and salt marshes (Duarte 2000). For mangroves, we used coastal mangroves that are on land or in river deltas.

#### 5.4.0.1 Current status

We measured the status of carbon storage, \(x\_{cs}\), as a function of the carbon storing habitats’ current condition, \(C\_{c}\), relative to their reference condition, \(C\_{r}\). The habitat condition values were averaged, weighted by the area of each habitat, \(A\_{k}\), and a coefficient, \(w\_k\), to account for the relative contribution of each habitat type, \(k\), to total carbon storage (Laffoley & Grimsditch 2009) (Table 5.5):

\[
x\_{cs} = \frac { \displaystyle\sum \_{ k=1 }^{ N }{ { (h }\_{ k } } \times { w }\_{ k }\times { A }\_{ k }) }{ \displaystyle\sum \_{ k=1 }^{ N }{ { (w }\_{ k }\times { A }\_{ k }) } }, (Eq. 5.7)
\]

where:

\[
h = \frac { C\_{ c } }{ { C }\_{ r } }
\]

We employed several different methods for calculating habitat condition scores depending on the habitat of interest and available data (Table 5.2).

**Table 5.5. Carbon sequestration data** Weighting factors based on carbon sequestration rates for habitats used in the carbon storage goal (Laffoley & Grimsditch 2009).

| Habitat carbon storage | Sequestration (weight) |
| --- | --- |
| Mangrove | 139 |
| Saltmarsh | 210 |
| Seagrass | 83 |

We scaled each region’s score to habitat area for two reasons. First, it avoids penalizing a country that naturally lacks one of the habitats (e.g., Canada is too cold to have mangroves). Second, it ensures that habitats influence the goal score proportionately to their area of extent. This rewards the protection of large extents of habitat but does not assign a higher weight to higher habitat diversity. As such, our measure underestimates the actual amount of carbon storage being done by these coastal habitats (because we cannot account for habitats we do not know exist).

Reference area for each habitat is treated as a fixed value; in cases where current area might exceed this reference value (e.g., through restoration), we cap the score at the maximum value (1.0). Although this does not give credit for restoration efforts improving things, data tend to be of poor quality making it difficult to determine true increases, and in general habitat restoration beyond reference values is extremely unlikely.

### 5.4.1 Trend

The trend for this goal is calculated using different methods for each habitat due to data availability (Table 5.2).

#### 5.4.1.1 Data

*Status and trend*

Habitat condition (hab\_health): Current condition of habitat relative to historical condition

Habitat condition trend (hab\_trend): Estimated change in habitat condition

Habitat extent (hab\_extent): Area of habitats: mangrove, saltmarsh, seagrass, soft bottom, seaice, coral

*Pressure*

Coastal chemical pollution (po\_chemicals\_3nm): Modeled chemical pollution within 3nm of coastline from commercial shipping traffic, ports and harbors, land-based pesticide use (organic pollution), and urban runoff (inorganic pollution)

Coastal nutrient pollution (po\_nutrients\_3nm): Modeled nutrient pollution within EEZ based on fertilizer consumption

Intertidal habitat destruction (hd\_intertidal): Coastal population density (25 mi from shore) as a proxy for intertidal habitat destruction

Nonindigenous species (sp\_alien): Measure of harmful invasive species

Ocean acidification (cc\_acid): Ocean acidification pressure scaled using biological thresholds

Sea level rise (cc\_slr): Sea level rise pressure

Sea surface temperature (cc\_sst): Sea surface temperature anomalies

Weakness of governance (ss\_wgi): Inverse of World Governance Indicators (WGI) six combined scores

Weakness of social progress (ss\_spi): Inverse of Social Progress Index scores

*Resilience*

Coastal protected marine areas (habitat preservation) (hd\_mpa\_coast): Protected marine areas within 3nm of coastline (lasting special places goal status score)

Management of habitat to protect habitat biodiversity (hd\_habitat): Survey responses by country to the Convention on Biological Diversity (CBD) Third National Report: habitat related questions

Management of waters to preserve biodiversity (po\_water): Survey responses by country to the Convention on Biological Diversity (CBD) Third National Report: tourism related questions

Social Progress Index (res\_spi): Social Progress Index scores

Strength of governance (wgi\_all): World Governance Indicators (WGI) six combined scores

## 5.5 Clean waters

People value marine waters that are free of pollution and debris for aesthetic and health reasons. Contamination of waters comes from oil spills, chemicals, eutrophication, algal blooms, disease pathogens (e.g., fecal coliform, viruses, and parasites from sewage outflow), floating trash, and mass kills of organisms due to pollution. People are sensitive to these phenomena occurring in areas they access for recreation or other purposes as well as for simply knowing that clean waters exist. This goal scores highest when the contamination level is zero.

We include four measures of pollution in the clean waters goal: eutrophication (nutrients), chemicals, pathogens and marine debris. This decision was meant to represent a comprehensive list of the contamination categories that are commonly considered in assessments of coastal clean waters (Borja *et al.* 2008) and for which we could obtain datasets (Table 5.6). The status of these components is the inverse of their intensity (i.e., high input results in low status score).

**Table 5.6. Clean waters goal components**

| Component | Data | Trend |
| --- | --- | --- |
| Eutrophication (nutrient) | FAO fertilizer data (Halpern *et al.* 2008; United Nations 2016c) | Standard method (section 4.3.1) |
| Chemical | Land-based organic pollution (FAO pesticide data), Land-based inorganic pollution (based on run-off from impermeable surfaces), ocean-based pollution based on commercial shipping and port traffic (Halpern *et al.* 2008; United Nations 2016c) | Trend based only on changes in organic pollution, other variables remained the same |
| Pathogens | Proportion of population without access to improved sanitation facilities (WHO-UNICEF 2015) | Standard method |
| Marine debris | Plastic pollution (Eriksen *et al.* 2014) | Data on improperly disposed of plastics (Jambeck *et al.* 2015) |

We used the modeled input of land-based nitrogen input from Halpern et al. (2008) as a proxy for nutrient input. The modeled proxy approach does not allow the distinction between toxic and non-toxic bloom events that can arise from excess nutrient input (often both referred to in the literature as harmful algal blooms, or HABs) or at what nutrient concentration an ecosystem is pushed into a HAB condition (i.e., the threshold value). Local studies may be able to obtain information on such non-linear responses and include it as part of this status measure.

For the chemical pollution component (Halpern *et al.* 2008), we used a combination of modeled input of fertilizer input as a proxy for land-based organic pollution, and impermeable surfaces as a proxy for land-based organic pollution, and shipping and port traffic for ocean based pollution. We were not able to assess specific toxic chemicals at the global scale; however regional case studies often will have data available for the quantities and toxicity of a range of chemicals put into watersheds and coastal waters. We also did not have global data for oil spills and so could not include oil pollution, but in future assessments where such data exist it would be included in chemical pollution as well.

Human-derived pathogens are found in coastal waters primarily from sewage discharge or direct human defecation. Since we did not have access to a global database of in situ measurements of pathogen levels, we used a proxy measure for the status of pathogen pollution, namely the number of people in coastal areas without access to improved sanitation facilities (WHO-UNICEF 2015). The underlying assumption is that locations with a low number of people with access to improved facilities will likely have higher levels of coastal water contamination from human pathogens. To estimate this pathogen intensity, we multiplied the human population within 25 miles of the coast by the percentage of population without access to improved sanitation. This allows countries with low coastal population densities and low access to improved sanitation to score better than high population countries with better access if the absolute number of people without access is lower in the small country.

The status of trash pollution was estimated using globally-available plastic pollution data (Eriksen *et al.* 2014).

#### 5.5.0.1 Current status

The status of this goal, \(x\_{cw}\), was calculated as the geometric mean of the four components, such that:

\[
x\_{cw} = \sqrt [ 4 ]{ a\ast u\ast l\ast d }, (Eq. 5.8)
\]

where \(a\) = 1 - the number of people without access to sanitation, rescaled to the global maximum; \(u\) = 1 – (nutrient input), rescaled at the raster level by the 99.99th quantile value; \(l\) = 1 – (chemical input), rescaled at the raster level by the 99.99th quantile value; and \(d\) = 1 – (marine debris), rescaled at the raster level by the 99.99th quantile value.

We used a geometric mean, as is commonly done for water quality indices (Liou *et al.* 2004), because a very bad score for any one sub-component would pollute the waters sufficiently to make people feel the waters were ‘too dirty’ to enjoy for recreational or aesthetic purposes (e.g., a large oil spill trumps any other measure of pollution).

Although clean waters are relevant and important anywhere in the ocean, coastal waters drive this goal both because the problems of pollution are concentrated there and because people predominantly access and care about clean waters in coastal areas. The nearshore area is what people can see and where beach-going, shoreline fishing, and other activities occur. Furthermore, the data for high seas areas is limited and there is little meaningful regulation or governance over the input of pollution into these areas. We therefore calculate this goal only for the first 3 nm of ocean for each country’s EEZ. We chose 3 nm for several reasons, but found the status results to be relatively insensitive to different distances.

A number of potential components of clean water were not included due to lack of global datasets, including toxic algal blooms, oil spills, turbidity (sediment input), and floating trash. In future applications of the Index where such data are available, they would be included in their appropriate component of clean waters (nutrients, chemicals, and trash, respectively).

#### 5.5.0.2 Trend

Trends in eutrophication, pathogens, and chemical pollution are estimated as described in section 4.3.1. Because only one of the inputs (organic pollution) of the chemical pollution layer includes data over time, the trend only reflects this input. Marine debris trends are estimated using a secondary dataset describing the amount of improperly disposed of plastics within each country (Jambeck *et al.* 2015).

#### 5.5.0.3 Data

*Status and trend*

Chemical pollution trend (cw\_chemical\_trend): Trends in chemical pollution, based on commercial shipping traffic, ports and harbors, land-based pesticide use (organic pollution), and urban runoff (inorganic pollution) within EEZ

Coastal chemical pollution (po\_chemicals\_3nm): Modeled chemical pollution within 3nm of coastline from commercial shipping traffic, ports and harbors, land-based pesticide use (organic pollution), and urban runoff (inorganic pollution)

Coastal nutrient pollution (po\_nutrients\_3nm): Modeled nutrient pollution within EEZ based on fertilizer consumption

Marine plastics (po\_trash): Global marine plastic pollution

Nutrient pollution trend (cw\_nutrient\_trend): Trends in nutrient pollution, based on fertilizer consumption as a proxy for nutrient pollution

Pathogen pollution (po\_pathogens): Percent of population without access to improved sanitation facilities as a proxy for pathogen pollution

Pathogen pollution trend (cw\_pathogen\_trend): Trends in percent of population without access to improved sanitation facilities as a proxy for pathogen pollution

Plastic trash trends (cw\_trash\_trend): Trends in trash estimated using improperly disposed of plastics

*Pressure*

Coastal chemical pollution (po\_chemicals\_3nm): Modeled chemical pollution within 3nm of coastline from commercial shipping traffic, ports and harbors, land-based pesticide use (organic pollution), and urban runoff (inorganic pollution)

Coastal nutrient pollution (po\_nutrients\_3nm): Modeled nutrient pollution within EEZ based on fertilizer consumption

Marine plastics (po\_trash): Global marine plastic pollution

Pathogen pollution (po\_pathogens): Percent of population without access to improved sanitation facilities as a proxy for pathogen pollution

Weakness of governance (ss\_wgi): Inverse of World Governance Indicators (WGI) six combined scores

Weakness of social progress (ss\_spi): Inverse of Social Progress Index scores

*Resilience*

Management of waters to preserve biodiversity (po\_water): Survey responses by country to the Convention on Biological Diversity (CBD) Third National Report: tourism related questions

Social Progress Index (res\_spi): Social Progress Index scores

Strength of governance (wgi\_all): World Governance Indicators (WGI) six combined scores

## 5.6 Food Provision

One of the most fundamental services the ocean provides people is the provision of seafood. From meeting the basic nutritional needs of over half of the world’s population to being sold in high-end sushi restaurants, seafood is an important benefit of healthy oceans. This goal measures the amount of seafood sustainably harvested within an EEZ or region through any means for use primarily in human consumption and thus includes wild-caught commercial fisheries, mariculture, artisanal-scale and recreational fisheries. Importantly, seafood harvest using unsustainable fishing practices or catch levels is penalized, as well as underexploitation of a sustainable resource as the goal aims to maximize the amount of sustainably produced seafood from wild or cultured stocks. Because we do not track where the fish go after being caught or produced, this goal does not aim to measure food security for the population of a given country, but instead measures the food provided from its waters.

The status of the food provision goal is calculated as the mean of the fisheries and mariculture subgoals, weighted by their relative contribution in tonnes to food production for each region.

### 5.6.1 Fisheries (subgoal of food provision)

This model aims to assess the amount of wild-caught seafood that can be sustainably harvested with penalties assigned for both over- and under-harvesting. As such, one must establish a reference point at which harvest is both maximal and sustainable. We assess food provision from wild caught fisheries by estimating population biomass relative to the biomass that can deliver maximum sustainable yield (\(B/B\_{MSY}\)) for each stock. When available, we obtained \(B/B\_{MSY}\) values from the RAM Legacy Stock Assessment Database (Ricard *et al.* 2012), which contains stock assessment information for a portion of global fish stocks. When RAM data were not available, we used data-limited approaches that have been developed to estimate \(B/B\_{MSY}\) values using globally available catch data (Costello *et al.* 2012, 2016; Martell & Froese 2013; Thorson *et al.* 2013; Rosenberg *et al.* 2014). To calculate the status for each region and year, \(B/B\_{MSY}\) values were converted to a stock status score between 0-1 that penalizes both over- and under-harvesting. To obtain the overall status for each region, the stock status scores for all the stocks within a region were averaged using a geometric mean weighted by the average catch (tonnes) of each stock.

**Figure 5.1: Overview of fisheries status calculations.**

#### 5.6.1.1 Current status

*Spatial allocation of catch to regions*

The data we use to calculate \(B/B\_{MSY}\) and the weights used in the geometric mean are from Sea Around Us Project (SAUP) catch reconstruction data (Pauly & Zeller 2015). SAUP uses a spatial allocation method to distribute FAO catches (which are reported at the country scale) to a global grid of one half-degree cell resolution based on the spatial distribution of commercial taxa and the allocation of catch to fleets based on fishing access agreements (Zeller *et al.* 2016).

We aggregate the SAUP catch data in two ways. To get the data needed to weight the stock status scores, we sum the total catch for each taxa within each region’s EEZ to get the total catch in tonnes for each year. To get the data needed to calculate \(B/B\_{MSY}\) values, we sum the total catch for each taxa within each major fishing region (FAO Fisheries and Aquaculture Department 2015) for each year.

*Estimating \(B/B\_{MSY}\)*

When we were unable to obtain \(B/B\_{MSY}\) values from the RAM database for a stock, we calculated them using a data-poor, or catch only model, developed by Martell & Froese (2013), and hereafter referred to as the “catch-MSY” method. The latter was chosen, among other data-limited methods because it was as good, or better, at predicting RAM \(B/B\_{MSY}\) values than other methods based on our initial testing. We compared \(B/B\_{MSY}\) scores from three catch models (catch-MSY, SSCOM, and COMSIR) as well as a variety of ensemble methods (Anderson *et al.* 2017). The catch\_MSY model performed as well or better than the other models at predicting RAM \(B/B\_{MSY}\) values. Furthermore, this method performed well (although not as well as the Random Forest ensemble approach, based on a rank correlation analysis) in analyses using simulated stocks with a broad range of life history traits and different known sources of uncertainty (Anderson *et al.* 2017).

We defined a stock as taxa occurring within a major fishing area, and consequently, we ran the catch-MSY model using yearly catch data aggregated to FAO region from 1950 to the most current year. We chose this definition because many fish populations straddle the boundaries of EEZs. Any aggregation method will be biased in some way, but populations with the largest catches are most often straddling stocks, so a bias in assessments due to erroneous aggregation of catch could occur more often with cosmopolitan species that include small, sedentary (i.e., patchily distributed) populations that are less likely to play a dominant role in a country’s fisheries. The catch-MSY model was applied only to stocks identified to the species level.

The catch-MSY method is based on the same assumptions used in many stock assessment models (Schaefer 1954), namely that the change in a population’s biomass depends on its biomass in the previous year and two population-specific parameters: the carrying capacity (\({K}\)) and rate of population increase (\({r}\)). The method estimates the status of a given population using landings time-series as proxies for biomass removals from the population, and using empirically derived relationships of relative peak to current catch values to estimate depletion at the end of the time series (Martell & Froese 2013). Then, a sampling procedure is used to estimate the distribution of values of \({r}\) and \({K}\) that are compatible with the estimated current depletion levels, and are constrained within the range that maintains viable population abundance and at the same time does not exceed the population’s carrying capacity. In the original formulation of Martell & Froese (2013) the geometric mean \({r}\) and \({K}\) were used to derive an estimate of MSY. Rosenberg et al. (2014) modified this method by producing a biomass time series for each of the viable \({r-K}\) pairs using the surplus production model. The arithmetic mean biomass time series was selected and the current year stock abundance (\({B}\)) relative to the abundance that achieves \({MSY}\) (\(B\_{MSY}\)) produced a measure, \(B/B\_{MSY}\).

A potential issue of the catch-MSY method (when using the default “constrained” prior) is that declining catch is assumed to indicate declining population biomass (resulting in lower \(B/B\_{MSY}\) values) rather than reduced effort or improved management. When declining catch is known to be due to reduced effort and/or improved management this results in artificially low \(B/B\_{MSY}\) values; however, the catch-MSY model can be modified by using a “uniform” prior distribution for the final biomass. However, this adjustment should be considered carefully because the model will assume that all stocks with declining catch are rebuilding (resulting in higher \(B/B\_{MSY}\) values), which is unrealistic. Previously, for the 2015 assessment, we attempted to use the constrained vs. uniform prior for each stock based on the catch weighted fishery management scores of the regions catching the stock. However, recent analyses suggest this method did not improve the ability of the catch-MSY derived \(B/B\_{MSY}\) values to predict RAM values, suggesting we were adding additional complication that did not improve our model. Consequently, all analyses are done using the “constrained” prior.

*Weights for stock status scores*

To get the data needed to weight the stock status scores, we sum the total catch for each taxa within each region’s EEZ to get the total catch in tonnes for each year. We then average each taxa’s catch within each region across all years from 1980 to the most current year’s data. Consequently, for a taxa within a region, the average catch value is the same across all years (only the \(B/B\_{MSY}\) value will vary across years.). This provides an estimate of the mean potential contribution of each species to total food provision, independent of yearly stochastic fluctuations of the population and possible recent declines.

*Goal model calculations*

The status of wild caught fisheries, \(x\_{fis}\), for each reporting region in each year was calculated as the geometric mean of the stock status scores, \(SS\) (derived from \(B/B\_{MSY}\) score for each stock, described below) and weighted by the stock’s relative contribution to overall catch, \(C\), such that:

\[
x\_{fis} = \prod \_{ i=1 }^{ n }{ { SS }\_{ i }^{ (\frac { { C }\_{ i } }{ \sum { { C }\_{ i } } } ) } }, (Eq. 5.9)
\]

where \({i}\) is an individual taxon and \(n\) is the total number of taxa in the reported catch for each region throughout the time-series, and \({C}\) is the average catch, since the first non null record, for each taxon within each region.

We used a geometric weighted mean to account for the portfolio effect of exploiting a diverse suite of resources, such that small stocks that are doing poorly will have a stronger influence on the overall score than they would using an arithmetic weighted mean, even though their \({C}\) contributes relatively little to the overall tonnage of harvested seafood within a given region. The behavior of the geometric mean is such that improving a well-performing stock is not rewarded as much as improving one that is doing poorly. We believe this behavior is desirable because the recovery of stocks in poor condition requires more effort and can have more important effects on the system than making a species that is already abundant even more abundant. In this way, the score is not solely driven by absolute tonnes of fish produced and accounts for preserving the health of a diversity of species.

\(B/B\_{MSY}\) values were used to derive stock status scores, \({SS}\), such that the best score is achieved for stocks at \(B/B\_{MSY} = 1\%\), with a 5% error buffer, and it decreases as the distance of \(B\) from \(B\_{MSY}\) increases, due to under- or over-exploitation (Figure 5.2). For each species reported, within each major fishing area, the stock status score was calculated as:

where, for \(B/B\_{MSY} < 1\) (with a 5% buffer), status declines with direct proportionality to the decline of \(\beta\) with respect to \(B\_{MSY}\), while for \(B/B\_{MSY} > 1\) (with a 5% buffer), status declines at rate \(\alpha\), where \(\alpha = 0.5\), so that as the distance of \(\beta\) from \(B\_{MSY}\) increases, status is penalized by half of that distance. For \(B/B\_{MSY} > 1.05\), \(\beta\) is the minimum score a stock can get, and was set at \(\beta = 0.25\). The \(\alpha\) value ensures that the penalty for under-harvested stocks is half of that for over-harvested stocks (\(\alpha = 1.0\) would assign equal penalty). The \(\beta\) value ensures stocks with \(B/B\_{MSY} > 1.4\) due to, for example, an exceptionally productive year, are not unduly penalized, and also recognizes that it is much easier to improve the goal score when stocks are under-harvested (i.e., increase fishing pressure) than it is when populations are over-harvested and need to be rebuilt. Both parameters \(\alpha\) and \(\beta\) were chosen arbitrarily because there is no established convention for this particular approach. Thus, consistent with previous work (Halpern *et al.* 2012), countries are rewarded for having wild stocks at the biomass that can sustainably deliver the maximum sustainable yield, +/-5% to allow for measurement error, and are penalized for both over- or under-harvesting.

For the 2016 assessment, we did not apply underharvest penalties to the following stocks: Katsuwonus pelamis (FAO region 71), Clupea harengus (FAO region 27), Trachurus capensis (FAO region 47), Sardinella aurita (FAO region 34), Scomberomorus cavalla (FAO region 31). These stocks are fished in multiple regions and the current model formulation ends up penalizing the regions that have the highest proportion of catch of these stocks, which is opposite of what we would like to do. This suggests that our approach to penalizing underharvested stocks could be improved at the model level.

**Figure 5.2: Conversion of \(B/B\_{MSY}\) to stock status, \(SS\) score.**

We needed to gapfill missing status, \(SS\), scores for a large proportion of the catch. Gapfilling was necessary because we could only estimate \(B/B\_{MSY}\) values for taxa identified to the species level. Furthermore, we were unable to estimate \(B/B\_{MSY}\) values for some species due to model non-convergence or too few years of catch data. Missing status scores were gapfilled using the median status scores of the stocks sharing a region and year, the median value was then adjusted using a taxonomic reporting penalty (Table 5.7). For catch not reported to the species level, a penalty was applied for increasingly coarser taxonomic reporting, as this is considered a sign of minimal monitoring and management. We based the penalty on the ISSCAAP convention for taxon codes (http://www.fao.org/fishery/collection/asfis/en), which defines 6 levels of taxonomic aggregation, from 6 (species) to 1 (order or higher). When \({g}<6\), a penalized gapfilled value for status was estimated for the taxa in each region:

**Table 5.7: Penalty applied to gapfilled stock status scores** The penalty is multiplied by the gapfilled stock status score to obtain the final stock status score.

| ISSCAAP taxon code | Description | Penalty (gapfilled score multiplied by value) |
| --- | --- | --- |
| 1 | e.g., Marine fishes not identified, Miscellaneous marine molluscs | 0.1 |
| 2 | Class, Subclass, Subphylum (e.g., Cephalopoda, Holocephali, Crustacea) | 0.25 |
| 3 | Order (e.g., Chimaeriformes, Octopoda) | 0.5 |
| 4 | Family (e.g., Lamnidae, Squillidae) | 0.8 |
| 5 | Genus (e.g., Strongylocentrotus, Scyllarides) | 0.9 |
| 6 | Species | 1 (no penalty) |

*Model limitations*

This model is based on single-species assessments of stock status and thus cannot predict the effect of multi-species interactions. This model adopts \(B=B\_{MSY}\) as a single-species reference point, which by various assessment frameworks is considered very conservative (e.g., Froese *et al.* (2011)), and the fact that the single-species values are aggregated using a geometric mean ensures that some multi-species effects may influence the scores. Nonetheless, a better understanding of the emerging effects of fishing various species at their reference levels would be desirable and will hopefully be possible in the future.

Despite the fact that invertebrates represent a large proportion of global caught biomass, and represent the dominant stocks in many regions, stock assessment approaches for these taxa are poorly developed. The catch-MSY approach was applied to invertebrates even though the model developers only tested it on fish (Martell & Froese 2013). Part of the challenge in broadly testing this approach on organisms other than fish is the lack of a large enough collection of invertebrate assessments to use for validation testing.

This approach captures whether stocks have been historically well managed, but it is worth noting that it does not directly measure current food production.

#### 5.6.1.2 Trend

Trend was calculated as described in section 4.3.1.

#### 5.6.1.3 Data

*Status and trend*

B/Bmsy estimates (fis\_b\_bmsy): The ratio of fish population abundance compared to the abundance required to deliver maximum sustainable yield (RAM data and catch-MSY)

Fishery catch data (fis\_meancatch): Mean commercial catch for each OHI region (averaged across years)

*Pressure*

Chemical pollution (po\_chemicals): Modeled chemical pollution within EEZ from commercial shipping traffic, ports and harbors, land-based pesticide use (organic pollution), and urban runoff (inorganic pollution)

Genetic escapes (sp\_genetic): Introduced mariculture species (Mariculture Sustainability Index) as a proxy for genetic escapes

High bycatch due to artisanal fishing (fp\_art\_hb): The presence of destructive artisanal blast and poison (cyanide) fishing.

High bycatch due to commercial fishing (fp\_com\_hb): Modeled destructive commercial fishing practices by 5 gear types and scaled by Net Primary Productivity

Intertidal habitat destruction (hd\_intertidal): Coastal population density (25 mi from shore) as a proxy for intertidal habitat destruction

Low bycatch due to artisanal fishing (fp\_art\_lb): Extent of artisanal fishing (including: artisanal, subsistence, and recreational catch)

Low bycatch due to commercial fishing (fp\_com\_lb): Modeled destructive commercial fishing practices by 2 gear types and scaled by Net Primary Productivity

Nonindigenous species (sp\_alien): Measure of harmful invasive species

Nutrient pollution (po\_nutrients): Modeled nutrient pollution within 3nm of coastline based on fertilizer consumption

Subtidal hardbottom habitat destruction (hd\_subtidal\_hb): High bycatch artisanal fishing practices (blast fishing) as a proxy for subtidal hard bottom habitat destruction

Subtidal soft bottom habitat destruction (hd\_subtidal\_sb): Demersal destructive commercial fishing practices (i.e., trawling) in soft bottom habitat as a proxy for soft bottom habitat destruction

Weakness of governance (ss\_wgi): Inverse of World Governance Indicators (WGI) six combined scores

Weakness of social progress (ss\_spi): Inverse of Social Progress Index scores

*Resilience*

Artisanal fisheries management effectiveness (fp\_mora\_artisanal): Quality of management of small-scale fishing for artisanal and recreational purposes

Commercial fishing management (fp\_mora): Regulations and management of commerical fishing

EEZ protected marine areas (fishing preservation) (fp\_mpa\_eez): Protected marine areas within EEZ (lasting special places calculation applied to the entire EEZ)

EEZ protected marine areas (habitat preservation) (hd\_mpa\_eez): Protected marine areas within EEZ (lasting special places calculation applied to the entire EEZ)

Management of habitat to protect fisheries biodiversity (fp\_habitat): Survey responses by country to the Convention on Biological Diversity (CBD) Third National Report: habitat related questions

Management of habitat to protect habitat biodiversity (hd\_habitat): Survey responses by country to the Convention on Biological Diversity (CBD) Third National Report: habitat related questions

Measure of ecological integrity (species\_diversity\_eez): Marine species condition (species subgoal status score) as a proxy for ecological integrity

Social Progress Index (res\_spi): Social Progress Index scores

Strength of governance (wgi\_all): World Governance Indicators (WGI) six combined scores

### 5.6.2 Mariculture (subgoal of food provision)

The mariculture subgoal attempts to measure each region’s food production from mariculture relative to its capacity. A basic problem facing assessments of mariculture is the lack of an ecologically- and socially-based reference point for the potential yield of every suitable mariculture species for every type of geographic habitat and location. Determining such reference points for every country at the global scale is a daunting challenge, not only because so much information is lacking, but also because species, genotypes and habitats are likely to change. Consequently, the reference point is based on a comparison of country performance of the harvested tonnes per coastal inhabitant.

#### 5.6.2.1 Current status

The status of the mariculture subgoal, \(x\_{mar}\), was defined as production of strictly marine taxa from both the marine and brackish water FAO categories, excluding aquatic plants such as kelps and seaweeds, which were assumed to contribute predominantly to medicinal and cosmetic uses rather than as a source of food. The data reported by FAO does not always clearly describe whether harvest is derived through mariculture or from land-based facilities. Wherever possible, we excluded species that could not have been harvested from coastal waters, such as freshwater cyclids. Mariculture status was therefore assessed as the current sustainably-harvested yield, \(Y\_{c}\), within each country, such that:

\[
x\_{mar}= \frac {Y\_c}{Y\_{c, ref}}, (Eq. 5.10)
\] where, \(Y\_{c,ref}\) is the \(Y\_c\) value that corresponds to the 95th quantile across all regions and years (including and prior to the year of the assessment year data), and \(Y\_c\) is:

\[
Y\_{c} = \frac { \displaystyle\sum \_{ k=1 }^{ N }{ { Y }\_{ k }{ S }\_{ k,r } } }{ { P }\_{ r } }, (Eq. 5.11)
\]

where, \(Y\_{k}\) is the 4-year moving window average of tonnes of production (United Nations 2016b) for each \({k}\) mariculture species that is currently or at one time cultured within a country, \(S\_{k,r}\) is the sustainability score for each \(k\) mariculture species and region, and \(P\_{r}\) is the population within 25 miles of the region’s coast.

Sustainable harvest data is adjusted for coastal population within a country given the assumption that production depends on the presence of coastal communities that can provide the labor force, infrastructures, and economic demand to support the development and economic viability of mariculture facilities. Thus, two regions with an equal number of coastal inhabitants harvesting an equal tonnage of cultured seafood will score the same, as productivity is commensurate to each region’s socio-economic potential to develop mariculture. Stated another way, mariculture development is assumed to scale proportionally with coastal population, which is a proxy for potential logistic limitations to farm development, e.g., presence of infrastructures, coastal access, and locally available workforce.

The reference point was the \(Y\_c\) value of the region scoring in 95th percentile across all years of data, with all regions scoring above that value given a status score of 1.0.

The sustainability score, \(S\_{k,r}\), for each species in each region is based on the Mariculture Sustainability Index (MSI, (1990)). We used the three sub-indices that directly measured long-term renewability of a given mariculture practice: the wastewater treatment index, the origin of feed index (i.e., fishmeal or other) and the origin of seed (i.e., hatchery or wild caught). These scores are country and species-specific, and we require each species’ yield, \(Y\_{k}\), to have a corresponding sustainability score, \(S\_{k,r}\). However, if a country farms a species that was not assessed by the MSI for that country, but it was assessed in other countries, a global average score is used for that species and country. If a country farms a species that was not assessed at all by the MSI but a species within the same genus was assessed, a global average for the genus was used. Finally, if these scores were not available for the categories above, we used the global average for broad taxonomic grouping (e.g., crustaceans, algae, bivalves, etc.). We are aware that there is some bias associated with using scores derived as averages across countries because they were originally assigned to specific species-country pairs, nevertheless this is preferable to applying a sustainability score solely based on a subset of the species harvested.

#### 5.6.2.2 Trend

Trend was calculated as described in section 4.3.1.

#### 5.6.2.3 Data

*Status and trend*

Inland coastal population (mar\_coastalpopn\_inland25mi): Total coastal population within 25 miles of coast.

Mariculture harvest (mar\_harvest\_tonnes): Tonnes of mariculture harvest

Mariculture sustainability score (mar\_sustainability\_score): Mariculture sustainability based on the Mariculture Sustainability Index (MSI)

*Pressure*

Chemical pollution (po\_chemicals): Modeled chemical pollution within EEZ from commercial shipping traffic, ports and harbors, land-based pesticide use (organic pollution), and urban runoff (inorganic pollution)

Coastal nutrient pollution (po\_nutrients\_3nm): Modeled nutrient pollution within EEZ based on fertilizer consumption

Sea level rise (cc\_slr): Sea level rise pressure

Weakness of governance (ss\_wgi): Inverse of World Governance Indicators (WGI) six combined scores

Weakness of social progress (ss\_spi): Inverse of Social Progress Index scores

*Resilience*

Management of mariculture to preserve biodiversity (g\_mariculture): Survey responses by country to the Convention on Biological Diversity (CBD) Third National Report: mariculture related questions

Management of waters to preserve biodiversity (po\_water): Survey responses by country to the Convention on Biological Diversity (CBD) Third National Report: tourism related questions

Mariculture Sustainability Index (g\_msi\_gov): Mariculture practice assessment criteria from the Mariculture Sustainability Index (MSI)

Social Progress Index (res\_spi): Social Progress Index scores

Strength of governance (wgi\_all): World Governance Indicators (WGI) six combined scores

## 5.7 Livelihoods and economies

Due to discontinued and non-updated source datasets, we have not updated the status of this goal since 2013 (changes across scenario years after 2013 are due to changes in pressures/resilience).

The jobs and revenue produced from marine-related industries are clearly of huge value to many people, even those who do not directly participate in the industries but value community identity, tax revenue, and indirect economic and social impacts of a stable coastal economy.

This goal is composed of two equally important sub-goals, livelihoods and economies, which are assessed across as many marine-related sectors as possible (Table 5.8). Livelihoods includes two equally important sub-components, the number of jobs, which is a proxy for livelihood quantity, and the per capita average annual wages, which is a proxy for job quality. Economies is composed of a single component, revenue. We track the two halves of this goal separately because the number and quality of jobs and the amount of revenue produced are both of considerable interest to stakeholders and governments, and could show very different patterns in some cases (e.g., high revenue sectors do not necessarily provide large employment opportunities). The status of the livelihoods and economies goal is the average of the livelihoods and economies subgoals.

The total value of economic industries cannot be captured fully by measuring only the jobs and revenue generated directly by those industries, since activity in the direct industry stimulates additional jobs and revenue in related industries. For example, the fishing industry provides direct jobs to fishers, indirect jobs to fishing gear manufacturing companies, and induced jobs to the restaurants and movie theaters where those manufacturing employees spend their income. In the case of tourism, data describing total jobs and revenue (direct plus indirect and induced) were available from the primary data source, and so we used that information as the best estimate of total employment and total revenue for that sector. For all other sectors we used sector- and development status-specific multipliers derived from the literature to estimate total job or revenue impacts. We did not apply multiplier values to wages since the cascading effects of earned income are more contentious. We assumed that sector-specific job and revenue multipliers are static and globally consistent, but distinct for developed versus developing countries (when such information was available), because we do not have data to resolve temporal or regional differences (Table 5.9). Countries were classified as developed or developing using the Human Development Index (HDI, UNDP (2010)), with all countries identified as “very high human development” classified as developed and all others as developing. We classified regions not assessed by the HDI by compiling information used to calculate the HDI score (schooling, life expectancy and per capita Gross National Income statistics).

For a job or wage sector to be included in our assessment it needed to report at least two time points and have data for all or most coastal regions (reported separately, not as a single global number). However, a sector did not need to have data for all three measures – jobs, wages, and revenue – as this would have eliminated almost every sector. Consequently, the sectors that comprise each of the three measures differ (Table 5.8) and there is variation across regions in which sectors and measures comprise the status score (because of gaps in datasets and the fact that not all sectors exist in all countries). If a region only had one data layer (a single sector for only one measure), a status score was not calculated for that region and instead, a regional average was applied. We used a weighted average of the region’s UN geopolitical region; revenue values were weighted by each region’s GDP, jobs were weighted by each region’s workforce size, and wages were unweighted.

A number of sectors were not included primarily because sufficient data do not exist. In the future, particularly in finer scale applications, it would be desirable to include these sectors, including (but not limited to) ecotourism (beyond just cetacean watching), sailing/kayaking/boating, surfing/kiteboarding, etc., offshore wind and wave energy, navigation assistance, safety and security, coastal development, scientific research, and restoration and conservation.

**Table 5.8. Livelihoods and economies sectors.** Sectors for which data were available for each component of the livelihoods and economies goal.

| Sector | Jobs data | Wages data | Revenue data |
| --- | --- | --- | --- |
| Tourism | X | X | X |
| Commercial fishing | X | X | X |
| Marine mammal watching | X |  |  |
| Aquarium fishing |  |  | X |
| Wave & renewable energy | X |  | X |
| Mariculture | X |  | X |
| Transportation & shipping |  | X |  |
| Ports & harbors |  | X |  |
| Ship & boatbuilding |  | X |  |

**Table 5.9. Sector multipliers.** Sector-specific multipliers used to calculate total jobs and total revenue created by sector-based employment in developing and developed nations. N/A (not applicable) indicates that total employment or total revenue (direct plus indirect and induced) data were provided by primary data source, eliminating the need for a multiplier value. ND indicates no data available for that sector.

|  | Developed Countries |  | Developing Countries |  |
| --- | --- | --- | --- | --- |
| **Sector** | **Jobs** | **Revenue** | **Jobs** | **Revenue** |
| Tourism | N/A | N/A | N/A | N/A |
| Commercial fishing | 1.582 | 1.568 | 1.582 | 1.568 |
| Marine mammal watching | 1.915 | 1.0 | 1.915 | 1.0 |
| Aquarium fishing | ND | 1.568 | ND | 1.568 |
| Wave & tidal energy | 1.88 | 1.652 | 1.88 | 1.652 |
| Mariculture | 2.7 | 2.377 | 1.973 | 1.59 |

This goal aims to maintain coastal livelihoods and economies (i.e., avoid the loss of, coastal and ocean-dependent jobs and revenues), while also maximizing livelihood quality (relative wages). It does not attempt to capture any aspects of job identity (i.e., the reputation, desirability or other social or cultural perspectives associated with different jobs), although one can examine the component parts that make up this goal to evaluate individual sectors and infer implications for job identity. We make the assumption that all marine-related jobs are equivalent, such that, for example, a fisherman could transition to a job in mariculture or ship-building without affecting the score of this goal. While job identity has social and cultural value, there are not adequate data to track individual workers and assess their job satisfaction on a global scale. We also do not include any measure of petroleum extraction, as we do not consider these practices to be related to the biophysical state of the system and, because they rely on a non-renewable resource, they are inherently unsustainable. Furthermore, because of data constraints, this goal does not provide more credit for sectors or economic activities that are more ecologically sustainable. Future, finer scale applications of the Index may incorporate these key considerations.

Gaps were filled in the adjustment datasets (national GDP and national employment) by first determining the average metric value (e.g., average employment rate) in UN geopolitical regions (United Nations (2013d)) for each year based on all countries in that region for which there were data. Using these regional average time series, we fit nonlinear models to the adjustment data. Using the model fit, we determined the slope between each year. To fill in missing data points in country time series, we applied the slope (percent change in the metric) between the missing year and the following year (or previous year, if necessary). We prioritized filling in backwards (e.g., if a country has data from 2006 and 2008, to fill in 2007, one would use the regional delta between 2008 and 2007), but filled forwards when there were no data for a subsequent year.

### 5.7.1 Economies (subgoal of livelihoods and economies)

This subgoal measures the revenue produced from marine-related industries.

#### 5.7.1.1 Current status

The model to estimate the status of the economies sub-goal, \(x\_{eco}\), is:

\[
x\_{eco} = \frac { \displaystyle\sum \_{ k=1 }^{ N }{ { e }\_{ c,k } } }{ \displaystyle\sum \_{ k=1 }^{ N }{ { e }\_{ r,k } } }, (Eq. 5.12)
\]

where, \(e\) is the total adjusted revenue generated directly and indirectly from sector \(k\), at current, \(c\), and reference, \(r\), time points.

Because there is no absolute global reference point for revenue (i.e., a target number would be completely arbitrary), the economies subgoal uses a moving baseline as the reference point. Reference revenue is calculated as the value in the current year (or most recent year), relative to the value in a recent moving reference period, defined as 5 years prior to the current year. This reflects an implicit goal of maintaining coastal revenue on short time scales, allowing for decadal or generational shifts in what people want and expect. We allowed for a longer or shorter gap between the current and recent years if a 5 year span was not available from the data, but the gap could not be greater than 10 years. Our preferred gap between years was as follows (in order of preference): 5, 6, 4, 7, 3, 8, 2, 9, 1, and 10 years.

Absolute values for \(e\) in the current and reference periods were lumped across all sectors before calculating reference values (even though the current and reference years will not be exactly the same for all sectors), allowing a decrease in one sector to be balanced by an increase in another sector. As such, we do not track the status of individual sectors and instead always focus on the status of all sectors together.

To control for inflation/deflation, we used a standard dollar year. To account for broader economic forces that may affect revenue independent of changes in ocean health (e.g., a global recession), we adjusted revenue based on a country’s GDP (i.e., must keep pace with growth in GDP). The current and reference years used for GDP data were based on the average current year and average reference year across the sector data sources used for revenue.

#### 5.7.1.2 Trend

Trend was calculated as the slope in the individual sector values (not summed sectors) for revenue over the most recent five years (as opposed to the status, which examines changes between two points in time, current versus five years prior to current), corrected by GDP. We calculated the average for revenue by averaging slopes across sectors weighted by the revenue in each sector.

#### 5.7.1.3 Data

*Status and trend*

Adjustment factor for revenue (current) (le\_rev\_cur\_adj\_value): GDP during the current status year of each sector/region (World Bank)

Adjustment factor for revenue (reference) (le\_rev\_ref\_adj\_value): GDP during the reference year (i.e., earliest year of data) of each sector/region (World Bank)

GDP (le\_gdp): Gross Domestic Product (GDP)

Revenue adjustment (GDP) (le\_revenue\_adj): GDP data used to adjust revenue values

Revenue for each sector and year (le\_rev\_sector\_year): Revenue for each sector, region, and year

Revenue, most current value (le\_rev\_cur\_base\_value): Revenue data for the most recent year of data (relative to the assessment year) for each sector/region

Revenue, reference value (le\_rev\_ref\_base\_value): Revenue data for the earliest year of each sector for each sector/region

Sectors in each region (le\_sector\_weight): Proportion of jobs within each sector

*Pressure*

Chemical pollution (po\_chemicals): Modeled chemical pollution within EEZ from commercial shipping traffic, ports and harbors, land-based pesticide use (organic pollution), and urban runoff (inorganic pollution)

Coastal nutrient pollution (po\_nutrients\_3nm): Modeled nutrient pollution within EEZ based on fertilizer consumption

Genetic escapes (sp\_genetic): Introduced mariculture species (Mariculture Sustainability Index) as a proxy for genetic escapes

High bycatch due to artisanal fishing (fp\_art\_hb): The presence of destructive artisanal blast and poison (cyanide) fishing.

High bycatch due to commercial fishing (fp\_com\_hb): Modeled destructive commercial fishing practices by 5 gear types and scaled by Net Primary Productivity

Intertidal habitat destruction (hd\_intertidal): Coastal population density (25 mi from shore) as a proxy for intertidal habitat destruction

Low bycatch due to artisanal fishing (fp\_art\_lb): Extent of artisanal fishing (including: artisanal, subsistence, and recreational catch)

Low bycatch due to commercial fishing (fp\_com\_lb): Modeled destructive commercial fishing practices by 2 gear types and scaled by Net Primary Productivity

Marine plastics (po\_trash): Global marine plastic pollution

Nonindigenous species (sp\_alien): Measure of harmful invasive species

Nutrient pollution (po\_nutrients): Modeled nutrient pollution within 3nm of coastline based on fertilizer consumption

Ocean acidification (cc\_acid): Ocean acidification pressure scaled using biological thresholds

Pathogen pollution (po\_pathogens): Percent of population without access to improved sanitation facilities as a proxy for pathogen pollution

Sea level rise (cc\_slr): Sea level rise pressure

Sea surface temperature (cc\_sst): Sea surface temperature anomalies

Subtidal hardbottom habitat destruction (hd\_subtidal\_hb): High bycatch artisanal fishing practices (blast fishing) as a proxy for subtidal hard bottom habitat destruction

Subtidal soft bottom habitat destruction (hd\_subtidal\_sb): Demersal destructive commercial fishing practices (i.e., trawling) in soft bottom habitat as a proxy for soft bottom habitat destruction

Weakness of governance (ss\_wgi): Inverse of World Governance Indicators (WGI) six combined scores

Weakness of social progress (ss\_spi): Inverse of Social Progress Index scores

*Resilience*

Global Competitiveness Index (GCI) scores (li\_gci): Competitiveness in achieving sustained economic prosperity

Social Progress Index (res\_spi): Social Progress Index scores

Strength of governance (wgi\_all): World Governance Indicators (WGI) six combined scores

### 5.7.2 Livelihoods (subgoal of livelihoods and economies)

This subgoal measures the jobs produced from marine-related industries. Livelihoods includes two equally important sub-components, the number of jobs, which is a proxy for livelihood quantity, and the per capita average annual wages, which is a proxy for job quality.

#### 5.7.2.1 Current status

The status of the livelihoods sub-goal, \(x\_{liv}\), is calculated as:

\[
x\_{liv} = \frac { \frac { \sum \_{ 1 }^{ k }{ { j }\_{ c,k } } }{ \sum \_{ 1 }^{ k }{ { j }\_{ r,k } } } \quad +\quad \frac { \sum \_{ 1 }^{ k }{ { w }\_{ m,k } } }{ \sum \_{ 1 }^{ k }{ { w }\_{ r,k } } } }{ 2 }, (Eq. 5.13)
\]

where \(j\) is the adjusted number of direct and indirect jobs within sector \(k\) within a region and \(w\) is the average PPP-adjusted wages per job within the sector. Jobs are summed across sectors and measured at current, \(c\), and reference, \(r\), time points. Adjusted wage data are averaged across sectors within each region, \(m\), and the reference country, \(r\), with the highest average wages across all sectors.

Because there is no absolute global reference point for jobs (i.e., a target number would be completely arbitrary), this component of the livelihoods subgoal uses a moving baseline as the reference point. Jobs, \(j\), are calculated as a relative value: the value in the current year (or most recent year), \(c\), relative to the value in a recent moving reference period, \(r\), defined as 5 years prior to \(c\). This reflects an implicit goal of maintaining coastal jobs on short time scales, allowing for decadal or generational shifts in what people want and expect. We allowed for a longer or shorter gap between the current and recent years if a 5 year span was not available from the data, but the gap could not be greater than 10 years. Our preferred gap between years was as follows (in order of preference): 5, 6, 4, 7, 3, 8, 2, 9, 1, and 10 years. For wages, \(w\), we assumed the reference value was the highest value observed across all regions.

Absolute values for \(j\) and \(w\) in the current and reference period (jobs) or region (wages) were lumped across all sectors before calculating relative values (even though the current and reference years will not be exactly the same for all sectors), allowing a decrease in one sector to be balanced by an increase in another sector. As such, we do not track the status of individual sectors and instead always focus on the status of all sectors together. For wages, we use the most current data available for each country and each sector, but only use data from 1990 on, assuming that wages are relatively slow to change over time (apart from inflation adjustments, which we control for by using real dollars) and thus can be compared across sectors and countries without controlling for year.

Wages data were divided by the inflation conversion factor so that wage data across years would be comparable in 2010 US dollars (inflation conversion factors were downloaded from http://oregonstate.edu/cla/polisci/sahr/sahr). These data were also multiplied by the purchasing power parity-adjusted per capita GDP (World Bank 2016). To account for broader economic forces that may affect jobs independent of changes in ocean health (e.g., a global recession), we adjusted jobs data by dividing by percent employment for the corresponding year: (1 – percent unemployment) \* total labor force (World Bank 2014a,b). For example, if unemployment increased from the reference to the current period, we would expect the number of marine-related jobs to decrease by a comparable proportion, without causing a lower score for the goal. Therefore, the objective of the goal is actually no loss of jobs and jobs must keep pace with growth in employment rates or sustain losses no greater than national increases in unemployment rates. The current and reference years used for unemployment data were based on the average current year and average reference year across the sector data sources used for number of jobs.

#### 5.7.2.2 Trend

Trend was calculated as the slope in the individual sector values (not summed sectors) for jobs and wages over the most recent five years (as opposed to the status, which examines changes between two points in time, current versus five years prior to current), corrected by national trends in employment rates and average wages. We then calculated the average trend for jobs across all sectors, with the average weighted by the number of jobs in each sector. We calculated the average trend for wages across all sectors. We then averaged the wages and jobs average slopes to get the livelihoods trend.

#### 5.7.2.3 Data

*Status and trend*

Adjusted workforce size (le\_workforcesize\_adj): Total labor force (number of people) from the World Bank

Adjustment factor for jobs (current) (le\_jobs\_cur\_adj\_value): Percent employment during the current status year for each sector/region

Adjustment factor for jobs (reference) (le\_jobs\_ref\_adj\_value): Percent employment during the reference year (i.e., earliest year of data) for each sector/region

Adjustment factor for wages (current) (le\_wage\_cur\_adj\_value): Values are 1, no subsequent adjustments were made to the wage data

Adjustment factor for wages (reference) (le\_wage\_ref\_adj\_value): Values are 1, no subsequent adjustments were made to the wage data

GDP per capita PPP (le\_gdp\_pc\_ppp): Gross domestic product per person at purchasing power parity

Jobs for each sector and year (le\_jobs\_sector\_year): Number of jobs in each sector, region, and year.

Jobs, most current value (le\_jobs\_cur\_base\_value): Number of jobs in each sector and region for the most recent year of data

Jobs, reference value (le\_jobs\_ref\_base\_value): Number of jobs in each sector and region for the reference year (earliest year of data for sector/region) of data

Sectors in each region (le\_sector\_weight): Proportion of jobs within each sector

Total human population (le\_popn): Human population of OHI regions

Unemployment (le\_unemployment): Unemployment data from the World Bank

Wages for each sector and year (le\_wage\_sector\_year): Wages for each sector, region, and year

Wages, current value (le\_wage\_cur\_base\_value): Wage data for the most recent year of data (relative to the assessment year) for each sector/region

Wages, reference value (le\_wage\_ref\_base\_value): Wage data for the earliest year of data for each sector/region

*Pressure*

Chemical pollution (po\_chemicals): Modeled chemical pollution within EEZ from commercial shipping traffic, ports and harbors, land-based pesticide use (organic pollution), and urban runoff (inorganic pollution)

Coastal nutrient pollution (po\_nutrients\_3nm): Modeled nutrient pollution within EEZ based on fertilizer consumption

Genetic escapes (sp\_genetic): Introduced mariculture species (Mariculture Sustainability Index) as a proxy for genetic escapes

High bycatch due to artisanal fishing (fp\_art\_hb): The presence of destructive artisanal blast and poison (cyanide) fishing.

High bycatch due to commercial fishing (fp\_com\_hb): Modeled destructive commercial fishing practices by 5 gear types and scaled by Net Primary Productivity

Intertidal habitat destruction (hd\_intertidal): Coastal population density (25 mi from shore) as a proxy for intertidal habitat destruction

Low bycatch due to artisanal fishing (fp\_art\_lb): Extent of artisanal fishing (including: artisanal, subsistence, and recreational catch)

Low bycatch due to commercial fishing (fp\_com\_lb): Modeled destructive commercial fishing practices by 2 gear types and scaled by Net Primary Productivity

Marine plastics (po\_trash): Global marine plastic pollution

Nonindigenous species (sp\_alien): Measure of harmful invasive species

Nutrient pollution (po\_nutrients): Modeled nutrient pollution within 3nm of coastline based on fertilizer consumption

Pathogen pollution (po\_pathogens): Percent of population without access to improved sanitation facilities as a proxy for pathogen pollution

Sea level rise (cc\_slr): Sea level rise pressure

Subtidal hardbottom habitat destruction (hd\_subtidal\_hb): High bycatch artisanal fishing practices (blast fishing) as a proxy for subtidal hard bottom habitat destruction

Subtidal soft bottom habitat destruction (hd\_subtidal\_sb): Demersal destructive commercial fishing practices (i.e., trawling) in soft bottom habitat as a proxy for soft bottom habitat destruction

Weakness of governance (ss\_wgi): Inverse of World Governance Indicators (WGI) six combined scores

Weakness of social progress (ss\_spi): Inverse of Social Progress Index scores

*Resilience*

Economic diversity (li\_sector\_evenness): Sector evenness based on Shannon’s Diversity Index calculated on the proportion of jobs in each sector as a measure of economic diversity

Global Competitiveness Index (GCI) scores (li\_gci): Competitiveness in achieving sustained economic prosperity

Social Progress Index (res\_spi): Social Progress Index scores

Strength of governance (wgi\_all): World Governance Indicators (WGI) six combined scores

## 5.8 Natural products

In many countries the harvest of non-food natural products is important for local economies and can also be traded internationally. The sustainable harvest of these products is therefore an important component of a healthy ocean. This goal assesses the ability of countries to maximize the sustainable harvest of living marine resources, such as corals, shells, seaweeds, and fish for the aquarium trade. It does not include bioprospecting which focuses on potential (and largely unknowable and potentially infinite) value rather than current realized value, or non-living products such as oil and gas or mining products which by definition are not sustainable. We include six natural product categories: coral, ornamental fish, fish oil, seaweeds and marine plants, shells, and sponges (Table 5.10). We did not have data for other key natural products such as wood from mangroves, and we excluded oils from mammals as they are widely seen as (currently) unsustainably harvested due to low mammal populations.

#### 5.8.0.1 Current status

To determine the total production in tonnes for each product category we summed the products provided in the FAO commodities data (United Nations 2016a) for each category (Table 5.10).

**Table 5.10. Natural product categories**. List of FAO products included in each of the six natural product categories.

| commodity | subcategory |
| --- | --- |
| corals | Coral and the like |
| fish oil | Alaska pollack oil, nei, Anchoveta oil, Capelin oil, Clupeoid oils, nei, Cod liver oil, Fish body oils, nei, Fish liver oils, nei, Gadoid liver oils, nei, Hake liver oil, Halibuts, liver oils, Herring oil, Jack mackerel oil, Menhaden oil, Pilchard oil, Redfish oil, Sardine oil, Shark liver oil, Shark oil, Squid oil, Pelagic fish oils, nei, Gadiformes, oil, nei, Demersal fish oils, nei, Alaska pollock, oil, nei |
| ornamentals | Ornamental saltwater fish, Ornamental fish nei |
| seaweeds | Agar agar in powder, Agar agar in strips, Agar agar nei, Carrageen (Chondrus crispus), Green laver, Hizikia fusiforme (brown algae), Kelp, Kelp meal, Laver, dry, Laver, nei, Other brown algae (laminaria, eisenia/ecklonia), Other edible seaweeds, Other inedible seaweeds, Seaweeds and other algae, unfit for human consumption, nei, Seaweeds and other algae, fit for human consumption, nei, Other red algae, Other seaweeds and aquatic plants and products thereof, Undaria pinnafitida (brown algae) |
| shells | Abalone shells, Miscellaneous corals and shells, Mother of pearl shells, Oyster shells, Sea snail shells, Shells nei, Trochus shells |
| sponges | Natural sponges nei, Natural sponges other than raw, Natural sponges raw |

The status of each natural product category, \(P\_c\), was calculated as:

\[
P\_c = { H }\_{ c }\ast { S }\_{ c } , (Eq. 5.14)
\] where, \(H\_{c}\) is the harvest level for a category relative to its own (buffered) peak reference point and \(S\_{c}\) is the sustainability of that commodity.

For each commodity, we calculated \(H\_{c}\) as the most recent harvest (in metric tons) per region relative to the maximum harvest ever achieved in that region, under the assumption that the maximum achieved at any point in time was likely the maximum possible. This creates a reference point internal to each country. We then established a buffer around this peak catch because we do not know whether it is sustainable. We rescaled these values from 0-1, with any value within 35% of the peak harvest set to 1.0.

Although we do not know actual sustainable levels of harvest, \(S\_{p}\), in each region, we estimated it based on exposure and risk components for each of the natural products commodities:

\[
S\_{c} = 1- average({ E\_c+R\_c }), (Eq. 5.15)
\]

where \({E\_c}\) is the exposure term and \({R\_c}\) is the risk term for each commodity.

The exposure term, \(E\_c\), is the ln-transformed intensity of harvest calculated as tonnes of harvest per km2 of coral and/or rocky reef, depending on the product, relative to the global maximum. We ln transformed the harvest intensity scores because the distribution of values was highly skewed; because we do not know the true threshold of sustainable harvest, nearly all values would be considered highly sustainable without the log transformation.

The risk term, \(R\_c\), is based on whether a commodity has unsustainable harvest practices (i.e., the intensity of cyanide fishing for ornamental fish, and any harvest of corals since they are CITES protected species). Risk for all corals was set as 1 since species in both subclasses and multiple orders of extant corals in class Anthozoa are listed in CITES Appendices II and III (www.cites.org/eng/app/appendices.php). No sponges, algae or marine plants were listed in CITES and thus their risks were set at 0. Shells were also set as 0 since species were not listed individually in the FAO database and only one marine genus (*Tridacnidae* spp.) and one marine species (*Lithophaga lithophaga*) were identified in CITES Appendix II. Risk for ornamental fish was set based on assessments of cyanide or dynamite fishing by Reefs at Risk Revisited (www.wri.org/publication/reefs-at-risk-revisited) under the assumption that most ornamental fishes are harvested from coral reefs.

For the fish oil commodity sustainability was estimated using the fisheries score for the country. It is not possible to identify which of the species fished in the area are used to extract the fish oil. Therefore the estimate is based on all the stocks harvested.

To estimate the status score, \(x\_{np}\), for each region and year we took the weighted average of the individual product scores, \(P\_c\), such that:

\[
x\_{np} = \frac { \displaystyle\sum \_{ c=1 }^{ N }{ { P }\_{ c }\ast { w }\_{ c } } }{ { { N } } }, (Eq. 5.16)
\]

where \({N}\) is the number of product categories, \(c\), that were harvested, and \(w\_{c}\) is the relative contribution of each product to the overall status of the goal. \(w\_c\) was calculated as the ratio of the maximum US dollar value for a product (from the smoothed, gap-filled data) across all years of data for the product, relative to the sum of maximum values for all products harvested in the country.

If a product had a peak value, but was missing a harvest value for that product in a given year, we used \(w\_{c} = 0\) during the aggregation for that year.

There are several important caveats about the natural product status model. First, our approach is supply (export) based. If declining demand for a natural product causes a decline in production, the producing country’s score declines even if it could (sustainably) produce more. Similarly, if a country chose to reduce or halt production of a natural product in order to improve conservation or sustainability, its score will decline. Second, we do not have Maximum Sustainable Yield (MSY) estimates for any of the six natural products evaluated. When such estimates become available in the future they can easily be incorporated. These scenarios may lead to decreases in the score for a region despite maintenance or even improvement of the sustainable harvest of natural products; we instituted the 35% buffer around peak harvest (described above) as a way to help mitigate these potential issues. Finally, our estimate of the sustainability of many of the harvest practices is likely overly optimistic. For example, fishing for ornamental trade often employs unsustainable techniques such as cyanide fishing, but we have few data to inform such an estimate of sustainability in the status calculation for ornamental fish.

This model requires both harvest tonnes and value data. However, because of inconsistencies with how data are reported to FAO, there are many cases where harvest data but no value data are reported, and vice versa. We gapfilled these data because otherwise these mismatches in reporting would result in losing real data. We used a linear regression model to estimate missing tonnes or US dollar values (Frazier *et al.* 2016). For countries that never harvested a product, we assumed they cannot produce it and treat that as a ‘no data’ rather than a zero value. For countries that harvested a product at any point in time, empty values are treated as zeros since the country has the capacity to harvest that product.

#### 5.8.0.2 Trend

Trend was calculated as described in section 4.3.1.

#### 5.8.0.3 Data

*Status and trend*

Areas of blast fishing (np\_blast): Destructive artisanal blast fishing

Areas of poison fishing (np\_cyanide): Destructive artisanal poison (cyanide) fishing

Natural product harvest (np\_harvest\_tonnes): Yield in metric tonnes of six marine commodities (coral, fish oil, seaweed and plants, shells, sponges, ornamental fish)

Relative harvest tonnes (np\_harvest\_tonnes\_relative): Tonnes of harvest of each commodity relative to maximum harvest (with 35% buffer) of the commodity within the region observed across years.

Relative harvest value (np\_harvest\_product\_weight): Value of harvest of each commodity relative to total harvest of six marine commodities (coral, fish oil, seaweed and plants, shells, sponges, ornamental fish) for each region and year

*Pressure*

Chemical pollution (po\_chemicals): Modeled chemical pollution within EEZ from commercial shipping traffic, ports and harbors, land-based pesticide use (organic pollution), and urban runoff (inorganic pollution)

High bycatch due to artisanal fishing (fp\_art\_hb): The presence of destructive artisanal blast and poison (cyanide) fishing.

Intertidal habitat destruction (hd\_intertidal): Coastal population density (25 mi from shore) as a proxy for intertidal habitat destruction

Low bycatch due to artisanal fishing (fp\_art\_lb): Extent of artisanal fishing (including: artisanal, subsistence, and recreational catch)

Low bycatch due to commercial fishing (fp\_com\_lb): Modeled destructive commercial fishing practices by 2 gear types and scaled by Net Primary Productivity

Nonindigenous species (sp\_alien): Measure of harmful invasive species

Nutrient pollution (po\_nutrients): Modeled nutrient pollution within 3nm of coastline based on fertilizer consumption

Ocean acidification (cc\_acid): Ocean acidification pressure scaled using biological thresholds

Sea level rise (cc\_slr): Sea level rise pressure

Sea surface temperature (cc\_sst): Sea surface temperature anomalies

Subtidal hardbottom habitat destruction (hd\_subtidal\_hb): High bycatch artisanal fishing practices (blast fishing) as a proxy for subtidal hard bottom habitat destruction

Subtidal soft bottom habitat destruction (hd\_subtidal\_sb): Demersal destructive commercial fishing practices (i.e., trawling) in soft bottom habitat as a proxy for soft bottom habitat destruction

UV radiation (cc\_uv): Modeled UV radiation

Weakness of governance (ss\_wgi): Inverse of World Governance Indicators (WGI) six combined scores

Weakness of social progress (ss\_spi): Inverse of Social Progress Index scores

*Resilience*

Artisanal fisheries management effectiveness (fp\_mora\_artisanal): Quality of management of small-scale fishing for artisanal and recreational purposes

CITES signatories (g\_cites): Convention on International Trade in Endangered Species of Wild Fauna and Flora (CITES) signatories

Coastal protected marine areas (fishing preservation) (fp\_mpa\_coast): Protected marine areas within 3nm of coastline (lasting special places goal status score)

Coastal protected marine areas (habitat preservation) (hd\_mpa\_coast): Protected marine areas within 3nm of coastline (lasting special places goal status score)

Commercial fishing management (fp\_mora): Regulations and management of commerical fishing

EEZ protected marine areas (fishing preservation) (fp\_mpa\_eez): Protected marine areas within EEZ (lasting special places calculation applied to the entire EEZ)

EEZ protected marine areas (habitat preservation) (hd\_mpa\_eez): Protected marine areas within EEZ (lasting special places calculation applied to the entire EEZ)

Management of habitat to protect fisheries biodiversity (fp\_habitat): Survey responses by country to the Convention on Biological Diversity (CBD) Third National Report: habitat related questions

Management of habitat to protect habitat biodiversity (hd\_habitat): Survey responses by country to the Convention on Biological Diversity (CBD) Third National Report: habitat related questions

Management of waters to preserve biodiversity (po\_water): Survey responses by country to the Convention on Biological Diversity (CBD) Third National Report: tourism related questions

Measure of coastal ecological integrity (species\_diversity\_3nm): Marine species condition (same calculation and data as the species subgoal status score) calculated within 3 nm of shoreline as a proxy for ecological integrity

Measure of ecological integrity (species\_diversity\_eez): Marine species condition (species subgoal status score) as a proxy for ecological integrity

Social Progress Index (res\_spi): Social Progress Index scores

Strength of governance (wgi\_all): World Governance Indicators (WGI) six combined scores

## 5.9 Sense of Place

This goal attempts to capture the aspects of the coastal and marine system that people value as part of their cultural identity. This definition includes people living near the ocean and those who live far from it but still derive a sense of identity or value from knowing particular places or species exist. This goal is calculated using two equally weighted subgoals: iconic species and lasting special places.

### 5.9.1 Iconic species (subgoal of sense of place)

Iconic species are those that are relevant to local cultural identity through their relationship to one or more of the following: 1) traditional activities such as fishing, hunting or commerce; 2) local ethnic or religious practices; 3) existence value; and 4) locally-recognized aesthetic value (e.g., touristic attractions/common subjects for art such as whales). Ultimately, almost any species can be iconic to someone, and so the intent with this goal was to focus on those species widely seen as iconic from a cultural or existence value (rather than a livelihoods or extractive reason). Habitat-forming species were not included, nor were species harvested solely for economic or utilitarian purposes (even though they may be iconic to a sector or individual).

#### 5.9.1.1 Current status

The status of this sub-goal, \(x\_{ico}\), is the average of status scores of the iconic species in each region based on their IUCN Red List threat categories (IUCN 2016a):

\[
x\_{ico} = \frac { \displaystyle\sum\_{ i=EX }^{ LC }{ S\_{i}\times w\_{i} } }{ \displaystyle\sum\_{ i=EX }^{ LC }{ S\_{i} } }, (Eq. 5.17)
\]

where for each IUCN threat category \(i\), \(S\_{i}\) is the number of assessed species and \(w\_{i}\) is the status (Table 5.3) following the methods described by Butchart et al. (2007). This formulation gives partial credit to species that still exist but are in one of the other threat categories. The reference point is to have the risk status of all assessed species as Least Concern (i.e., a goal score = 1.0). Species that have not been assessed or labeled as data deficient are not included in the calculation.

The list of iconic species was drawn from several (data sources, Section 6, IUCN extinction risk), but primarily from the World Wildlife Fund’s global and regional lists for Priority Species (especially important to people for their health, livelihoods, and/or culture) and Flagship Species (‘charismatic’ and/or well-known). Many lists exist for globally important, threatened, endemic, etc. species, but in all cases it is not clear if or to what extent these species represent culturally iconic species. The World Wildlife Fund is the only data source that included cultural reasons for listing iconic species. Although, iconic species vary largely among regions, we include little regional information in our list (i.e., the same list is applied to nearly all regions). Our ultimate goal is to develop a more region-specific iconic species list.

#### 5.9.1.2 Trend

We calculate trend using data the IUCN provides for current and past assessments of species, which we use to create a time series of risk status for each species. Because IUCN assessments are generally infrequent for any given species, we derive the trend as the annual change in risk status for each species across the previous ten years, rather than a five-year window typical of other goals.

#### 5.9.1.3 Data

*Status and trend*

IUCN extinction risk (ico\_spp\_iucn\_status): IUCN extinction risk category for iconic species located within each region

*Pressure*

Coastal chemical pollution (po\_chemicals\_3nm): Modeled chemical pollution within 3nm of coastline from commercial shipping traffic, ports and harbors, land-based pesticide use (organic pollution), and urban runoff (inorganic pollution)

Coastal nutrient pollution (po\_nutrients\_3nm): Modeled nutrient pollution within EEZ based on fertilizer consumption

High bycatch due to artisanal fishing (fp\_art\_hb): The presence of destructive artisanal blast and poison (cyanide) fishing.

High bycatch due to commercial fishing (fp\_com\_hb): Modeled destructive commercial fishing practices by 5 gear types and scaled by Net Primary Productivity

Intertidal habitat destruction (hd\_intertidal): Coastal population density (25 mi from shore) as a proxy for intertidal habitat destruction

Marine plastics (po\_trash): Global marine plastic pollution

Nonindigenous species (sp\_alien): Measure of harmful invasive species

Ocean acidification (cc\_acid): Ocean acidification pressure scaled using biological thresholds

Sea surface temperature (cc\_sst): Sea surface temperature anomalies

Subtidal hardbottom habitat destruction (hd\_subtidal\_hb): High bycatch artisanal fishing practices (blast fishing) as a proxy for subtidal hard bottom habitat destruction

Targeted harvest of cetaceans and marine turtles (fp\_targetharvest): Targeted harvest of cetaceans and marine turtles

Weakness of governance (ss\_wgi): Inverse of World Governance Indicators (WGI) six combined scores

Weakness of social progress (ss\_spi): Inverse of Social Progress Index scores

*Resilience*

Artisanal fisheries management effectiveness (fp\_mora\_artisanal): Quality of management of small-scale fishing for artisanal and recreational purposes

CITES signatories (g\_cites): Convention on International Trade in Endangered Species of Wild Fauna and Flora (CITES) signatories

Commercial fishing management (fp\_mora): Regulations and management of commerical fishing

EEZ protected marine areas (fishing preservation) (fp\_mpa\_eez): Protected marine areas within EEZ (lasting special places calculation applied to the entire EEZ)

EEZ protected marine areas (habitat preservation) (hd\_mpa\_eez): Protected marine areas within EEZ (lasting special places calculation applied to the entire EEZ)

Management of habitat to protect fisheries biodiversity (fp\_habitat): Survey responses by country to the Convention on Biological Diversity (CBD) Third National Report: habitat related questions

Management of habitat to protect habitat biodiversity (hd\_habitat): Survey responses by country to the Convention on Biological Diversity (CBD) Third National Report: habitat related questions

Management of waters to preserve biodiversity (po\_water): Survey responses by country to the Convention on Biological Diversity (CBD) Third National Report: tourism related questions

Measure of ecological integrity (species\_diversity\_eez): Marine species condition (species subgoal status score) as a proxy for ecological integrity

Social Progress Index (res\_spi): Social Progress Index scores

Strength of governance (wgi\_all): World Governance Indicators (WGI) six combined scores

### 5.9.2 Lasting special places (subgoal of sense of place)

The lasting special places sub-goal focuses on geographic locations that hold particular value for aesthetic, spiritual, cultural, recreational or existence reasons (TRC 2004). This sub-goal is particularly hard to quantify. Ideally one would survey every community around the world to determine the top list of special places, and then assess how those locations are faring relative to a desired state (e.g., protected or well managed). The reality is that such lists do not exist. Instead, we assume areas that are protected indicate special places (i.e., the effort to protect them suggests they are important places). Clearly this is an imperfect assumption but in many cases it will be true.

The identification of protected areas does not indicate the proportion of special places in a region that are protected. To solve this problem we make two important assumptions. First, we assume that all countries have roughly the same percentage of their coastal waters and coastline that qualify as lasting special places. In other words, they all have the same reference target (as a percentage of the total area). Second, we assume that the target reference level is 30% of area protected (Hughes 2003).

#### 5.9.2.1 Current status

We calculate the status of this goal as:

\[
x\_{lsp} = \frac { \left( \frac{\%\_{CMPA}}{\%\_{Ref\_{CMPA}}} + \frac {\%\_{CP}}{\%\_{Ref\_{CP}}} \right) }{ 2 }, (Eq. 5.18)
\]

where, \(\%\_{CMPA}\) is the proportion of coastal marine protected area, \(\%\_{CP}\) is the proportion of coastline protected, and \(\%\_{Ref} = 30%\) for both measures.

We focus only on coastal waters (within 3 nautical miles of shore) for marine special places because we assume lasting special places are primarily in coastal areas. For coastlines, we focus only on the first 1-km-wide strip of land as a way to increase the likelihood that the area being protected by terrestrial parks is connected to the marine system in some way.

We use the United Nation’s World Database on Protected Areas (WDPA) to identify protected areas (UNEP-WCMC 2015). The WDPA aggregates several key databases: IUCN’s World Commission on Protected Areas, Global Marine Protected Areas, UNESCO World Heritage Marine sites, National Parks and Nature Reserves, and the United Nations List of Protected Places. In most cases the year of designation is listed for each protected area.

#### 5.9.2.2 Trend

Trend was calculated as described in section 4.3.1.

#### 5.9.2.3 Data

*Status and trend*

Inland area (rgn\_area\_inland1km): Inland area of OHI regions within 1km of shoreline

Inland coastal protected areas (lsp\_prot\_area\_inland1km): Protected areas located 1 km inland

Offshore area (rgn\_area\_offshore3nm): Offshore area of OHI regions within 3nm of shoreline

Offshore coastal protected areas (lsp\_prot\_area\_offshore3nm): Protected areas located 3nm offshore

*Pressure*

Coastal chemical pollution (po\_chemicals\_3nm): Modeled chemical pollution within 3nm of coastline from commercial shipping traffic, ports and harbors, land-based pesticide use (organic pollution), and urban runoff (inorganic pollution)

Coastal nutrient pollution (po\_nutrients\_3nm): Modeled nutrient pollution within EEZ based on fertilizer consumption

Intertidal habitat destruction (hd\_intertidal): Coastal population density (25 mi from shore) as a proxy for intertidal habitat destruction

Marine plastics (po\_trash): Global marine plastic pollution

Nonindigenous species (sp\_alien): Measure of harmful invasive species

Sea level rise (cc\_slr): Sea level rise pressure

Subtidal hardbottom habitat destruction (hd\_subtidal\_hb): High bycatch artisanal fishing practices (blast fishing) as a proxy for subtidal hard bottom habitat destruction

Weakness of governance (ss\_wgi): Inverse of World Governance Indicators (WGI) six combined scores

Weakness of social progress (ss\_spi): Inverse of Social Progress Index scores

*Resilience*

Management of habitat to protect habitat biodiversity (hd\_habitat): Survey responses by country to the Convention on Biological Diversity (CBD) Third National Report: habitat related questions

Management of waters to preserve biodiversity (po\_water): Survey responses by country to the Convention on Biological Diversity (CBD) Third National Report: tourism related questions

Social Progress Index (res\_spi): Social Progress Index scores

Strength of governance (wgi\_all): World Governance Indicators (WGI) six combined scores

## 5.10 Tourism and recreation

The tourism and recreation goal aims to capture the number of people, and the quality of their experience, visiting coastal and marine areas and attractions. Although coastal tourism industries can be important contributors to coastal economies, the tourism and recreation goal is assessed separately from its economic benefits, which are reported in the coastal livelihoods and economies goal. Few non-economic indicators of tourism and recreation exist at the global scale, consequently, we use employment in the tourism sector as a reasonable proxy measure for the total number of people engaged in coastal tourism and recreation activities. Employment within this sector should respond dynamically to the number of people participating in tourist activities, based on the assumption that the number of hotel employees, travel agents and employees of other affiliated professions will increase or decrease with changing tourism demand within different regions.

#### 5.10.0.1 Current status

The model for the status of the tourism & recreation goal, \(x\_{tr}\), is:

\[
x\_{tr} = \frac{ T\_r }{ T\_{95th}}, (Eq. 5.19)
\]

where, \(T\_{95th}\) is the \(T\_r\) value of the region value that corresponds to the 95th quantile, and:

\[
T\_{r} = { E }\times { S }\times{W}, (Eq. 5.20)
\]

where, \(E\) is the proportion of employees directly involved in the travel and tourism industry, \(S\) is sustainability, and \(W\) is a penalty based on travel warnings issued by the US State Department.

Ideally there would be data available specifically for employment, \(E\), in coastal tourism industries, however the best data available at a global scale reports the proportion of the workforce directly employed in tourism, not just coastal jobs (World Travel and Tourism Council, WTTC 2013). Because we do not know how employment patterns vary geographically within sectors for each region, we assume that the proportion employed in the tourism industry is the same in coastal areas as it is away from the coast, and thus \(E\) is the same whether applied solely to coastal areas or to the entire region.

The WTTC data include jobs for both leisure and business that are directly connected to the tourism industry, including accommodation services, food and beverage services, retail trade, transportation services, and cultural, sports and recreational services, but exclude investment industries and suppliers. Unfortunately it was not possible to determine the proportion of jobs affiliated with strictly leisure tourism. However, some (unknown) proportion of business travelers also enjoy the coast for leisure during their visit to coastal areas, such that we assumed all travel and tourism employment was related to tourism and recreation values. Regional applications of the Index can make use of better-resolved data and more direct measures of tourism, as has been done within the US West Coast (Halpern *et al.* 2014), where data for participation in coastal recreational activities across 19 different sectors were available.

Measures of sustainability are data from the World Economic Forum’s (Crotti & Misrahi 2015) Travel Tourism Competitiveness Index (TTCI). This index measures “the set of factors and policies that enable the sustainable development of the Travel & Tourism sector, which in turn, contributes to the development and competitiveness of a country”.

The index is based on 14 pillars that are organized into four subindexes:

- Enabling Environment (general settings necessary for operating in a country)
  - Business Environment
  - Safety and Security
  - Health and Hygiene
  - Human Resources and Labour Market +ICT Readiness
- Travel and Tourism Policy and Enabling Conditions (specific policies or strategic aspects that impact the travel and tourism industry more directly)
  - Prioritization of Travel and Tourism
  - International Openness
  - Price Competitiveness
  - Environmental Sustainability
- Infrastructure (availability and quality of physical infrastructure of each economy)
  - Air Transport Infrastructure
  - Ground and Port Infrastructure
  - Tourist Service Infrastructure
- Natural and Cultural Resources (principal “reasons to travel”)
  - Natural Resources
  - Cultural Resources and Business Travel

The sustainability factor, \(S\) is calculated as the average of the 4 subindexes. Missing sustainability data were gapfilled using per capita GDP (World Bank data with gaps filled using CIA data) based on a linear regression model. For regions without per capita GDP data, remaining missing data were gapfilled using averages of UN geopolitical regions, (United Nations 2013d) with sustainability data.

Penalties, \(W\) were assigned based on travel warnings issued by the US State Department (Table 5.11). If the travel warning was issued for a region within a country, the penalty was multiplied by 0.5.

**Table 5.11. Travel warning penalties** Penalties, \(W\), based on level or US State Department travel warnings.

| Travel warning | Penalty, \(W\) |
| --- | --- |
| inform | 1 |
| risk | 0.75 |
| avoid nonessential | 0.25 |
| avoid all | 0 |
| get out! | 0 |

#### 5.10.0.2 Trend

Trend was calculated as described in section 4.3.1.

#### 5.10.0.3 Data

*Status and trend*

Percent direct employment in tourism (tr\_jobs\_pct\_tourism): Percent direct employment in tourism

Tourism sustainability index (tr\_sustainability): Tourism Competitiveness Index (TTCI)

US State Department travel warnings (tr\_travelwarnings): Countries with US State Department travel warnings

*Pressure*

Coastal chemical pollution (po\_chemicals\_3nm): Modeled chemical pollution within 3nm of coastline from commercial shipping traffic, ports and harbors, land-based pesticide use (organic pollution), and urban runoff (inorganic pollution)

Coastal nutrient pollution (po\_nutrients\_3nm): Modeled nutrient pollution within EEZ based on fertilizer consumption

Marine plastics (po\_trash): Global marine plastic pollution

Pathogen pollution (po\_pathogens): Percent of population without access to improved sanitation facilities as a proxy for pathogen pollution

Sea level rise (cc\_slr): Sea level rise pressure

Weakness of governance (ss\_wgi): Inverse of World Governance Indicators (WGI) six combined scores

Weakness of social progress (ss\_spi): Inverse of Social Progress Index scores

*Resilience*

Management of waters to preserve biodiversity (po\_water): Survey responses by country to the Convention on Biological Diversity (CBD) Third National Report: tourism related questions

Social Progress Index (res\_spi): Social Progress Index scores

Strength of governance (wgi\_all): World Governance Indicators (WGI) six combined scores

# 6 Description of data layers

### 6.0.1 Tables describing data layers (Table 6.1) and sources (Table 6.2)

**Table 6.1. Data layers of 2016 global OHI assessment** A brief overview of all the data layers used to calculate the global OHI. The “Data layer” variable provides links to a full description of the data layer. The “Description” variable provides link/s to the data preparation scripts (when available). See Table 6.2 for a description of the data sources used to create these data layers.

| Layer | Description | Dimension | References | Updates |
| --- | --- | --- | --- | --- |
| Artisanal fisheries opportunity | The opportunity for artisanal and recreational fishing based on the quality of management of the small-scale fishing sector (data prep) | AO | Mora *et al.* (2009) | none |
| Economic need for artisanal fishing | Per capita purchasing power parity (PPP) adjusted gross domestic product (GDP): GDPpcPPP as a proxy for subsistence fishing need (data prep) | AO | World Bank (2016) | additional year, new reference point |
| Chemical pollution trend | Trends in chemical pollution, based on commercial shipping traffic, ports and harbors, land-based pesticide use (organic pollution), and urban runoff (inorganic pollution) within EEZ (data prep) | CW | Halpern *et al.* (2008); Homer *et al.* (2004); United Nations (2016d); Halpern *et al.* (2015a) | additional year: organic (pesticide) data |
| Nutrient pollution trend | Trends in nutrient pollution, based on fertilizer consumption as a proxy for nutrient pollution (data prep) | CW | Halpern *et al.* (2008); United Nations (2016c) | additional year |
| Pathogen pollution trend | Trends in percent of population without access to improved sanitation facilities as a proxy for pathogen pollution (data prep) | CW | WHO-UNICEF (2015) | none |
| Plastic trash trends | Trends in trash estimated using improperly disposed of plastics (data prep) | CW | Jambeck *et al.* (2015) | new dataset |
| Coastal chemical pollution | Modeled chemical pollution within 3nm of coastline from commercial shipping traffic, ports and harbors, land-based pesticide use (organic pollution), and urban runoff (inorganic pollution) (data prep) | CW, pressure | Halpern *et al.* (2008); Homer *et al.* (2004); United Nations (2016d); Halpern *et al.* (2015a) | additional year: organic (pesticide) data |
| Coastal nutrient pollution | Modeled nutrient pollution within EEZ based on fertilizer consumption (data prep) | CW, pressure | Halpern *et al.* (2008); United Nations (2016c) | additional year |
| Pathogen pollution | Percent of population without access to improved sanitation facilities as a proxy for pathogen pollution (data prep) | CW, pressure | WHO-UNICEF (2015) | none |
| Marine plastics | Global marine plastic pollution (data prep) | CW, pressure | Eriksen *et al.* (2014) | none |
| GDP | Gross Domestic Product (GDP) (data prep) | ECO | World Bank (2016) | none |
| Adjustment factor for revenue (current) | GDP during the current status year of each sector/region (World Bank) (data prep) | ECO | United Nations (2013b); United Nations (2013a); United Nations (2013c); O’Connor *et al.* (2009); United Nations (2012); WTTC (2013) | none |
| Revenue, most current value | Revenue data for the most recent year of data (relative to the assessment year) for each sector/region (data prep) | ECO | United Nations (2013b); United Nations (2013a); United Nations (2013c); O’Connor *et al.* (2009); United Nations (2012); WTTC (2013) | none |
| Revenue adjustment (GDP) | GDP data used to adjust revenue values (data prep) | ECO | United Nations (2013b); United Nations (2013a); United Nations (2013c); O’Connor *et al.* (2009); United Nations (2012); WTTC (2013) | none |
| Adjustment factor for revenue (reference) | GDP during the reference year (i.e., earliest year of data) of each sector/region (World Bank) (data prep) | ECO | United Nations (2013b); United Nations (2013a); United Nations (2013c); O’Connor *et al.* (2009); United Nations (2012); WTTC (2013) | none |
| Revenue, reference value | Revenue data for the earliest year of each sector for each sector/region (data prep) | ECO | United Nations (2013b); United Nations (2013a); United Nations (2013c); O’Connor *et al.* (2009); United Nations (2012); WTTC (2013) | none |
| Revenue for each sector and year | Revenue for each sector, region, and year (data prep) | ECO | United Nations (2013b); United Nations (2013a); United Nations (2013c); O’Connor *et al.* (2009); United Nations (2012); WTTC (2013) | none |
| Sectors in each region | Proportion of jobs within each sector (data prep) | ECO, LIV |  | none |
| B/Bmsy estimates | The ratio of fish population abundance compared to the abundance required to deliver maximum sustainable yield (RAM data and catch-MSY) (data prep) | FIS | Pauly & Zeller (2015) | improved source data; new methods |
| Fishery catch data | Mean commercial catch for each OHI region (averaged across years) (data prep) | FIS | Pauly & Zeller (2015) | improved source data |
| Food provision weights | Proportion of wild caught fisheries relative to total food production (e.g., fisheries and mariculture) (data prep) | FP | Pauly & Zeller (2015); United Nations (2016a) | additional year mariculture; improved fishery catch source data |
| Habitat extent | Area of habitats: mangrove, saltmarsh, seagrass, soft bottom, seaice, coral (data prep 1, data prep 2, data prep 3, data prep 4, data prep 5, data prep 6, data prep 7, data prep 8) | HAB, CP, CS | Bruno & Selig (2007); Burke *et al.* (2011); Halpern *et al.* (2008); Schutte *et al.* (2010); Hamilton & Casey (2016); Bridgham *et al.* (2006); Dahl (2011); Eionet (2008); Joint Nature Conservation Committee (2004); New Zealand Ministry for the Environment (2007); Cavalieri *et al.* (1996); Short *et al.* (2011); UNEP-WCMC & Short (2005); Waycott *et al.* (2009) | none |
| Habitat condition | Current condition of habitat relative to historical condition (data prep 1, data prep 2, data prep 3, data prep 4, data prep 5, data prep 6, data prep 7) | HAB, CP, CS | Bruno & Selig (2007); Burke *et al.* (2011); Halpern *et al.* (2008); Schutte *et al.* (2010); Pauly & Zeller (2015); Bridgham *et al.* (2006); Dahl (2011); Eionet (2008); Joint Nature Conservation Committee (2004); New Zealand Ministry for the Environment (2007); Cavalieri *et al.* (1996); Short *et al.* (2011); UNEP-WCMC & Short (2005); Waycott *et al.* (2009) | additional year: sea ice, soft-bottom |
| Habitat condition trend | Estimated change in habitat condition (data prep 1, data prep 2, data prep 3, data prep 4, data prep 5, data prep 6, data prep 7) | HAB, CP, CS | Bruno & Selig (2007); Burke *et al.* (2011); Halpern *et al.* (2008); Schutte *et al.* (2010); Hamilton & Casey (2016); Bridgham *et al.* (2006); Dahl (2011); Eionet (2008); Joint Nature Conservation Committee (2004); New Zealand Ministry for the Environment (2007); Cavalieri *et al.* (1996); Short *et al.* (2011); UNEP-WCMC & Short (2005); Waycott *et al.* (2009) | additional year: sea ice, soft-bottom |
| IUCN extinction risk | IUCN extinction risk category for iconic species located within each region (data prep) | ICO | Halpern *et al.* (2012); IUCN (2016a) | additional year, new methods |
| GDP per capita PPP | Gross domestic product per person at purchasing power parity (data prep) | LIV | World Bank (2016) | none |
| Adjustment factor for jobs (current) | Percent employment during the current status year for each sector/region (data prep) | LIV | United Nations personal communication (2011); O’Connor *et al.* (2009); France (2011); Thorbourne (2011); WTTC (2013) | none |
| Jobs, most current value | Number of jobs in each sector and region for the most recent year of data (data prep) | LIV | United Nations personal communication (2011); O’Connor *et al.* (2009); France (2011); Thorbourne (2011); WTTC (2013) | none |
| Adjustment factor for jobs (reference) | Percent employment during the reference year (i.e., earliest year of data) for each sector/region (data prep) | LIV | United Nations personal communication (2011); O’Connor *et al.* (2009); France (2011); Thorbourne (2011); WTTC (2013) | none |
| Jobs, reference value | Number of jobs in each sector and region for the reference year (earliest year of data for sector/region) of data (data prep) | LIV | United Nations personal communication (2011); O’Connor *et al.* (2009); France (2011); Thorbourne (2011); WTTC (2013) | none |
| Jobs for each sector and year | Number of jobs in each sector, region, and year. (data prep) | LIV | United Nations personal communication (2011); O’Connor *et al.* (2009); France (2011); Thorbourne (2011); WTTC (2013) | none |
| Total human population | Human population of OHI regions (data prep) | LIV | Kaufmann *et al.* (2010) | none |
| Unemployment | Unemployment data from the World Bank (data prep) | LIV | World Bank (2014b) | none |
| Adjustment factor for wages (current) | Values are 1, no subsequent adjustments were made to the wage data (data prep) | LIV | Oostendorp & Freeman (2012) | none |
| Wages, current value | Wage data for the most recent year of data (relative to the assessment year) for each sector/region (data prep) | LIV | Oostendorp & Freeman (2012) | none |
| Adjustment factor for wages (reference) | Values are 1, no subsequent adjustments were made to the wage data (data prep) | LIV | Oostendorp & Freeman (2012) | none |
| Wages, reference value | Wage data for the earliest year of data for each sector/region (data prep) | LIV | Oostendorp & Freeman (2012) | none |
| Wages for each sector and year | Wages for each sector, region, and year (data prep) | LIV | Oostendorp & Freeman (2012) | none |
| Adjusted workforce size | Total labor force (number of people) from the World Bank (data prep) | LIV | World Bank (2014a) | none |
| Inland coastal protected areas | Protected areas located 1 km inland (data prep) | LSP | UNEP (2015); UNEP-WCMC (2015) | additional year |
| Offshore coastal protected areas | Protected areas located 3nm offshore (data prep) | LSP | UNEP (2015); UNEP-WCMC (2015) | additional year |
| Inland area | Inland area of OHI regions within 1km of shoreline (data prep) | LSP | Claus *et al.* (2012); ESRI (2010); Halpern *et al.* (2012); Halpern *et al.* (2015b) | none |
| Offshore area | Offshore area of OHI regions within 3nm of shoreline (data prep) | LSP | Claus *et al.* (2012); ESRI (2010); Halpern *et al.* (2012); Halpern *et al.* (2015b) | none |
| Inland coastal population | Total coastal population within 25 miles of coast. (data prep) | MAR | International Earth Science Information Network (CIESIN) *et al.* (2005); Claus *et al.* (2012); ESRI (2010); Halpern *et al.* (2012); Halpern *et al.* (2015b) | additional year |
| Mariculture harvest | Tonnes of mariculture harvest (data prep) | MAR | United Nations (2016a) | additional year |
| Mariculture sustainability score | Mariculture sustainability based on the Mariculture Sustainability Index (MSI) (data prep) | MAR | Trujillo (2008) | additional year mariculture data (weights), but no updates to species sustainability |
| Areas of blast fishing | Destructive artisanal blast fishing (data prep) | NP | Burke *et al.* (2011) | none |
| Areas of poison fishing | Destructive artisanal poison (cyanide) fishing (data prep) | NP | Burke *et al.* (2011) | none |
| Relative harvest value | Value of harvest of each commodity relative to total harvest of six marine commodities (coral, fish oil, seaweed and plants, shells, sponges, ornamental fish) for each region and year (data prep) | NP | United Nations (2016c) | additional year |
| Natural product harvest | Yield in metric tonnes of six marine commodities (coral, fish oil, seaweed and plants, shells, sponges, ornamental fish) (data prep) | NP | United Nations (2016c) | additional year |
| Relative harvest tonnes | Tonnes of harvest of each commodity relative to maximum harvest (with 35% buffer) of the commodity within the region observed across years. (data prep) | NP | United Nations (2016c) | additional year |
| Ocean acidification | Ocean acidification pressure scaled using biological thresholds (data prep) | pressure | Feely *et al.* (2009) | additional year, new methods |
| Sea level rise | Sea level rise pressure (data prep) | pressure | AVISO (2016) | additional year, new datasource |
| Sea surface temperature | Sea surface temperature anomalies (data prep) | pressure | NOAA (2015) | new reference point |
| UV radiation | Modeled UV radiation (data prep) | pressure | Thaminnen & Arola (2013) | additional year, new methods |
| High bycatch due to artisanal fishing | The presence of destructive artisanal blast and poison (cyanide) fishing. (data prep) | pressure | Burke *et al.* (2011) | none |
| Low bycatch due to artisanal fishing | Extent of artisanal fishing (including: artisanal, subsistence, and recreational catch) (data prep 1, data prep 2) | pressure | Pauly & Zeller (2015) | improved fishery catch source data; new NPP data |
| High bycatch due to commercial fishing | Modeled destructive commercial fishing practices by 5 gear types and scaled by Net Primary Productivity (data prep 1, data prep 2) | pressure | Pauly & Zeller (2015); Halpern *et al.* (2008); Behrenfeld & Falkowski (1997) | improved fishery catch source data; new NPP data |
| Low bycatch due to commercial fishing | Modeled destructive commercial fishing practices by 2 gear types and scaled by Net Primary Productivity (data prep 1, data prep 2) | pressure | Pauly & Zeller (2015); Halpern *et al.* (2008); Behrenfeld & Falkowski (1997) | improved fishery catch source data; new NPP data |
| Targeted harvest of cetaceans and marine turtles | Targeted harvest of cetaceans and marine turtles (data prep) | pressure | United Nations (2016d) | additional year |
| Intertidal habitat destruction | Coastal population density (25 mi from shore) as a proxy for intertidal habitat destruction (data prep) | pressure | International Earth Science Information Network (CIESIN) *et al.* (2005) | additional year |
| Subtidal hardbottom habitat destruction | High bycatch artisanal fishing practices (blast fishing) as a proxy for subtidal hard bottom habitat destruction (data prep) | pressure | Burke *et al.* (2011) | none |
| Subtidal soft bottom habitat destruction | Demersal destructive commercial fishing practices (i.e., trawling) in soft bottom habitat as a proxy for soft bottom habitat destruction (data prep) | pressure | Pauly & Zeller (2015); Halpern *et al.* (2008) | additional year |
| Chemical pollution | Modeled chemical pollution within EEZ from commercial shipping traffic, ports and harbors, land-based pesticide use (organic pollution), and urban runoff (inorganic pollution) (data prep) | pressure | Halpern *et al.* (2008); Homer *et al.* (2004); United Nations (2016d); Halpern *et al.* (2015a) | additional year: organic (pesticide) data |
| Nutrient pollution | Modeled nutrient pollution within 3nm of coastline based on fertilizer consumption (data prep) | pressure | Halpern *et al.* (2008); United Nations (2016c) | additional year |
| Nonindigenous species | Measure of harmful invasive species (data prep) | pressure | Molnar *et al.* (2008) | none |
| Genetic escapes | Introduced mariculture species (Mariculture Sustainability Index) as a proxy for genetic escapes (data prep) | pressure | Trujillo (2008) | additional year mariculture data, but no updates to Mariculture Sustainability Index |
| Weakness of social progress | Inverse of Social Progress Index scores (data prep) | pressure | Porter (2014); Stern *et al.* (2016) | new dataset |
| Weakness of governance | Inverse of World Governance Indicators (WGI) six combined scores (data prep) | pressure | Kaufmann *et al.* (2010) | additional year |
| Management of habitat to protect fisheries biodiversity | Survey responses by country to the Convention on Biological Diversity (CBD) Third National Report: habitat related questions (data prep) | resilience | Convention on Biological Diversity (2005) | none |
| Commercial fishing management | Regulations and management of commerical fishing (data prep) | resilience | Mora *et al.* (2009) | none |
| Artisanal fisheries management effectiveness | Quality of management of small-scale fishing for artisanal and recreational purposes (data prep) | resilience | Mora *et al.* (2009) | none |
| Coastal protected marine areas (fishing preservation) | Protected marine areas within 3nm of coastline (lasting special places goal status score) (data prep) | resilience | UNEP (2015); UNEP-WCMC (2015) | additional year |
| EEZ protected marine areas (fishing preservation) | Protected marine areas within EEZ (lasting special places calculation applied to the entire EEZ) (data prep) | resilience | UNEP (2015); UNEP-WCMC (2015) | additional year |
| CITES signatories | Convention on International Trade in Endangered Species of Wild Fauna and Flora (CITES) signatories (data prep) | resilience | CITES (2015) | none |
| Management of mariculture to preserve biodiversity | Survey responses by country to the Convention on Biological Diversity (CBD) Third National Report: mariculture related questions (data prep) | resilience | Convention on Biological Diversity (2005) | none |
| Mariculture Sustainability Index | Mariculture practice assessment criteria from the Mariculture Sustainability Index (MSI) (data prep) | resilience | Trujillo (2008) | none |
| Management of tourism to preserve biodiversity | Survey responses by country to the Convention on Biological Diversity (CBD) Third National Report: tourism related questions (data prep) | resilience | Convention on Biological Diversity (2005) | none |
| Management of habitat to protect habitat biodiversity | Survey responses by country to the Convention on Biological Diversity (CBD) Third National Report: habitat related questions (data prep) | resilience | Convention on Biological Diversity (2005) | improved estimates of population density |
| Coastal protected marine areas (habitat preservation) | Protected marine areas within 3nm of coastline (lasting special places goal status score) (data prep) | resilience | UNEP (2015); UNEP-WCMC (2015) | additional year |
| EEZ protected marine areas (habitat preservation) | Protected marine areas within EEZ (lasting special places calculation applied to the entire EEZ) (data prep) | resilience | UNEP (2015); UNEP-WCMC (2015) | additional year |
| Global Competitiveness Index (GCI) scores | Competitiveness in achieving sustained economic prosperity (data prep) | resilience | Crotti & Misrahi (2015) | none |
| Economic diversity | Sector evenness based on Shannon’s Diversity Index calculated on the proportion of jobs in each sector as a measure of economic diversity (data prep) | resilience |  | none |
| Management of waters to preserve biodiversity | Survey responses by country to the Convention on Biological Diversity (CBD) Third National Report: tourism related questions (data prep) | resilience | Convention on Biological Diversity (2005) | none |
| Social Progress Index | Social Progress Index scores (data prep) | resilience | Porter (2014); Stern *et al.* (2016) | new dataset |
| Measure of coastal ecological integrity | Marine species condition (same calculation and data as the species subgoal status score) calculated within 3 nm of shoreline as a proxy for ecological integrity (data prep) | resilience | Kaschner *et al.* (2015); IUCN (2016b); IUCN (2016a) | additional year |
| Measure of ecological integrity | Marine species condition (species subgoal status score) as a proxy for ecological integrity (data prep) | resilience | Kaschner *et al.* (2015); IUCN (2016b); IUCN (2016a) | additional year |
| Strength of governance | World Governance Indicators (WGI) six combined scores (data prep) | resilience | Kaufmann *et al.* (2010) | additional year |
| ISO country codes | Lookup table of country codes (based on ISO 3166 country codes) and Ocean Health Index region identifiers (data prep) | spatial |  | none |
| Region areas based on EEZ boundaries | Area of Ocean Health Index regions modified from exclusive economic zones (data prep) | spatial | Claus *et al.* (2012); ESRI (2010); Halpern *et al.* (2012); Halpern *et al.* (2015b) | none |
| UN geopolitical region classifications | UN geopolitical regions (3 levels), often used for gapfilling missing data (data prep) | spatial |  | none |
| OHI regions | Subset of regions that are not deleted or disputed (data prep) | spatial |  | none |
| Regions | Regions by type (eez, subocean, unclaimed) (data prep) | spatial |  | none |
| Average species condition | Species condition based on average of IUCN threat categories (data prep) | SPP | Kaschner *et al.* (2015); IUCN (2016b); IUCN (2016a) | additional year |
| Average species condition trend | Species trends based on average of IUCN population trend data (data prep) | SPP | Kaschner *et al.* (2015); IUCN (2016b); IUCN (2016a) | additional year |
| Percent direct employment in tourism | Percent direct employment in tourism (data prep) | TR | WTTC (2016) | additional year |
| Tourism sustainability index | Tourism Competitiveness Index (TTCI) (data prep) | TR | Crotti & Misrahi (2015) | additional year |
| US State Department travel warnings | Countries with US State Department travel warnings (data prep) | TR | State (2016) | additional year |
| Coastal protection weights | Habitat extent multiplied by habitat protection rank for: coral, mangrove (offshore and inland 1km), saltmarsh, sea ice (shoreline), and seagrass (data prep) | weighting | Tallis *et al.* (2011) | none |
| Carbon storage weights | Habitat extent multiplied by carbon storage capacity for: mangrove (offshore and inland 1km), saltmarsh, and sea ice (data prep) | weighting | Laffoley & Grimsditch (2009) | none |
| Habitat weights | List of habitats in each region (data prep) | weighting |  | none |

**Table 6.2. Data sources used to create data layers for 2016 global OHI assessment** A brief overview of the data sources used to calculate the global OHI.

| Reference | Description | Layers | Years | Resolution | Updated |
| --- | --- | --- | --- | --- | --- |
| Molnar *et al.* (2008) | The number and type of invasive and harmful invasive species in each marine ecoregion | Nonindigenous species | 2008 | Ecoregion (sensu Spalding et al., 2007) | n |
| Burke *et al.* (2011) | Presence of artisanal blast and poison (cyanide) fishing practices | High bycatch due to artisanal fishing, Habitat extent, Habitat condition, Habitat condition trend, Subtidal hardbottom habitat destruction, Areas of blast fishing, Areas of poison fishing | 2009 | 10 km | n |
| Laffoley & Grimsditch (2009) | Carbon sequestration by habitat | Carbon storage weights | 2009 | habitat | n |
| Convention on Biological Diversity (2005) | Convention on Biological Diversity: Data from Third National Report for regulation of alien species, habitat, mariculture, tourism, and water to preserve biodiversity | Management of habitat to protect fisheries biodiversity , Management of mariculture to preserve biodiversity, Management of tourism to preserve biodiversity, Management of habitat to protect habitat biodiversity, Management of waters to preserve biodiversity, Management of nonindigenous species | 2005 | National | n |
| Halpern *et al.* (2008) | Modeled pollution from urban runoff from impervious surfaces | Chemical pollution trend, Nutrient pollution trend, High bycatch due to commercial fishing, Low bycatch due to commercial fishing, Habitat extent, Habitat condition, Habitat condition trend, Subtidal soft bottom habitat destruction, Chemical pollution, Coastal chemical pollution, Nutrient pollution, Coastal nutrient pollution | 2000 | 1 km | n |
| Homer *et al.* (2004) | Modeled pollution from urban runoff from impervious surfaces | Chemical pollution trend, Chemical pollution, Coastal chemical pollution | 2000 | 1 km | n |
| Halpern *et al.* (2008) | Modeled pollution from pesticides | Chemical pollution trend, Nutrient pollution trend, High bycatch due to commercial fishing, Low bycatch due to commercial fishing, Habitat extent, Habitat condition, Habitat condition trend, Subtidal soft bottom habitat destruction, Chemical pollution, Coastal chemical pollution, Nutrient pollution, Coastal nutrient pollution | 1990-2013 | 1 km (FAO data is National) | y |
| United Nations (2016d) | Modeled pollution from pesticides | Chemical pollution trend, Targeted harvest of cetaceans and marine turtles, Chemical pollution, Coastal chemical pollution | 1990-2013 | 1 km (FAO data is National) | y |
| Halpern *et al.* (2008) | Modeled pollution from shipping and ports | Chemical pollution trend, Nutrient pollution trend, High bycatch due to commercial fishing, Low bycatch due to commercial fishing, Habitat extent, Habitat condition, Habitat condition trend, Subtidal soft bottom habitat destruction, Chemical pollution, Coastal chemical pollution, Nutrient pollution, Coastal nutrient pollution | 2003-2011 | 1 km | n |
| Halpern *et al.* (2015a) | Modeled pollution from shipping and ports | Chemical pollution trend, Chemical pollution, Coastal chemical pollution | 2003-2011 | 1 km | n |
| CITES (2015) | Countries that signed the Convention on International Trade in Endangered Species of Wild Fauna and Flora (CITES) | CITES signatories | 2015 | National | n |
| International Earth Science Information Network (CIESIN) *et al.* (2005) | Raster data of human population | Intertidal habitat destruction, Inland coastal population | 2005-2015 | 2.5 arcmin | y |
| Tallis *et al.* (2011) | Ranks of coastal protection provided by habitats | Coastal protection weights | 2011 | habitat | n |
| Bruno & Selig (2007) | Global coral habitat extent and change in condition | Habitat extent, Habitat condition, Habitat condition trend | 2002,1980-2009,2006 | 0.5 km; 1 km; Sites (points) | n? |
| Burke *et al.* (2011) | Global coral habitat extent and change in condition | High bycatch due to artisanal fishing, Habitat extent, Habitat condition, Habitat condition trend, Subtidal hardbottom habitat destruction, Areas of blast fishing, Areas of poison fishing | 2002,1980-2009,2006 | 0.5 km; 1 km; Sites (points) | n? |
| Halpern *et al.* (2008) | Global coral habitat extent and change in condition | Chemical pollution trend, Nutrient pollution trend, High bycatch due to commercial fishing, Low bycatch due to commercial fishing, Habitat extent, Habitat condition, Habitat condition trend, Subtidal soft bottom habitat destruction, Chemical pollution, Coastal chemical pollution, Nutrient pollution, Coastal nutrient pollution | 2002,1980-2009,2006 | 0.5 km; 1 km; Sites (points) | n? |
| Schutte *et al.* (2010) | Global coral habitat extent and change in condition | Habitat extent, Habitat condition, Habitat condition trend | 2002,1980-2009,2006 | 0.5 km; 1 km; Sites (points) | n? |
| United Nations (2016c) | Export tonnes and value (US dollars) and of coral, ornamental fish, fish oil, sponges, shells, and seaweeds and plants | Nutrient pollution trend, Relative harvest value, Natural product harvest, Relative harvest tonnes, Nutrient pollution, Coastal nutrient pollution | 1976-2013 | National | y |
| Pauly & Zeller (2015) | Global fisheries catch statistics in yield per species | B/Bmsy estimates, Fishery catch data, Low bycatch due to artisanal fishing, High bycatch due to commercial fishing, Low bycatch due to commercial fishing, Food provision weights, Habitat condition, Subtidal soft bottom habitat destruction | 1980-2010 | 0.5 degree | y |
| Mora *et al.* (2009) | Management effectiveness of the world’s marine fisheries | Artisanal fisheries opportunity, Commercial fishing management, Artisanal fisheries management effectiveness | 2009 | National | n |
| Mora *et al.* (2009) | Management effectiveness of artisanal fisheries | Artisanal fisheries opportunity, Commercial fishing management, Artisanal fisheries management effectiveness | 2009 | National | n |
| Halpern *et al.* (2008) | Modeled proportion of fisheries catch using different gear types | Chemical pollution trend, Nutrient pollution trend, High bycatch due to commercial fishing, Low bycatch due to commercial fishing, Habitat extent, Habitat condition, Habitat condition trend, Subtidal soft bottom habitat destruction, Chemical pollution, Coastal chemical pollution, Nutrient pollution, Coastal nutrient pollution | 2008 | 0.5 deg | n |
| World Bank (2016) | Per capita purchasing power parity (PPP) adjusted gross domestic product (GDP): GDPpcPPP | Economic need for artisanal fishing, GDP, GDP per capita PPP | 1990-2015 | National | y |
| Crotti & Misrahi (2015) | Composite measure of 12 aspects of economic competitiveness | Global Competitiveness Index (GCI) scores, Tourism sustainability index | 2012-2015 | National | n |
| World Bank (2016) | Gross Domestic Product; Adjustment to all revenue data layers to factor out global economic fluctuations, in 2012 $USD | Economic need for artisanal fishing, GDP, GDP per capita PPP | 1960-2012 | National | n |
| Halpern *et al.* (2012) | WWF Priority and Flagship Species Lists | IUCN extinction risk, Inland coastal population, Region areas based on EEZ boundaries, Inland area, Offshore area | 2011 | Global; National | n |
| Jambeck *et al.* (2015) | Trends in mismanaged plastic waste for 2010 and projected for 2025 as a proxy for trash trends | Plastic trash trends | 2010-2025 (projected) | National | y |
| World Bank (2014a) | Number of people aged 15 and older who could contribute to the production of goods and services | Adjusted workforce size | 1990-2011 | National | n |
| Claus *et al.* (2012) | Land and ocean areas for OHI land and eez regions | Inland coastal population, Region areas based on EEZ boundaries, Inland area, Offshore area | 2013 | 1 km | n |
| ESRI (2010) | Land and ocean areas for OHI land and eez regions | Inland coastal population, Region areas based on EEZ boundaries, Inland area, Offshore area | 2013 | 1 km | n |
| Halpern *et al.* (2012) | Land and ocean areas for OHI land and eez regions | IUCN extinction risk, Inland coastal population, Region areas based on EEZ boundaries, Inland area, Offshore area | 2013 | 1 km | n |
| Halpern *et al.* (2015b) | Land and ocean areas for OHI land and eez regions | Inland coastal population, Region areas based on EEZ boundaries, Inland area, Offshore area | 2013 | 1 km | n |
| Hamilton & Casey (2016) | Global mangrove habitat extent, from remote sensing and assessments | Habitat extent, Habitat condition trend | 2000-2012 | 30m2, summarized to 500m2 | n |
| Trujillo (2008) | Mariculture Sustainability Index (MSI): traceability and code of practice, fishmeal use, waste treatment, and seed and larvae origin indicators | Mariculture Sustainability Index, Mariculture sustainability score, Genetic escapes | 1994-2003 | National | n |
| United Nations (2016a) | Production of finfish and invertebrates | Food provision weights, Mariculture harvest | 1976-2014 | National | y |
| United Nations personal communication (2011) | Global Number of Fishers | Adjustment factor for jobs (current), Jobs, most current value, Adjustment factor for jobs (reference), Jobs, reference value, Jobs for each sector and year | 1990-2008 | National | n |
| United Nations personal communication (2011) | Global Number of Fishers database, adjusted by proportion of a country’s aquaculture production that is focused on marine species | Adjustment factor for jobs (current), Jobs, most current value, Adjustment factor for jobs (reference), Jobs, reference value, Jobs for each sector and year | 1993-2008 | National | n |
| O’Connor *et al.* (2009) | Jobs based on number of whale watchers in a country and a regional average number of whale watchers per employee. Includes all marine mammal watching. | Adjustment factor for jobs (current), Jobs, most current value, Adjustment factor for jobs (reference), Jobs, reference value, Jobs for each sector and year, Adjustment factor for revenue (current), Revenue, most current value, Adjustment factor for revenue (reference), Revenue, reference value, Revenue for each sector and year, Revenue adjustment (GDP) | 1998-2008 | National | n |
| France (2011) | La Rance (France) and Annapolis (Canada) tidal plants employment data | Adjustment factor for jobs (current), Jobs, most current value, Adjustment factor for jobs (reference), Jobs, reference value, Jobs for each sector and year | 2003-2010 | Points (sites) | n |
| Thorbourne (2011) | La Rance (France) and Annapolis (Canada) tidal plants employment data | Adjustment factor for jobs (current), Jobs, most current value, Adjustment factor for jobs (reference), Jobs, reference value, Jobs for each sector and year | 2003-2010 | Points (sites) | n |
| WTTC (2013) | Total contribution of tourism to employment | Adjustment factor for jobs (current), Jobs, most current value, Adjustment factor for jobs (reference), Jobs, reference value, Jobs for each sector and year, Adjustment factor for revenue (current), Revenue, most current value, Adjustment factor for revenue (reference), Revenue, reference value, Revenue for each sector and year, Revenue adjustment (GDP) | 1988-2012 | National | n |
| UNEP-WCMC (2015) | Location and area of marine and terrestrial protected areas | Coastal protected marine areas (fishing preservation), EEZ protected marine areas (fishing preservation), Coastal protected marine areas (habitat preservation), EEZ protected marine areas (habitat preservation), Inland coastal protected areas, Offshore coastal protected areas | 2010-2015 | Shapefile | y |
| UNEP (2015) | Location and area of marine and terrestrial protected areas | Coastal protected marine areas (fishing preservation), EEZ protected marine areas (fishing preservation), Coastal protected marine areas (habitat preservation), EEZ protected marine areas (habitat preservation), Inland coastal protected areas, Offshore coastal protected areas | 2010-2015 | Shapefile | y |
| United Nations (2013b) | Revenue of Aquarium Trade Fishing derived from commodities database | Adjustment factor for revenue (current), Revenue, most current value, Adjustment factor for revenue (reference), Revenue, reference value, Revenue for each sector and year, Revenue adjustment (GDP) | 1984-2009 | National | n |
| United Nations (2013a) | Total revenue from commercial marine fishing | Adjustment factor for revenue (current), Revenue, most current value, Adjustment factor for revenue (reference), Revenue, reference value, Revenue for each sector and year, Revenue adjustment (GDP) | 1997-2007 | National | n |
| United Nations (2013c) | Total revenue from mariculture production of marine species | Adjustment factor for revenue (current), Revenue, most current value, Adjustment factor for revenue (reference), Revenue, reference value, Revenue for each sector and year, Revenue adjustment (GDP) | 1977-2011 | National | n |
| O’Connor *et al.* (2009) | Total revenue from marine mammal watching | Adjustment factor for jobs (current), Jobs, most current value, Adjustment factor for jobs (reference), Jobs, reference value, Jobs for each sector and year, Adjustment factor for revenue (current), Revenue, most current value, Adjustment factor for revenue (reference), Revenue, reference value, Revenue for each sector and year, Revenue adjustment (GDP) | 1998-2008 | National | n |
| United Nations (2012) | Total revenue from marine renewable energy | Adjustment factor for revenue (current), Revenue, most current value, Adjustment factor for revenue (reference), Revenue, reference value, Revenue for each sector and year, Revenue adjustment (GDP) | 1990/2001-2010/2008 | National | n |
| WTTC (2013) | Total tourism revenue by country, adjusted by country’s relative proportion of coastal area | Adjustment factor for jobs (current), Jobs, most current value, Adjustment factor for jobs (reference), Jobs, reference value, Jobs for each sector and year, Adjustment factor for revenue (current), Revenue, most current value, Adjustment factor for revenue (reference), Revenue, reference value, Revenue for each sector and year, Revenue adjustment (GDP) | 1998-2012 | National | n |
| Kaschner *et al.* (2015) | Aquamaps spatial distribution range maps | Measure of coastal ecological integrity, Measure of ecological integrity, Average species condition, Average species condition trend | 2015 | 0.5 deg | y |
| IUCN (2016b) | IUCN spatial distribution | Measure of coastal ecological integrity, Measure of ecological integrity, Average species condition, Average species condition trend | 2016 | Polygons rasterized to 0.5 deg | y |
| IUCN (2016a) | IUCN threat category; sub-population status for iconic species | IUCN extinction risk, Measure of coastal ecological integrity, Measure of ecological integrity, Average species condition, Average species condition trend | 1965-2016 | National | y |
| Oostendorp & Freeman (2012) | Occupations within commercial fishing, ports and harbors, ship and boat building, tourism, and transportation and shipping | Adjustment factor for wages (current), Wages, current value, Adjustment factor for wages (reference), Wages, reference value, Wages for each sector and year | 1989-2008 | National | n |
| World Bank (2014b) | Percent of the labor force unemployed but able to and looking for work | Unemployment | 1990-2011 | National | n |
| Behrenfeld & Falkowski (1997) | Net Primary Productivity | High bycatch due to commercial fishing, Low bycatch due to commercial fishing | 2002-2015 | 0.083 deg | n |
| Halpern *et al.* (2008) | Modeled N input from fertilizer use as a proxy for nutrient pollution | Chemical pollution trend, Nutrient pollution trend, High bycatch due to commercial fishing, Low bycatch due to commercial fishing, Habitat extent, Habitat condition, Habitat condition trend, Subtidal soft bottom habitat destruction, Chemical pollution, Coastal chemical pollution, Nutrient pollution, Coastal nutrient pollution | 1990-2013 | 1 km (FAO data is National) | y |
| United Nations (2016c) | Modeled N input from fertilizer use as a proxy for nutrient pollution | Nutrient pollution trend, Relative harvest value, Natural product harvest, Relative harvest tonnes, Nutrient pollution, Coastal nutrient pollution | 1990-2013 | 1 km (FAO data is National) | y |
| Feely *et al.* (2009) | Change in aragonite saturation state (ASS) levels | Ocean acidification | 2005-2015 | 1 deg | y |
| Halpern *et al.* (2008) | Global rocky reef habitat extent | Chemical pollution trend, Nutrient pollution trend, High bycatch due to commercial fishing, Low bycatch due to commercial fishing, Habitat extent, Habitat condition, Habitat condition trend, Subtidal soft bottom habitat destruction, Chemical pollution, Coastal chemical pollution, Nutrient pollution, Coastal nutrient pollution | 2005 | 2 arcmin; Points | n |
| Bridgham *et al.* (2006) | Global salt marsh habitat extent and condition | Habitat extent, Habitat condition, Habitat condition trend | 1975-2007 | National | n |
| Dahl (2011) | Global salt marsh habitat extent and condition | Habitat extent, Habitat condition, Habitat condition trend | 1975-2007 | National | n |
| Eionet (2008) | Global salt marsh habitat extent and condition | Habitat extent, Habitat condition, Habitat condition trend | 1975-2007 | National | n |
| Joint Nature Conservation Committee (2004) | Global salt marsh habitat extent and condition | Habitat extent, Habitat condition, Habitat condition trend | 1975-2007 | National | n |
| New Zealand Ministry for the Environment (2007) | Global salt marsh habitat extent and condition | Habitat extent, Habitat condition, Habitat condition trend | 1975-2007 | National | n |
| WHO-UNICEF (2015) | Percent population without access to improved sanitation facilities | Pathogen pollution trend, Pathogen pollution | 1990-2015 | National | n |
| Cavalieri *et al.* (1996) | Sea ice change in extent, both edge and shoreline metrics | Habitat extent, Habitat condition, Habitat condition trend | 1979-2015 | 25 km | y |
| AVISO (2016) | Net change in sea level during the time series | Sea level rise | 1993-2015 | 0.25 deg | y |
| NOAA (2015) | Sea surface temperature anomalies | Sea surface temperature | 1982-2012 | 4 km | n |
| Short *et al.* (2011) | Global seagrass habitat extent and change in condition | Habitat extent, Habitat condition, Habitat condition trend | 1975-2010 | 1 km, National | n |
| UNEP-WCMC & Short (2005) | Global seagrass habitat extent and change in condition | Habitat extent, Habitat condition, Habitat condition trend | 1975-2010 | 1 km, National | n |
| Waycott *et al.* (2009) | Global seagrass habitat extent and change in condition | Habitat extent, Habitat condition, Habitat condition trend | 1975-2010 | 1 km, National | n |
| Porter (2014) | Index measuring quality of life indicators | Social Progress Index, Weakness of social progress | 2016 | National | y |
| Stern *et al.* (2016) | Index measuring quality of life indicators | Social Progress Index, Weakness of social progress | 2016 | National | y |
| Halpern *et al.* (2008) | Global soft-bottom subtidal habitat extent | Chemical pollution trend, Nutrient pollution trend, High bycatch due to commercial fishing, Low bycatch due to commercial fishing, Habitat extent, Habitat condition, Habitat condition trend, Subtidal soft bottom habitat destruction, Chemical pollution, Coastal chemical pollution, Nutrient pollution, Coastal nutrient pollution | 2001-2005 | 0.5 deg | n |
| United Nations (2016d) | Catch statistics for cetaceans and marine turtles | Chemical pollution trend, Targeted harvest of cetaceans and marine turtles, Chemical pollution, Coastal chemical pollution | 1950-2014 | National | y |
| Kaufmann *et al.* (2010) | Census populations for 221 countries | Total human population, Weakness of governance, Strength of governance | 1990-2012 | National | n |
| Eriksen *et al.* (2014) | Plastic trash pollution in ocean | Marine plastics | 2014 | 0.2 deg | n |
| Crotti & Misrahi (2015) | Sustainability of the travel and tourism industry | Global Competitiveness Index (GCI) scores, Tourism sustainability index | 2015 | National | n |
| WTTC (2016) | Employment directly linked to travel and tourism sectors (hotels, transportation, services) | Percent direct employment in tourism | 1988-2015 | National | y |
| State (2016) | Travel warnings issued by the US State Department | US State Department travel warnings | 2015-2016 | National | y |
| Thaminnen & Arola (2013) | Anomalies in intensity of ultraviolet (UV) radiation | UV radiation | 2015 | 1 deg | y |
| Kaufmann *et al.* (2010) | Accountability, Political Stability and Absence of Violence, Government Effectiveness, Regulatory Quality, Rule of Law, and Control of Corruption | Total human population, Weakness of governance, Strength of governance | 1996-2014 | National | y? |

## 6.1 Adjusted workforce size

#### le\_workforcesize\_adj

Data for total labor force (number of people) was obtained from the World Bank http://data.worldbank.org/indicator/SL.TLF.TOTL.IN. Total labor force is defined as those 15 years old and older who can supply labor for the production of goods and services – and includes those employed and unemployed, as well as those in the armed forces, and generally excludes homemakers and other unpaid caregivers and workers in the informal sector. For the three EEZs that fall within the China region (China, Macau, and Hong Kong), we combined the values by summing.

#### Units

number of jobs

## 6.2 Adjustment factor for jobs (current)

#### le\_jobs\_cur\_adj\_value

See **Jobs for each sector and year** layer for more information about data and methods.

Percent employment during the current status year for each sector/region, calculated as (1 - percent unemployment)\*total labor force (World Bank). Jobs data for the livelihoods subgoal were adjusted by dividing by the percent employment in the corresponding year.

#### Units

value

## 6.3 Adjustment factor for jobs (reference)

#### le\_jobs\_ref\_adj\_value

See **Jobs for each sector and year** layer for more information about data and methods.

Percent employment during the reference year (i.e., earliest year of data) for each sector/region, calculated as (1 - percent unemployment)\*total labor force (World Bank). Jobs data for the livelihoods subgoal was adjusted by dividing by the percent employment of the corresponding year.

#### Units

value

## 6.4 Adjustment factor for revenue (current)

#### le\_rev\_cur\_adj\_value

See **Revenue for each sector and year** layer for more information about data and methods.

GDP during the current status year of each sector/region (World Bank). Revenue values used in the economies subgoal were adjusted by dividing by the GDP of the corresponding year.

#### Units

value

## 6.5 Adjustment factor for revenue (reference)

#### le\_rev\_ref\_adj\_value

See **Revenue for each sector and year** layer for more information about data and methods.

GDP during the reference year (i.e., earliest year of data) of each sector/region (World Bank). Revenue values used in the economies subgoal were adjusted by dividing by GDP in the corresponding year.

#### Units

value

## 6.6 Adjustment factor for wages (current)

#### le\_wage\_cur\_adj\_value

See **Wages for each sector and year** layer for information about data and methods.

The adjustment for each sector/region is 1, all adjustments to wage data used in the livelihoods subgoal were made directly to the wage data layers.

#### Units

value

## 6.7 Adjustment factor for wages (reference)

#### le\_wage\_ref\_adj\_value

See **Wages for each sector and year** layer for information about data and methods.

The adjustment for each sector/region is 1, all adjustments to wage data used in the livelihoods subgoal were made directly to the wage data layers.

#### Units

value

## 6.8 Areas of blast fishing

#### np\_blast

See **High bycatch due to artisanal fishing** for description of data and methods.

Data describes the relative prevalence of blast fishing (Reefs at Risk Revisited, Burke et al. 2007).

#### Units

score

## 6.9 Areas of poison fishing

#### np\_cyanide

See **High bycatch due to artisanal fishing** for description of data and methods.

Data describes the relative prevalence of poison (cyanide) fishing (Reefs at Risk Revisited, Burke et al. 2007).

#### Units

score

## 6.10 Artisanal fisheries management effectiveness

#### fp\_mora\_artisanal

*Resilience*

*Category: ecological/regulatory*

*Subcategory: fishing*

See **Artisanal fisheries opportunity** data layer for information about data and methods.

#### Units

scaled 0-1

## 6.11 Artisanal fisheries opportunity

#### ao\_access

This layer represents the opportunity for artisanal and recreational fishing in each country based on the quality of management of the small-scale fishing sector. Global data were extracted from Mora et al. (2009), Figure S4. Figure S4 is based on two expert opinion survey questions related to artisanal and recreational fishing (classified as small-scale fishing; presented in Table S5). Overall scores for small-scale fisheries management for each country are based on a scale of 0 to 100, with higher scores representing better management. These values were then rescaled (using a maximum value of 100 and minimum value of 0) to range between 0 and 1 for each OHI region.

Questions from Mora et al. that were used to evaluate access to artisanal scale fishing:

If recreational fishing exists to any extent, which of the following apply?

- Are recreational fishermen required to have a fishing license? Y/N
- Are there regulations to the size of fish caught? Y/N
- Are there regulations to the number of fish caught? Y/N
- Are there regulations to the number of fishermen allowed to fish? Y/N
- Are there statistics being collected for this sort of fishing? Y/N

If artisanal fishing exists to any extent, which of the following apply?

- Are there regulations to the size of fish caught? Y/N
- Are there regulations to the number of fish caught? Y/N
- Are there regulations to the number of fishermen allowed to fish? Y/N
- Are there statistics being collected for this sort of fishing? Y/N

#### Units

scaled 0-1

## 6.12 Average species condition

#### spp\_status

See Species goal for calculations.

This value reflects the average condition of species (based on risk status from the IUCN Red List of Threatened Species, http://www.iucnredlist.org/) located within the eez of each region based on species range maps from IUCN (shapefiles) and Aquamaps (http://www.aquamaps.org/, half degree resolution rasters).

#### Units

status score

## 6.13 Average species condition trend

#### spp\_trend

See Species goal for calculations.

This value reflects the average trend of species (based on trend status from the IUCN Red List of Threatened Species, http://www.iucnredlist.org/) located within the eez of each region based on the species range maps from IUCN (shapefiles) and Aquamaps (http://www.aquamaps.org/, half degree resolution rasters) range maps.

#### Units

trend score

## 6.14 B/Bmsy estimates

#### fis\_b\_bmsy

Status of global fish stocks based on B/Bmsy values (the ratio of population biomass compared to the biomass required to deliver maximum sustainable yield). We preferentially used B/Bmsy estimates from formal stock assessments from the  
RAM Legacy Database http://ramlegacy.org/. When these data are unavailable, we use the data-limited catch-MSY model (Martell & Frowese 2012) to estimate B/Bmsy values using yearly fish catch reconstruction data from the Sea Around Us Project (SAUP, Zeller and Pauly 2007). SAUP provides catch data (tons per cell) for individual taxa (identified from the species level to “Miscellaneous not identified”) at 0.5 degree resolution. For the catch-MSY model, we defined a stock as a species caught within an FAO major fishing area (www.fao.org/fishery/area/search/en), which eliminates all the taxa not identified to species level. This approach assumes that stocks are defined by FAO region, which we know is often not true because multiple stocks of the same species can exist within an FAO region and some stocks cover multiple FAO regions. However, this assumption is necessary without range maps of stocks. The catch data were summed for each species/FAO region/year, and the catch-MSY model was applied to each stock to estimate B/Bmsy.

#### Units

B/Bmsy

## 6.15 Carbon storage weights

#### element\_wts\_cs\_km2\_x\_storage

This layer describes the relative value of the habitats in each region to carbon storage, and is calculated by multiplying the habitat extent (km2) in each region by the amount of carbon the habitat sequesters (Laffoley & Grimsditch 2009). These data are called internally by ohicore functions (see: conf/config.R to see how these data are specified) to weight the data used to calculate pressure and resilience values.

**Table 6.3. Carbon storage weights**

| Habitat | Carbon storage |
| --- | --- |
| Mangrove | 139 |
| Saltmarsh | 210 |
| Seagrass | 83 |

#### Units

extent\*storage

## 6.16 Chemical pollution

#### po\_chemicals

*Pressure*

*Category: ecological*

*Subcategory: pollution*

This pressure layer is calculated using modeled data for land-based organic pollution (pesticide data), land-based inorganic pollution (using impermeable surfaces as a proxy), and ocean pollution (shipping and ports). These global data are provided at ~1km resolution, with raster values scaled from 0-1 (Halpern et al. 2008, 2015). To obtain the final pressure values, the three raster layers were summed (with cell values capped at 1).

#### 6.16.0.1 Land-based organic pollution

Data were calculated using modeled plumes of land-based pesticide pollution that provide intensity of pollution at 1km2 resolution (Halpern et al. 2008).

Organic pollution was estimated from FAO data on annual country-level pesticide use (http://faostat3.fao.org/faostat-gateway/go/to/browse/R/\*/E), measured in metric tons of active ingredients. FAO uses survey methods to measure quantities of pesticides applied to crops and seeds in the agriculture sector, including insecticides, mineral oils, herbicides, fungicides, seed treatments insecticides, seed treatments fungicides, plant growth regulators and rodenticides. Missing values were estimated by regression between fertilizer and pesticides when possible, and when not possible with agricultural GDP as a proxy. Data were summed across all pesticide compounds and reported in metric tons. Upon inspection the data included multiple 0 values that are most likely data gaps in the time-series, so they were treated as such and replaced with NA. In addition, regions with only 1 data point and regions where the most recent data point was prior to 2005 were excluded. Uninhabited countries were assumed to have no pesticide use and thus excluded.

Region-level pollution values were then dasymetrically distributed over a region’s landscape using global landcover data from 2009, derived from the MODIS satellite data at ~500m resolution. These values were then aggregated by ~140,000 global basins, and diffusive plumes were modeled from each basin’s pourpoint. The final non-zero plumes (about ~76,000) were aggregated into ~1km Mollweide (wgs84) projection rasters to produce a single plume-aggregated pollution raster.

These raw values were then ln(X+1) transformed and normalized to 0-1 by dividing by the 99.99th quantile of raster values across all years. The zonal mean was then calculated for each region.

#### 6.16.0.2 Land-based inorganic pollution

These data are from Halpern et al. (2008, 2015), and available from Knowledge Network for Biocomplexity (KNB, https://knb.ecoinformatics.org/#view/doi:10.5063/F19021PC, rescaled\_2013\_inorganic\_mol). Non-point source inorganic pollution was modeled with global 1 km2 impervious surface area data http://www.ngdc.noaa.gov/dmsp/ under the assumption that most of this pollution comes from urban runoff. These data will not capture point-sources of pollution or nonpoint sources where paved roads do not exist (e.g., select places in developing countries). Values were aggregated to the watershed and distributed to the pour point (i.e., stream and river mouths) for the watershed with raster statistics (i.e., aggregation by watershed).

#### 6.16.0.3 Ocean pollution (shipping lanes, ports)

These data are from Halpern et al. (2015), and available from the Knowledge Network for Biocomplexity (KNB, https://knb.ecoinformatics.org/#view/doi:10.5063/F1DR2SDD, rescaled\_2013\_one\_ocean\_pollution\_mol). Ocean-based pollution combines commercial shipping traffic data and port data.

Shipping data was obtained from two sources: (1) Over the past 20 years, 10-20% of the vessel fleet has voluntarily participated in collecting meteorological data for the open ocean, which includes location at the time of measurement, as part of the Volunteer Observing System (VOS). (2) In order to improve maritime safety, in 2002 the International Maritime Organization SOLAS agreement required all vessels over 300 gross tonnage (GT) and vessels carrying passengers to equip Automatic Identification System (AIS) transceivers, which use the Global Positioning System (GPS) to precisely locate vessels.

Port data was based on the volume (measured in tonnes) of goods transported through commercial ports as a proxy measure of port traffic. Total cargo volume data by port was collected from regional and national statistical organizations, and from published port rankings.

#### Units

scaled 0-1

## 6.17 Chemical pollution trend

#### cw\_chemical\_trend

See description for **Chemical pollution** layer.

The inverse of the pressure data (1 - Coastal chemical pollution) was used to estimate chemical trends for the clean water goal. The proportional yearly change in chemical pressure values were estimated using a linear regression model of the most recent five years of data (i.e., slope divided by value from the earliest year included in the regression model). The slope was then multiplied by five to get the predicted change in 5 years.

The only layer with yearly data was land-based organic pollution (pesticide data). The land-based inorganic pollution (using impermeable surfaces as a proxy) and ocean pollution (shipping and ports) remained the same across years.

#### Units

trend

## 6.18 CITES signatories

#### g\_cites

*Resilience*

*Category: ecological/regulatory*

*Subcategory: goal*

Contracting parties to the Convention on International Trade in Endangered Species of Wild Fauna and Flora (CITES, http://www.cites.org/eng/ disc/parties/alphabet.php). The Convention is an international agreement between governments that aims to ensure that any international trade in plants and animals “does not threaten their survival.” All countries party to the Convention are given full credit for membership; those countries that are not contracting parties are given no credit (score = 0).

#### Units

0 or 1

## 6.19 Coastal chemical pollution

#### po\_chemicals\_3nm

*Pressure*

*Category: ecological*

*Subcategory: pollution*

See description for **Chemical pollution**.

Methods follow those described for the **Chemical pollution** layer. However, the rescaled data were clipped to include only pixels within 3nm offshore, and the zonal mean for each region was calculated using this subset of data.

For the clean waters goal calculations, the inverse of the pressure values is used (1 minus chemical pressure).

#### Units

scaled 0-1

## 6.20 Coastal nutrient pollution

#### po\_nutrients\_3nm

*Pressure*

*Category: ecological*

*Subcategory: pollution*

See description for **Nutrient pollution**.

Methods follow those described for the **Nutrient pollution** layer. However, the rescaled data were clipped to include only pixels within 3nm offshore, and the zonal mean for each region was calculated using this subset of data.

For the clean waters goal calculations, the inverse of the pressure values is used (1 minus nutrient pressure).

#### Units

scaled 0-1

## 6.21 Coastal protected marine areas (fishing preservation)

#### fp\_mpa\_coast

*Resilience*

*Category: ecological/regulatory*

*Subcategory: fishing*

These data are calculated using the lasting special places status subgoal model using total marine protected area (km2) within 3 nm offshore (see **Offshore coastal protected areas** layer for information about the data). Following the lasting special places model, a reference point of 30% is used, such that any region with 30%, or more, protected area receives a score of 1.

#### Units

scaled 0-1

## 6.22 Coastal protected marine areas (habitat preservation)

#### hd\_mpa\_coast

*Resilience*

*Category: ecological/regulatory*

*Subcategory: habitat*

See **Coastal protected marine areas (fishing preservation)** for information about this layer.

#### Units

scaled 0-1

## 6.23 Coastal protection weights

#### element\_wts\_cp\_km2\_x\_protection

This layer describes the relative value of the habitats in each region to coastal protection, and is calculated by multiplying the habitat extent (km2) in each region by the habitat protection rank. These data are called internally by ohicore functions (see: conf/config.R to see how these data are specified) to weight the data used to calculate pressure and resilience values.

**Table 6.4. Coastal protection ranks**

| Habitat | Protection rank |
| --- | --- |
| Coral | 4 |
| Mangrove | 4 |
| Seaice (shoreline) | 4 |
| Saltmarsh | 3 |
| Seagrass | 1 |

#### Units

extent\*rank

## 6.24 Commercial fishing management

#### fp\_mora

*Resilience*

*Category: ecological/regulatory*

*Subcategory: fishing*

These data describe the current effectiveness of fisheries management regimes along 6 axes (Mora et al. 2009): scientific robustness, policy transparency, implementation capacity, subsidies, fishing effort, and foreign fishing. All countries with coastal areas were assessed through a combination of surveys, empirical data and enquiries to fisheries experts. For each OHI reporting region, scores for each category were rescaled between 0 and 1 using the maximum possible value for each category and then the average score of all 6 categories was used as the overall fisheries management effectiveness score.

#### Units

scaled 0-1

## 6.25 Economic diversity

#### li\_sector\_evenness

*Resilience*

*Category: social*

Sector evenness was measured using Shannon’s Diversity Index, a common measure of ecological and economic diversity that has been applied previously to economic sectors (Attaran 1986). The Diversity Index is computed as \({ H }^{ ' }/{ H }\_{ max }\) where:

\[
{ H }^{ ' } = \sum \_{ i }^{ z }{ { f }\_{ i }\ast } \ln { { (f }\_{ i }) }, (Eq. 6.1)
\]

and Z is the total number of sectors, fi is the frequency of the ith sector (the probability that any given job belongs to the sector), and \(H\_{max} = \ln { Z }\).

#### Units

scaled

## 6.26 Economic need for artisanal fishing

#### ao\_need

The need for artisanal fishing opportunities is measured as purchasing power parity adjusted per capita gross domestic product (ppppcgdp) in “constant” USD (World Bank, http://data.worldbank.org/indicator/NY.GDP.PCAP.PP.KD). The World Bank defines gdp as the gross value of all resident producers in the economy plus product taxes and minus and subsidies not included in the value of the products. The gdp is adjusted by population size to get per capita output and by purchasing power parity (ppp) to account for the difference in exchange rates between countries. Values for ppppcgdp were rescaled to values between zero and one by taking the natural log of the values and dividing by the 99th quantile value across all years/regions (2005 to most recent year).

When a region is missing some years of data, a within region linear model is used to estimate the missing values.

This is actually a measure of prosperity, but it is converted to need in the artisanal opportunities goal model (1 minus ppppcgdp).

#### Units

scaled 0-1

## 6.27 EEZ protected marine areas (fishing preservation)

#### fp\_mpa\_eez

*Resilience*

*Category: ecological/regulatory*

*Subcategory: fishing*

These data are calculated using the lasting special places status subgoal model (except this calculation is based on the entire eez region vs. 3 nm offshore), using the total marine protected area (km2) within the offshore eez region (see the **Offshore coastal protected areas** layer for information about the data). Following to the lasting special places model, a reference point of 30% is used, such that any region with 30%, or more, protected area receives a score of 1.

#### Units

scaled 0-1

## 6.28 EEZ protected marine areas (habitat preservation)

#### hd\_mpa\_eez

*Resilience*

*Category: ecological/regulatory*

*Subcategory: habitat*

See **EEZ protected marine areas (fishing preservation)** for information about this layer.

#### Units

scaled 0-1

## 6.29 Fishery catch data

#### fis\_meancatch

Fisheries catch data were averaged across years (e.g., catch values are the same across years within a taxa/region) for each OHI region and taxon. These values were used to weight the stock status scores (derived from B/Bmsy values) in the fisheries model. Yearly catch data (tonnes) from the Sea Around Us Project (SAUP, Zeller and Pauly 2007) is reported at 0.5 degree resolution for taxonomic levels range from species to class to “Miscellaneous not identified”. Tonnes of catch for each taxon and year were summed within each OHI region, and then the average catch across years was determined for each taxon and region.

#### Units

tonnes

## 6.30 Food provision weights

#### fp\_wildcaught\_weight

To weight the relative contributions of fisheries and mariculture to the food provision goal, we calculate the tonnes of fisheries production (SAUP data, Zeller and Pauly 2007) relative to the total tonnes of food production from fisheries and mariculture (FAO data, FAO Global Aquaculture Production Quantity).

#### Units

proportion

## 6.31 GDP

#### le\_gdp

National GDP data (World Bank, http://data.worldbank.org/indicator/ NY.GDP.MKTP.CD) were used in the economy subgoal to adjust revenue data. For the three EEZs that fall within the China region (China, Macau, and Hong Kong), we combined the values using a population-weighted average.

#### Units

2012 USD

## 6.32 GDP per capita PPP

#### le\_gdp\_pc\_ppp

See **Economic need for artisanal fishing** layer for details.

#### Units

USD

## 6.33 Genetic escapes

#### sp\_genetic

*Pressure*

*Category: ecological*

*Subcategory: nonindigenous species*

This layer represents the potential for harmful genetic escapement based on whether the species being cultured is native or introduced. Data come from the Mariculture Sustainability Index (MSI, Trujillo 2008). In the MSI analysis native species receive the highest score (10), while foreign and introduced species receive the lowest (1) on the premise of potential impacts to local biodiversity if these species were to escape. Use of native but non-local species were scored intermediately based on the assumption that potentially negative alterations to genetic biodiversity occur from non-local sources as well, but to a lower degree. Genetic ‘pollution’ can arise when larvae, spats or seeds escape from poorly managed hatcheries, making native species vulnerable to outbreeding depressions and/or genetic bottlenecks. The MSI reports data for 359 country-species combinations (with 53 countries represented). Where multiple scores exist for a country the weighted average of all scores (0-10) is used. All country scores were then rescaled from 0 to 1, using the maximum raw score of 10 and minimum of 1. Countries that were not analyzed by Trujillo did not receive a score and this pressure layer falls out of their analyses.

#### Units

scaled 0-1

## 6.34 Global Competitiveness Index (GCI) scores

#### li\_gci

*Resilience*

*Category: social*

The World Economic Forum’s Global Competitiveness Index (GCI) provides a country level assessment of competitiveness in achieving sustained economic prosperity (Schwab 2011, http://gcr.weforum.org). The GCI is a weighted index based on 12 pillars of economic competitiveness: institutions, infrastructure, macroeconomic environment, health and primary education, higher education and training, goods market efficiency, labor market efficiency, financial market development, technological readiness, market size, business sophistication, and innovation. The GCI can in theory span from 1 to 7, based on this range, we rescaled the scores to range between 0 and 1.

#### Units

scaled

## 6.35 Habitat condition

#### hab\_health

See **Habitat extent** layer for more information.

#### 6.35.0.1 Coral

Coral condition was calculated using current condition data divided by reference condition. We used condition data from percent live coral cover from 12,634 surveys from 1975-2006 (Bruno and Selig 2007, Schutte et al. 2010). When multiple data points were available for the same site and year, we averaged these data, and also averaged the site data to calculate a per country per year average. However, data were missing for several countries and some countries did not have data for the reference or current year time periods or had only 1-2 surveys. Because coral cover can be highly temporally and spatially dynamic, having only a few surveys that may have been motivated by different reasons (i.e., documenting a pristine or an impacted habitat) can bias results. To calculate condition we used fitted values from a linear trend of all data per country, which was more robust to data poor situations and allowed us to take advantage of periods of intense sampling that did not always include both current and reference years. Then, we created a fitted linear model of all these data points from 1975-2010, provided that 2 or more points are in 1980-1995 and 2 or more points are in 2000-2010. We defined the ‘current’ condition (health) as the mean of the predicted values for 2008-2010, and the reference condition as the mean of the predicted values for 1985-1987. Where country data were not available, we used an average from adjacent EEZs weighted by habitat area, or a georegional average weighted by habitat area, based on countries within the same ocean basin (Figure 6.1).

**Figure 6.1. Georegions to gapfill coral reef data**

#### 6.35.0.2 Mangroves

Mangrove condition was defined as the current cover divided by reference cover. FAO mangrove area data was provided on a country basis for 1980, 1990, 2000, and 2005. Current condition is based on 2005 cover, and reference condition is based on the 1980 cover.

#### 6.35.0.3 Salt marsh

Salt marsh condition was based on trends in area for regions where both a current and reference data year were available. An increasing or stable trend was assigned condition = 1.0, and a decreasing trend was assigned condition = 0.5. Data was from multiple sources (Bridgham et al. 2006, Dahl 2000, Ministry for the Environment 2007, JNCC 2004, EEA 2008).

#### 6.35.0.4 Sea-ice

Sea-ice condition was calculated using sea-ice concentrations from the USA National Snow and Ice Data Center (Cavalieri et al. 2014; https://nsidc.org/cryosphere/quickfacts/seaice.html) as the current percent cover of sea-ice (average of 3 years of data) divided by the average historical percent cover, defined as the start of the data (1979) until the year 2000 as recommended by the National Snow & Ice Data Center for both sea-ice edge and sea-ice shoreline habitats.

#### 6.35.0.5 Seagrass

Seagrass condition was calculated on a per-site basis from Waycott et al. (2009), which provides seagrass habitat extent data for several sites around the world over several years. Reference condition was calculated as the mean of the three oldest years between 1975-1985, or the two earliest years if needed. If data meeting these conditions was not available, we fitted a linear model to all data points, and then used the mean of the predicted values for 1979-1981 as the reference condition. For the current condition we used the mean of the three most recent years after 2000 or the two most recent years. If condition data satisfying these constraints were still not available, we fitted a linear model to all data points, provided that there were at least three data points and then used the mean of the predicted values for 2008-2010 as the current condition and the mean of the predicted values for 1979-1981 as the reference condition. Otherwise, we used neighboring (adjacent) regional averages, weighted by habitat area, or averages weighted by habitat area using seagrass geographical regions as defined by Hemminga and Duarte (2000). We did not project beyond a 15-year timeframe.

#### 6.35.0.6 Soft bottom subtidal

Soft bottom subtidal habitat condition was estimated using intensity of trawl fishing relative to soft bottom subtidal habitat area as a proxy. Spatialized catch data (0.5 degree raster) are available from the global catch database of the Sea Around Us project (Zeller and Pauly 2007), which uses data from FAO global fisheries catch statistics, data from international and national fisheries agencies, and reconstructed catch datasets. The proportion of catch by fishing gear type was estimated at 0.5 degree resolution based on global taxon-gear associations (Watson et al. 2006a,b, Halpern 2008). These data were used to calculate catch (tonnes per year) from trawling for each year. Trawling gears were defined as dredges, hand dredges, bottom trawls, and shrimp trawls (mid-water trawls were excluded). These catches were assumed to be uniform within each half degree cell. We aggregated by summing the trawled catch data by reporting region for each year, and converted to catch density by dividing annual catch by the area of trawlable (soft-bottom) habitat. ‘Trawlable habitat’ within a region was defined as shallow subtidal (0-60m) and outer shelf (60-200m) soft bottom habitat. Because the density data were extremely skewed, we \(ln(X+1)\) transformed them and then rescaled to the 95th quantile value from all year-country possibilities. Condition was then calculated as one minus the rescaled catch density in the most recent year and further rescaled to the median condition value across all years, and any value greater than the median was set = 1.0.

#### Units

proportion

## 6.36 Habitat condition trend

#### hab\_trend

See **Habitat extent** layer for more information.

#### 6.36.0.1 Coral

Coral trend was calculated using condition data from 1975-2006 (Bruno and Selig 2007, Schutte et al. 2010).

#### 6.36.0.2 Mangroves

We used Hamilton and Casey’s (2014) mangrove cover data to estimate the proportional yearly change in mangrove area using a linear regression model of the most recent five years of data (i.e., slope divided by data from the earliest year included in the regression model). The slope was then multiplied by five to get the predicted change in 5 years. The original mangrove data are provided yearly (2000-2012) at 30m raster cell resolution (with the estimated area of mangrove cover in each cell).

#### 6.36.0.3 Salt marsh

General trends in salt marsh area were determined from 1994-2007 using data from multiple sources (Bridgham et al. 2006, Dahl 2000, Ministry for the Environment 2007, JNCC 2004, EEA 2008). For trend estimates, we extracted categorical condition data (‘increasing”, “stable”, “declining” score as 0.5, 0.0, and -0.5, respectively) from these sources on a per country basis when both a current and reference data year were available.

#### 6.36.0.4 Sea-ice

Trends for sea-ice edge and sea-ice shoreline habitats were calculated using sea-ice concentrations from the USA National Snow and Ice Data Center (Cavalieri et al. 2014; https://nsidc.org/cryosphere/quickfacts/seaice.html). The average yearly proportional change in extent was estimated using a linear regression model that included the most recent five years of data (e.g., slope estimate was divided by the extent for earliest year included in the regression model), and this value was multiplied by five to get the predicted change in 5 years. Each year of data represents a 3-year average, to smooth yearly variation.

#### 6.36.0.5 Seagrass

Trend in seagrass condition was determined using two data sources (Waycott et al. 2009, Short et al. 2010). Short et al. (2010) measured percent cover on a per sample, per site, per year basis, whereas Waycott et al. (2009) measured habitat area on a per site, per year basis. We used data from Short if there were at least 3 data points between 2001-2010. If this condition was not met, we calculated trends from Waycott for the most recent 10 years after 1990 or else we used the mean of the trend in the adjacent regions or the regions within the corresponding seagrass geographical regions using the same methods described above for the in status.

#### 6.36.0.6 Soft bottom subtidal

Trend in soft bottom subtidal condition was estimated using a linear regression model that included the most recent five years of condition data. The proportional change in condition was determined (e.g., slope estimate was divided by the condition value for earliest year included in the regression) and then multiplied by five to get the change predicted in 5 years.

#### Units

trend

## 6.37 Habitat extent

#### hab\_extent

**Table 6.5. Habitats used in goal/subgoal calculations**

| Habitat | Habitat goal | Coastal Protection | Carbon Storage | Natural Products (exposure) |
| --- | --- | --- | --- | --- |
| Seagrass | X | X | X |  |
| Mangrove | X | X | X |  |
| Saltmarsh | X | X | X |  |
| Coral | X | X |  | X |
| Sea-ice shoreline |  | X |  |  |
| Sea-ice edge | X |  |  |  |
| Rocky reef |  |  |  | X |
| Soft bottom subtidal | X |  |  |  |

#### 6.37.0.1 Coral

Coral extent area (km2) are derived from the 500m resolution dataset developed for Reefs at Risk Revisited (Burke et al. 2011).

#### 6.37.0.2 Mangroves

Hamilton and Casey’s (2014) yearly (2000-2012) mangrove cover data were used to calculate mangrove extent (km2). Data were provided at 30m raster cell resolution (with the estimated area of mangrove cover in each cell) which we converted to 500m resolution to facilitate global calculations.

#### 6.37.0.3 Salt marsh

Salt marsh extent area (km2) comes from multiple sources (Bridgham et al. 2006, Dahl 2000, Ministry for the Environment 2007, JNCC 2004, EEA 2008). In the case of Europe, most data were obtained via the European Environment Agency databases housing information pertaining to the European Union’s Habitat Directive.

Severe data gaps exist for several key regions of the world, including the Middle East, South America, and Africa. Extensive salt marshes are believed to exist in the Middle East, bordering the unique salt flat ecosystems known as sabkha, however no numbers concerning extent could be found. Similarly, data on salt marshes in South America were extremely limited, with no estimates on current rates of loss or historical extent from or before the 1970s. The majority of the data included in the model come from North America (United States and Canada), Australia, New Zealand, China, Europe, and the United Kingdom.

#### 6.37.0.4 Rocky reef

To estimate rocky reef extent area (km2) we used data from Halpern et al. (2008), which assumes rocky reef habitat exists in all cells within 1 km of shore.

#### 6.37.0.5 Sea-ice

Sea-ice extent area (km2) was calculated using sea-ice concentrations from the USA National Snow and Ice Data Center (Cavalieri et al. 2014; https://nsidc.org/cryosphere/quickfacts/seaice.html), which are updated yearly. These raster data are 25km in resolution (625km2 per pixel) in a Stereographic polar projection. Two sea-ice metrics are calculated using these data: sea-ice edge (pixels with 10-50% ice cover) and sea-ice shoreline (shoreline pixels with >15% ice cover). Calculations of area are based on 3-year averages (to smooth yearly variation, e.g., 2009 data is the average of 2008-2010) of the pixels meeting the habitat criteria.

#### 6.37.0.6 Seagrass

Seagrass extent area (km2) was calculated from vector-based data from the Global Distribution of Seagrasses (UNEP-WCMC 2005).

#### 6.37.0.7 Soft bottom subtidal

Soft bottom extent was defined as the total area (km2) of shallow (0-60m) and shelf (60-200m) soft-bottom habitat within the reporting region based on benthic substrate point samples (Halpern et al. 2008).

#### Units

km2

## 6.38 Habitat weights

#### element\_wts\_hab\_pres\_abs

This layer describes the habitats present in each region (based on the habitat extent data) and is called internally by ohicore functions to calculate pressure and resilience values based on the habitats present in each region.

#### Units

boolean

## 6.39 High bycatch due to artisanal fishing

#### fp\_art\_hb

*Pressure*

*Category: ecological*

*Subcategory: fishing pressure*

Reefs at Risk Revisited (Burke et al. 2007) recorded the global presence of destructive artisanal blast and poison (cyanide) fishing based on survey observations and expert opinion. We reclassified the log-scale scoring system for the blast and poison rasters, so 0 = 0, 100 = 1, 1000 = 2. The mean raster score was then determined for each OHI region. The poison and blast values for each region were then summed to get the total.

A notable limitation of using these data as a proxy for high bycatch artisanal fishing is that they only cover coral reef habitats. High bycatch artisanal fishing likely exists in every coastal country on the planet, but we have no way of knowing the type or extent (for every country).

#### Units

scaled 0-1

## 6.40 High bycatch due to commercial fishing

#### fp\_com\_hb

*Pressure*

*Category: ecological*

*Subcategory: fishing pressure*

This layer describes the relative pressure of high bycatch commercial fishing practices for each OHI region. These data are calculated using 0.5 degree global raster data for: (1) **Industrial** fish catch reconstruction data from the Sea Around Us Project (SAUP) that describe tons of commercial catch; and, (2) the proportion of catch for each of 5 commercial fishing gear types (Halpern 2008). High bycatch commercial fishing is defined as total catch caught by demersal destructive (e.g., trawl), demersal non-destructive high bycatch (e.g., pots, traps) and pelagic high bycatch (e.g., long-line) gear. For each raster cell, the proportion of catch using high bycatch gear was multiplied by the total tonnes of catch to produce a single layer describing the tonnes of catch using high bycatch methods.

Catch per cell is then standardized by mean net primary productivity (mg C/m2/day) derived from monthly output from the Catch per cell was then standardized by mean net primary productivity (mg C/m2/day) derived from monthly output from the Vertically Generalized Production Model (VGPM, http://www.science.oregonstate.edu/ocean.productivity/). Standardizing catch by primary productivity controls for the fact that similar amounts of catch impart different pressures depending on the productivity in the region. The layer was rescaled from 0 to 1 using the 99.99th quantile of the entire data layer, and the mean value of the raster cells within each OHI region was calculated.

#### Units

scaled 0-1

## 6.41 Inland area

#### rgn\_area\_inland1km

See **Region areas based on EEZ boundaries** layer for more information.

Area (km2) located from each region’s land-sea interface to 1 km inland.

For coastal land areas, we extracted hi-resolution country boundary data from ESRI (2010), and rasterized it with a resolution to match our land-sea interface model. We grew this raster by 50 pixels to bridge gaps between the ESRI data and our land-sea model. Area values do not include inland lakes or EEZs.

#### Units

km2

## 6.42 Inland coastal population

#### mar\_coastalpopn\_inland25mi

Coastal population was determined for each region using Gridded Population of the World (GPW) Population Count Grid Future Estimates (v3) from the Center for International Earth Science Information Net (CIESIN)/Columbia University (CIESIN & CIAT 2005). Global data were provided at 2.5 arc-minute resolution for 2005, 2010, and 2015. Intervening years were temporally interpolated, as: d2013 = 0.4 \* d2010 + 0.6 \* d2015. The total coastal population count data was calculated for each region by extracting the relevant cell data using a 25-mile inland coastal zone shapefile. Where modeled coastal population exceeded World Bank reported total population, World Bank figures were used. We used other sources (e.g., World Bank, Wikipedia, etc.) to estimate population for some island regions (Macquarie Island, Wake Island, Glorioso Islands, Juan de Nova Island, Bassas da India, Ile Europa, Ile Tromelin, British Indian Ocean Territory, Kerguelen Islands, Heard and McDonald Islands, Bouvet Island, Clipperton Island, Jan Mayen, Palmyra Atoll, Johnston Atoll, South Georgia and the South Sandwich Islands, Prince Edward Islands, Crozet Islands, Amsterdam Island and Saint Paul Island).

#### Units

number of people

## 6.43 Inland coastal protected areas

#### lsp\_prot\_area\_inland1km

This includes protected areas 1km inland, but otherwise follows the methods described in **Offshore coastal protected areas**.

#### Units

km2

## 6.44 Intertidal habitat destruction

#### hd\_intertidal

*Pressure*

*Category: ecological*

*Subcategory: habitat destruction*

See **Inland coastal population** for more information about the population data.

Coastal population data was converted to average coastal density by dividing by the total 25 mile inland area. We then rescaled the data to have values between 0-1, by logging the density data and then dividing by the ln (maximum density) across all regions and years.

#### Units

scaled 0-1

## 6.45 ISO country codes

#### cntry\_rgn

Country codes for OHI regions based on ISO 3166 country codes.

#### Units

region id

## 6.46 IUCN extinction risk

#### ico\_spp\_iucn\_status

This data layer provides the risk category and the year the species was assessed from the IUCN Red List of Threatened Species (http://www.iucnredlist.org/) for the iconic species in each region. Regionally specific IUCN risk category data for *subpopulations* are included where available. Trend calculations are based on the change in each species’ IUCN risk category over time, based upon past and current IUCN assessments.

OHI defines iconic species as those relevant to local cultural identity through the species’ relationship to traditional activities such as fishing, hunting, commerce or involvement in local ethnic or religious practices; and species with locally-recognized aesthetic value (e.g., touristic attractions/common artistic subjects such as whales). Habitat forming species are excluded in this definition of iconic species. The OHI global iconic species list combines three species lists from WWF Global: global priorities, regional and local priorities, and flagship species. The criteria for including species on the WWF lists are consistent with the OHI’s definition of iconic species.

Once the species lists were obtained, each species was assigned to a region based on native range countries from the IUCN Red List.

Most of the iconic species are not region specific, and the global list is applied across all regions. However, some countries have developed national priority and flagship species lists in conjunction with WWF. These region-specific iconic species lists supplement the global list for those specific countries only. In addition, as countries and regions conduct OHI regional assessments (http://ohi-science.org/projects/), we will use the iconic species list developed by those countries/regions to supplement our global model. For example, iconic species identified for the Baltic Health Index regional assessment have been included for all countries bordering the Baltic Sea.

**Table 6.6. Iconic species resources**

| Iconic List | Source |
| --- | --- |
| Priority Species | http://wwf.panda.org/what\_we\_do/endangered\_species/ |
| Flagship Species | http://wwf.panda.org/what\_we\_do/endangered\_species/ |
| Australia’s Flagship Species | http://www.wwf.org.au/our\_work/saving\_the\_natural\_world/wildlife\_and\_habitats/australian\_priority\_species/ |
| Pakistan’s Priority Species | http://www.wwfpak.org/species/priority\_species.php |
| India’s Priority Species | http://www.wwfindia.org/about\_wwf/priority\_species/ |
| Madagascar’s Flagship Species | http://www.wwf.mg/ourwork/cssp/species\_report/wwf\_madagascar\_s\_flagship\_species/ |
| Malaysia’s Flagship Species | http://www.wwf.org.my/about\_wwf/what\_we\_do/species\_main/ |
| Portugal’s Flagship Species | http://www.wwf.pt/o\_nosso\_planeta/especies/top\_5\_das\_especies\_de\_portugal\_\_\_as\_cinco\_especies\_mais\_ameacadas\_e\_ emblematicas\_de\_portugal/ |
| Peru’s Priority Species | http://peru.panda.org/nuestro\_trabajo/iniciativas\_globales/ |

#### Units

IUCN risk category

## 6.47 Jobs for each sector and year

#### le\_jobs\_sector\_year

This layer includes yearly data for jobs in commercial fishing, mariculture, marine mammal watching, marine renewable energy, and, tourism. The data sources and methods for each sector are described below.

#### 6.47.0.1 Commercial fishing

Data are from the United Nations Food and Agriculture Organization (FAO) Fisheries and Aquaculture Department which provides a Global Number of Fishers dataset ( http://www.fao.org/fishery/statistics/global-fishers/en). The data include yearly total numbers of employees in commercial fishing, subsistence fishing, and aquaculture (land- and ocean-based combined) in more than 160 countries. The dataset includes the following occupational categories: aquatic-life cultivation, inland waters fishing, marine coastal waters fishing, marine deepsea waters fishing, subsistence and unspecified. We omitted jobs with an unspecified category to avoid overestimating employment for marine fishing or aquaculture. We omitted jobs in the subsistence category since subsistence opportunities are captured by the artisanal fishing opportunity goal and in the aquatic-life cultivation category since that represents a distinct sector (see mariculture below). For commercial fishing, we eliminated inland waters fishing and summed marine coastal waters and marine deep-sea waters fishing for each country in each year. Data are reported separately for men and women, but we summed these numbers. Employment is disaggregated into full-time, parttime, occasional, and unspecified statuses. These categories are defined as full time workers having > 90% of their time or livelihood from fishing/aquaculture, part time workers are between 30-90% time (or 30-90% of their livelihood) and occasional workers are < 30% time. Unspecified status workers could fall anywhere from 0-100% time. Taking the midpoints of those ranges, we assume that 1 part time worker = 0.6 full time workers, 1 occasional worker = 0.15 full time workers, and 1 unspecified worker = 0.5 full time workers, which we used as a weighting scheme for determining total numbers of jobs. The dataset has significant gaps, but it provides the most comprehensive source of global data on commercial fishing and aquaculture employment.

#### 6.47.0.2 Mariculture

We used the FAO Global Number of Fishers dataset (see commercial fishing above for full description) to estimate jobs for mariculture. For this sector, we used data in the aquatic-life cultivation category. Again, employment is disaggregated into full-time, part-time, occasional, and unspecified statuses and we implement a weighting scheme where full time = 1 job, part-time = 0.6, occasional = 0.15, and unspecified = 0.5. Aquatic-life cultivation includes marine, brackish and freshwater aquaculture. In order to estimate the proportion of total aquaculture jobs that can be attributed to marine and brackish aquaculture, we used country-specific proportions of marine and brackish aquaculture revenues (compared to total revenues) calculated from FAO aquaculture production data, assuming that numbers of jobs approximately scale with production in terms of revenue. For country-years with no data for the proportion of marine/brackish production because of gaps in the FAO production data, we used the proportion from the most recent year for which data were available. For countries without proportion estimates from any years, we used the average proportion from the country’s geographic region (e.g., Caribbean, Polynesia, Eastern Asia), with the exception of American Samoa, for which we used the proportion value from Guam.

#### 6.47.0.3 Marine mammal watching

The International Fund for Animal Welfare’s (IFAW) Whale Division provides time series data on whale watching in more than 115 coastal countries (O’Connor et al 2009). This dataset may be an imperfect representation of all marine mammal watching due to its focus on whales, although it does include data for other types of marine mammal watching (e.g., dolphins). However, to our knowledge, it is the most complete dataset pertaining to the global marine mammal watching industry. We obtained regional averages of the number of whale watchers per employee, as well as the number of whale watchers in each country. Using this information, we estimated the number of whale watching jobs in each country by dividing the country’s total number of whale watchers by the average number of whale watchers per employee for that country’s region (e.g., Africa & Middle East, Europe, North America). It is important to note that data are not annual, but there are at least four years of data for each country. When IFAW reported “minimal” numbers of whale watchers, we converted this description to a 0 for lack of additional information. Because some of the whale watching in O’Connor et al. focused on freshwater cetacean viewing, we categorized the target species listed for each country as freshwater or marine. For countries with both marine and freshwater species, we categorized the whale watching in those countries as either 50% or 90% marine, based on the number of marine versus freshwater target species and information provided in the report narrative. For Colombia and Indonesia, more detailed information in the report narrative allowed for a more precise determination of the percentage of marine-based whale watching. We applied these marine proportions to data on the number of whale watchers before converting these estimates into employment estimates.

#### 6.47.0.4 Marine renewable energy

The number of marine renewable energy jobs was determined for the two countries, France and Canada, which produce significant enough amounts of tidal energy to register with the UN Energy Statistics Database http://data.un.org/Data.aspx?d=EDATA&f=cmID%3aEO. For the La Rance plant in France, employment information was obtained from a recent press statement (EDF 2011); we assumed employment stayed constant over the time period for which we had production data for this plant, given relatively consistent or even growing production. For the Annopolis Royal plant in Canada, we received yearly employment information from the plant (Ruth Thorbourne, personal communication, Aug 9 2011).

Marine renewable energy includes five major technologies: tidal barrages, marine currents, waves, ocean thermal converters and salinity gradients. However, we only include data for the largest tidal barrage plants, as these data are available.

#### 6.47.0.5 Tourism

The World Travel & Tourism Council (WTTC) provides data on travel and tourism’s total contribution to employment for 180 countries (http://www.wttc.org/eng/Tourism\_Research/Economic\_Data\_Search\_Tool/). Although other global data sources on tourism are available (i.e., United Nations World Tourism Organization, UNTWO), the WTTC database was chosen because it offers yearly time series data that span through the current year, it includes nearly complete coverage of all nations, and it disaggregates direct and total (direct plus indirect) employment impacts of tourism. WTTC provides projected data, however, we do not use these values. We used total employment data to avoid the use of literature derived multiplier effects. The WTTC shares a significant drawback with UNTWO data, in that data on coastal/marine and inland tourism are lumped. Therefore, a country-specific coefficient must be applied to estimate the jobs provided by coastal/marine tourism alone. We adjusted national tourism data by the proportion of a country’s population that lives within a 25 mile inland coastal zone.

#### Units

number of jobs

## 6.48 Jobs, most current value

#### le\_jobs\_cur\_base\_value

See **Jobs for each sector and year** layer for information about data and methods.

This layer includes the jobs data (from the **Jobs for each sector and year** layer) for the most recent year of data (relative to the assessment year) for each sector/region.

#### Units

value

## 6.49 Jobs, reference value

#### le\_jobs\_ref\_base\_value

See **Jobs for each sector and year** layer for information about data and methods.

This layer includes the jobs data (from the **Jobs for each sector and year** layer) for the earliest year of data for each sector/region.

#### Units

value

## 6.50 Low bycatch due to artisanal fishing

#### fp\_art\_lb

*Pressure*

*Category: ecological*

*Subcategory: fishing pressure*

This layer estimates the relative pressure of artisanal fishing using catch reconstruction data from the Sea Around Us Project (SAUP) at a resolution of 0.5 degrees globally. Artisanal catch (tons) includes 3 of the 4 SAUP reporting sectors: artisanal, subsistence and recreational catch. The catch from these sectors was aggregated for each raster cell, and then standardized by mean net primary productivity (mg C/m2/day) derived from monthly output from the Vertically Generalized Production Model (VGPM, http://www.science.oregonstate.edu/ocean.productivity/).

Catch per cell was then standardized by mean net primary productivity (mg C/m2/day) derived from monthly output from the Vertically Generalized Production Model (VGPM, http://www.science.oregonstate.edu/ocean.productivity/). Standardizing catch by primary productivity controls for the fact that similar amounts of catch impart different pressures depending on the productivity in the region. The layer was rescaled from 0 to 1 using the 99.99th quantile of the entire data layer, and the mean value of the raster cells within each OHI region was calculated.

#### Units

scaled 0-1

## 6.51 Low bycatch due to commercial fishing

#### fp\_com\_lb

*Pressure*

*Category: ecological*

*Subcategory: fishing pressure*

This layer describes the relative pressure of low bycatch commercial fishing practices for each OHI region. These data are calculated using 0.5 degree global raster data for: (1) **Industrial** fish catch reconstruction data from the Sea Around Us Project (SAUP) that describe tons of commercial catch; and, (2) the proportion of catch for each of 5 commercial fishing gear types (Halpern 2008). Low bycatch commercial fishing is defined as total catch caught by demersal low bycatch (e.g., hook and line) and pelagic low bycatch (e.g., purse seines) gear. For each raster cell, the proportion of catch using low bycatch methods was multiplied by the total tonnes of catch to produce a single layer describing the tonnes of catch using low bycatch methods.

Catch per cell was then standardized by mean net primary productivity (mg C/m2/day) derived from monthly output from the Vertically Generalized Production Model (VGPM, http://www.science.oregonstate.edu/ocean.productivity/). Standardizing catch by primary productivity controls for the fact that similar amounts of catch impart different pressures depending on the productivity in the region. The layer was rescaled from 0 to 1 using the 99.99th quantile of the entire data layer, and the mean value of the raster cells within each OHI region was calculated.

#### Units

scaled 0-1

## 6.52 Management of habitat to protect fisheries biodiversity

#### fp\_habitat

*Resilience*

*Category: ecological/regulatory*

*Subcategory: fishing*

Country responses to the Convention on Biological Diversity (CBD) Third National Report (2005). Each question was weighted equally within each category and responses were averaged to give a score between 0 and 1 for all responding countries. The survey uses a 0 to 3 scale for questions 79 and 81, and a 0 to 2 scale for question 80, which we rescale linearly to 0 to 1.

All countries were given credit within each of the 4 resilience measures for simply being a member of the CBD (0.5), the other 0.5 of the resilience score came from each country’s response to the specific questions within each resilience measure. In cases where the “European Union” answered yes or was a signatory, all EU25 countries were given that answer if they did not provide one themselves.

The CBD has 193 members and 153 members responded to the Third National Survey (2005). We had data for 147 regions, and used geographical means, weighted by country area, for the remaining regions.

Questions: 153 (a,b,c,e,g) and 158 (a,b,c,f,g,h)

153. Do your country’s strategies and action plans include the following?

1. Developing new marine and coastal protected areas
2. Improving the management of existing marine and coastal protected areas
3. Building capacity within the country for management of marine and coastal resources, including through educational programmes and targeted research initiatives
4. Protection of areas important for reproduction, such as spawning and nursery areas
5. Controlling excessive fishing and destructive fishing practices

158. Which of the following statements can best describe the current status of marine and coastal protected areas in your country?

1. Marine and coastal protected areas have been declared and gazetted
2. Management plans for these marine and coastal protected areas have been developed with involvement of all stakeholders
3. Effective management with enforcement and monitoring has been put in place
4. The national system of marine and coastal protected areas includes areas managed for purpose of sustainable use, which may allow extractive activities
5. The national system of marine and coastal protected areas includes areas which exclude extractive uses
6. The national system of marine and coastal protected areas is surrounded by sustainable management practices over the wider marine and coastal environment.

#### Units

scaled 0-1

## 6.53 Management of habitat to protect habitat biodiversity

#### hd\_habitat

*Resilience*

*Category: ecological/regulatory*

*Subcategory: habitat*

See **Management of habitat to protect fisheries biodiversity**  layer for data and methods.

#### Units

scaled 0-1

## 6.54 Management of mariculture to preserve biodiversity

#### g\_mariculture

*Resilience*

*Category: ecological/regulatory*

*Subcategory: goal*

Country responses to the Convention on Biological Diversity (CBD) Third National Report (2005). Each question was weighted equally within each category and responses were averaged to give a score between 0 and 1 for all responding countries. The survey uses a 0 to 3 scale for questions 79 and 81, and a 0 to 2 scale for question 80, which we rescale linearly to 0 to 1.

All countries were given credit within each of the 4 resilience measures for simply being a member of the CBD (0.5), the other 0.5 of the resilience score came from each country’s response to the specific questions within each resilience measure. In cases where the “European Union” answered yes or was a signatory, all EU25 countries were given that answer if they did not provide one themselves.

The CBD has 193 members and 153 members responded to the Third National Survey (2005). We had data for 147 regions, and used geographical means, weighted by country area, for the remaining regions.

Questions: 158 (d) and 159 (a-l)

158. Which of the following statements can best describe the current status of marine and coastal protected areas in your country?

4. A national system or network of marine and coastal protected areas is under development

159. Is your country applying the following techniques aimed at minimizing adverse impacts of mariculture on marine and coastal biodiversity?

1. Application of environmental impact assessments for mariculture developments
2. Development and application of effective site selection methods in the framework of integrated marine and coastal area management
3. development of effective methods for effluent and waste control
4. Development of appropriate genetic resource management plans at the hatchery level
5. Development of controlled hatchery and genetically sound reproduction methods in order to avoid seed collection from nature.
6. If seed collection from nature cannot be avoided, development of environmentally sound practices for spat collecting operations, including use of selective fishing gear to avoid by-catch
7. Use of native species and subspecies in mariculture
8. Implementation of effective measures to prevent the inadvertent release of mariculture species and fertile polypoids.
9. Use of proper methods of breeding and proper places of releasing in order to protect genetic diversity
10. Minimizing the use of antibiotics through better husbandry techniques
11. Use of selective methods in commercial fishing to avoid or minimize bycatch
12. Considering traditional knowledge, where applicable, as a source to develop sustainable mariculture techniques

#### Units

scaled 0-1

## 6.55 Management of nonindigenous species

#### sp\_alien\_species

*Resilience*

*Category: ecological/regulatory*

*Subcategory: nonindigenous species*

Country responses to the Convention on Biological Diversity (CBD) Third National Report (2005). Each question was weighted equally within each category and responses were averaged to give a score between 0 and 1 for all responding countries. The survey uses a 0 to 3 scale for questions 79 and 81, and a 0 to 2 scale for question 80, which we rescale linearly to 0 to 1.

All countries were given credit within each of the 4 resilience measures for simply being a member of the CBD (0.5), the other 0.5 of the resilience score came from each country’s response to the specific questions within each resilience measure. In cases where the “European Union” answered yes or was a signatory, all EU25 countries were given that answer if they did not provide one themselves.

The CBD has 193 members and 153 members responded to the Third National Survey (2005). We had data for 147 regions, and used geographical means, weighted by country area, for the remaining regions.

Questions: 160 (b-e)

160. Has your country put in place mechanisms to control pathways of introduction of alien species in the marine and coastal environment? Please check all that apply and elaborate on types of measures in the space below.

1. No
2. Mechanisms to control potential invasions from ballast water have been put in place
3. Mechanisms to control potential invasions from hull fouling have been put in place (please provide details below)
4. Mechanisms to control potential invasions from aquaculture have been put in place (please provide details below)
5. Mechanisms to control potential invasions from accidental releases, such as aquarium releases, have been put in place (please provide details below)

#### Units

scaled 0-1

## 6.56 Management of tourism to preserve biodiversity

#### g\_tourism

*Resilience*

*Category: ecological/regulatory*

*Subcategory: goal*

Country responses to the Convention on Biological Diversity (CBD) Third National Report (2005). Each question was weighted equally within each category and responses were averaged to give a score between 0 and 1 for all responding countries. The survey uses a 0 to 3 scale for questions 79 and 81, and a 0 to 2 scale for question 80, which we rescale linearly to 0 to 1.

All countries were given credit within each of the 4 resilience measures for simply being a member of the CBD (0.5), the other 0.5 of the resilience score came from each country’s response to the specific questions within each resilience measure. In cases where the “European Union” answered yes or was a signatory, all EU25 countries were given that answer if they did not provide one themselves.

The CBD has 193 members and 153 members responded to the Third National Survey (2005). We had data for 147 regions, and used geographical means, weighted by country area, for the remaining regions.

Questions: 79, 80, 82

79. Has your country established mechanisms to assess, monitor and measure the impact of tourism on biodiversity?

1. No
2. No, but mechanisms are under development
3. Yes, mechanisms are in place (please specify below)
4. Yes, existing mechanisms are under review

80. Has your country provided educational and training programmes to the tourism operators so as to increase their awareness of the impacts of tourism on biodiversity and upgrade the technical capacity at the local level to minimize the impacts?

1. No
2. No, but programmes are under development
3. Yes, programmes are in place (please describe below)

82. Does your country provide indigenous and local communities with capacity-building and financial resources to support their participation in tourism policy-making, development planning, product development and management?

1. No
2. No, but relevant programmes are being considered
3. Yes, some programmes are in place
4. Yes, comprehensive programmes are in place

#### Units

scaled 0-1

## 6.57 Management of waters to preserve biodiversity

#### po\_water

*Resilience*

*Category: ecological/regulatory*

*Subcategory: water*

Country responses to the Convention on Biological Diversity (CBD) Third National Report (2005). Each question was weighted equally within each category and responses were averaged to give a score between 0 and 1 for all responding countries. The survey uses a 0 to 3 scale for questions 79 and 81, and a 0 to 2 scale for question 80, which we rescale linearly to 0 to 1.

All countries were given credit within each of the 4 resilience measures for simply being a member of the CBD (0.5), the other 0.5 of the resilience score came from each country’s response to the specific questions within each resilience measure. In cases where the “European Union” answered yes or was a signatory, all EU25 countries were given that answer if they did not provide one themselves.

The CBD has 193 members and 153 members responded to the Third National Survey (2005). We had data for 147 regions, and used geographical means, weighted by country area, for the remaining regions.

Questions: 153 (d,f)

153. Do your country’s strategies and action plans include the following?

4. Instituting improved integrated marine and coastal area management (including catchments management) in order to reduce sediment and nutrient loads into the marine environment
5. Improving sewage and other waste treatment

#### Units

scaled 0-1

## 6.58 Mariculture harvest

#### mar\_harvest\_tonnes

Mariculture production from the FAO Global Aquaculture Production Quantity dataset. Only production classified in the “Marine” and “Brackishwater” environments was included in the analysis (all “Freshwater” production was excluded). Seaweeds were excluded because they are included in the natural products goal. All species produced within a country were summed to give a single production value for each country in each year that production took place. For the three EEZs that fall within the China region (China, Macau, and Hong Kong), we combined the values by summing across these EEZs.

#### Units

tonnes

## 6.59 Mariculture Sustainability Index

#### g\_msi\_gov

*Resilience*

*Category: ecological/regulatory*

*Subcategory: goal*

See **Mariculture sustainability score** for more information about these data.

Two mariculture practice criteria from the Marine Sustainability Index (Trujillo 2008) contributed to the resilience of mariculture (traceability and code of practice). These are the only 2 social criteria assessed in the MSI that have the potential to positively affect the long term resilience of a mariculture system.

The MSI reports data for 359 country-species combinations (with 60 countries and 86 species represented) for each assessment criterion. Scores for each assessment criterion were aggregated and averaged. All country average scores were then rescaled from 0 to 1 using the maximum possible raw MSI score of 10 and minimum of 1, and then weighted equally to come up with a composite resilience.

Unlike the mariculture sustainability data (**Mariculture sustainability score**), gapfilling was not performed when country specific data were not available for the two resilience measures because these measures are social in nature and reliant on the specific decisions made by each country rather than on the species cultured. As such, it was decided that crosscountry species averages were not appropriate to use in the gapfilling process for resilience.

**Table 6.7. Mariculture Sustainability Index criteria**

| Criteria | Description of practice and score scheme |
| --- | --- |
| Code of practice usage | Certification, up to date set of standards and principles, i.e., FAO Code of Conduct (FAO 1995, 1999), or Eco-labelling are scored high, while no certification or similar scheme scores low (1) |
| Traceability | Food safety related to a specific geographical origin, slaughtering or processing facility, and batch of fish can be identified scores high (8-9). If the original and preparation of feed used in the farmed sector is included then scores high (10). |

#### Units

scaled 0-1

## 6.60 Mariculture sustainability score

#### mar\_sustainability\_score

Three mariculture practice criteria from the Marine Sustainability Index (Trujillo 2008) contributed to the sustainability of mariculture (fishmeal use, waste treatment, and seed and larvae origin criteria). These criteria represent the internal mariculture practices with the potential to affect the long term sustainability of the mariculture system. The MSI reports data for 359 country-species combinations (with 60 countries and 86 species represented) for each assessment criterion. Scores for each assessment criterion were aggregated and averaged based on the proportion of the landings that each assessed species contributed to the overall catch in each country in the current year. All country average scores were then rescaled from 0 to 1 using the maximum possible raw MSI score of 10 and minimum of 1, and then weighted equally to calculate a composite resilience. Because the Index comprises species-specific sustainability values that are combined as a catch-weighted average, when the list of species harvested changes, the sustainability index used in the mariculture sub-goal model also changes due to shifts in the relative composition of harvested species.

Species/country combinations that were not assessed by the MSI were gapfilled using average data from similar taxonomic groupings from other countries (ideally we used the same species, but used more general taxonomic groupings when necessary).

**Table 6.8. Mariculture sustainability criteria**

| Criteria | Description of practice and score scheme |
| --- | --- |
| Fishmeal use | Fish protein and oil inclusion in the diet at any stage of development must be considered; herbivore species will score 10, and carnivorous (piscivorous) organisms will score closer to 1, depending on the level of feed supplied. |
| Waste treatment | Water exchange, output destinations, recycling and filtering of open water discharge or closed system reuse systems. Systems that are closed score high (10), while open systems without waste treatments score low (1) |
| Seed and larvae origin | Hatcheries are major providers of larvae, fry and seeds. Broodstock origin and strain will also affect the score. Wild seed collection and its importance contribute to a low score due to bycatch and other effects on non-target species. |

#### Units

score

## 6.61 Marine plastics

#### po\_trash

*Pressure*

*Category: ecological*

*Subcategory: pollution*

Marine plastic pollution is modeled using data on the global distribution of floating marine plastics at 0.2 degree resolution (Erikson et al. 2014). Specifically, weight of floating plastics (g/km2) across four different size classes were aggregated to represent total weight of plastic debris per km2. These data were log transformed and rescaled from 0 to 1 using the 99.99th quantile as the reference point.

#### Units

scaled 0-1

## 6.62 Measure of coastal ecological integrity

#### species\_diversity\_3nm

*Resilience*

*Category: ecological/ecosystem*

See Species goal for calculations.

This value reflects the average condition of species (based on risk status from the IUCN Red List of Threatened Species, http://www.iucnredlist.org/) located within 3 nm offshore of each region based on species range maps from IUCN (shapefiles, used preferentially) and Aquamaps (http://www.aquamaps.org/, half degree resolution rasters).

#### Units

scaled 0-1

## 6.63 Measure of ecological integrity

#### species\_diversity\_eez

*Resilience*

*Category: ecological/ecosystem*

See Species goal for calculations.

This value reflects the average condition of species (based on risk status from the IUCN Red List of Threatened Species, http://www.iucnredlist.org/) located within the eez of each region based on species range maps from IUCN (shapefiles, used preferentially) and Aquamaps (http://www.aquamaps.org/, half degree resolution rasters).

#### Units

scaled 0-1

## 6.64 Natural product harvest

#### np\_harvest\_tonnes

The total tonnes of six natural product commodities (corals, ornamental fish, shells, fish oil, seaweeds and plants, and sponges) were determined for each region using export data from the FAO Global Commodities database. For each group the sum of the subcategories was calculated. For ornamental fish we excluded the subcategory ‘Fish for culture including ova, fingerlings, etc.’ because it is not specific to ornamental fish, and the subcategory ‘Ornamental freshwater fish’ because it is not from marine systems. Corals and shells are reported together in nine subcategories; we used the subcategories most closely tied to each, leading to the sum of two subcategories for corals and seven subcategories for shells (one subcategory was used for both). We did not use one subcategory – ‘Powder and waste of shells’ – as it likely a byproduct of the other subcategories rather than a primary target (and it comprises a very small amount of the total anyway).

If a country was missing tonnes or dollar values (but had one of the values), the missing data were estimated. FAO provides yearly data for the tonnes and dollar value generated for each natural product, however, countries often provide only one of these variables (and the data provided varies across years). To estimate these missing data, we used country-specific linear models to predict tonnes based on the dollar value of a product (or, vice versa). For the countries that did not have enough data to develop an adequate model, our models included the data for all the countries within a UN geopolitical region. When there wasn’t enough data at the geopolitical region scale, we used all the global data to predict missing values.

**Table 6.9. FAO categories included in each natural product commodity**

| commodity | subcategory |
| --- | --- |
| corals | Coral and the like |
| fish oil | Alaska pollack oil, nei, Anchoveta oil, Capelin oil, Clupeoid oils, nei, Cod liver oil, Fish body oils, nei, Fish liver oils, nei, Gadoid liver oils, nei, Hake liver oil, Halibuts, liver oils, Herring oil, Jack mackerel oil, Menhaden oil, Pilchard oil, Redfish oil, Sardine oil, Shark liver oil, Shark oil, Squid oil, Pelagic fish oils, nei, Gadiformes, oil, nei, Demersal fish oils, nei, Alaska pollock, oil, nei |
| ornamentals | Ornamental saltwater fish, Ornamental fish nei |
| seaweeds | Agar agar in powder, Agar agar in strips, Agar agar nei, Carrageen (Chondrus crispus), Green laver, Hizikia fusiforme (brown algae), Kelp, Kelp meal, Laver, dry, Laver, nei, Other brown algae (laminaria, eisenia/ecklonia), Other edible seaweeds, Other inedible seaweeds, Seaweeds and other algae, unfit for human consumption, nei, Seaweeds and other algae, fit for human consumption, nei, Other red algae, Other seaweeds and aquatic plants and products thereof, Undaria pinnafitida (brown algae) |
| shells | Abalone shells, Miscellaneous corals and shells, Mother of pearl shells, Oyster shells, Sea snail shells, Shells nei, Trochus shells |
| sponges | Natural sponges nei, Natural sponges other than raw, Natural sponges raw |

#### Units

tonnes

## 6.65 Nonindigenous species

#### sp\_alien

*Pressure*

*Category: ecological*

*Subcategory: nonindigenous species*

These data (Molnar et al. 2008) report the number and type of alien species in each marine ecoregion (Spalding et al. 2007), with species types categorized as invasive and harmful invasive species. For our purposes, total count of all invasive species was used. We intersected the ecoregion data with our reporting units to determine the proportion of each ecoregion that falls within each reporting unit and then assigned this percentage of invasive species from the ecoregion to the reporting unit. The sum of all invasive species within each reporting unit was then rescaled to the maximum global value. Predicting the full potential impact of alien species depends in large part on having high-resolution spatial information on where they exist, how far they have spread and exactly which components of the food web they affect. The data from Molnar et al. approximate these impacts but at ecoregional scales. In addition, the impacts of alien species will vary depending on the goal under consideration. This implies that harmful effects would need to be assessed separately for each goal. Such an endeavor may be possible when applying this framework to a smaller case-study where this type of information can be acquired.

#### Units

scaled 0-1

## 6.66 Nutrient pollution

#### po\_nutrients

*Pressure*

*Category: ecological*

*Subcategory: pollution*

Data were calculated using modeled plumes of land-based nitrogen pollution that provide intensity of pollution at ~1km resolution (Halpern et al. 2008).

Nitrogen pollution was estimated from FAO data on annual country-level fertilizer use (http://faostat3.fao.org/faostat-gateway/go/to/browse/R/\*/E), with missing values estimated by regression between fertilizer and pesticides when possible, and when not possible with agricultural GDP as a proxy. Data were summed across all fertilizer compounds and reported in metric tons. Upon inspection the data included multiple 0 values that are most likely data gaps in the time-series, so they were treated as such and replaced with NA. In addition, regions with only 1 data point and regions where the most recent data point was prior to 2005 were excluded. Uninhabited countries were assumed to have no fertilizer use and thus excluded.

These country-level pollution values were then dasymetrically distributed over a country’s landscape using global landcover data from 2009, derived from the MODIS satellite at ~500m resolution. These values were then aggregated by ~140,000 global basins, and diffusive plumes were modeled from each basin’s pourpoint. The final non-zero plumes (about ~76,000) were aggregated into ~1km Mollweide (wgs84) projection rasters to produce a single plume-aggregated pollution raster.

These raw values were then \(ln(X+1)\) transformed and normalized to 0-1 by dividing by the 99.99th quantile of raster values across all years. The zonal mean was then calculated for each region.

#### Units

scaled 0-1

## 6.67 Nutrient pollution trend

#### cw\_nutrient\_trend

See description for nutrient pollution layer **Nutrient pollution**.

The inverse of the pressure data (1 - **Coastal nutrient pollution**) was used to estimate nutrient trends for the clean water goal. The proportional yearly change was estimated using a linear regression model of the most recent five years of data (i.e., slope divided by data from the earliest year included in the regression model). The slope was then multiplied by five to get the predicted change in 5 years.

#### Units

trend

## 6.68 Ocean acidification

#### cc\_acid

*Pressure*

*Category: ecological*

*Subcategory: climate change*

This pressure layer models the difference in global distribution of the aragonite saturation state (\(\Omega\_{arag}\)) of the ocean in the pre-industrial era and modern times. Global estimates through time (Feely et al. 2009) are modeled at 1-degree resolution. Changes in the saturation state can be attributed to changes in the concentration of CO2 and thus we use the difference between the pre-industrial and modern times as a proxy for ocean acidification due to human influences. Values are rescaled from 0 to 1 using the threshold at which seawater becomes undersaturated, where \(\Omega\_{arag} = 1\).

#### Units

scaled 0-1

## 6.69 Offshore area

#### rgn\_area\_offshore3nm

See **Region areas based on EEZ boundaries** layer for more information.

Area (km2) located from each region’s land-sea interface to 3nm offshore.

#### Units

km2

## 6.70 Offshore coastal protected areas

#### lsp\_prot\_area\_offshore3nm

This includes marine protected areas within 3nm offshore of the coastline.

Data is from the United Nations Environment Programme - World Conservation Monitoring Centre’s World Database on Protected Areas (WDPA, http://www.protectedplanet.net). Data includes all nationally designated (e.g., National Parks, Nature Reserves) and internationally recognized protected areas (e.g., UNESCO World Heritage Sites, Ramsar Wetlands of International Importance) as an ESRI shapefile. We used only WDPA polygons (not points) with a status of “designated” (not “proposed”). These polygons were converted to a 500 m Mollweide raster by the value of the year in which the park was decreed “designated”. For cases in which polygons overlapped, priority was given first to the parks with the earliest year. The total amount of protected area (km2) was calculated for each year for: the entire eez, 3 nm offshore, and 1km inland (depending on the dimension being calculated).

#### Units

km2

## 6.71 OHI regions

#### rgn\_global

OHI global region ID and name.

#### Units

label

## 6.72 Pathogen pollution

#### po\_pathogens

*Pressure*

*Category: ecological*

*Subcategory: pollution*

The percentage of the population with access to improved sanitation facilities (World Health Organization and United Nations Children’s Fund, Joint Monitoring Programme, 2011) was used in combination with measurements of coastal population as a proxy for pathogens in coastal waters. Access to improved sanitation facilities is defined as the percentage of the population in a country with at least adequate access to disposal facilities that can effectively prevent human, animal, and insect contact with excreta. These data are a country-wide average (not specific to the coastal region). Percentages (0-100) for each country were rescaled to 0-1 based on a maximum target of 100% of the population with access to improved sanitation, and a minimum value of 0.

#### Units

scaled 0-1

## 6.73 Pathogen pollution trend

#### cw\_pathogen\_trend

See description of data and methods in **Pathogen pollution** layer.

The proportional yearly change in pathogen pressure values were estimated using a linear regression model of the most recent five years of data (i.e., slope divided by data from the earliest year included in the regression model). The slope was then multiplied by five to get the predicted change in 5 years.

#### Units

trend

## 6.74 Percent direct employment in tourism

#### tr\_jobs\_pct\_tourism

World Travel & Tourism Council (www.wttc.org/research/economic-data-search-tool/) provides country data describing the percent employment in the travel and tourism sectors (such as hotels, airlines, airports, travel agents and leisure & recreation services that deal directly with tourists). We used the percent employment in “direct” tourism jobs as an indicator of the number of tourists visiting the coast.

Regions without data were gapfilled using the average values of UN geopolitical regions.

#### Units

proportion

## 6.75 Plastic trash trends

#### cw\_trash\_trend

Trend in trash was estimated using data on improperly disposed of plastics (Jambeck et al. 2016). Data are from the supplement, and describe mismanaged plastic waste in 2010 and projected mismanaged plastic waste in 2025. Using these data, we estimates proportional trash trends during a 5 year period as follows:

\(trend\_{15year} = (value\_{2025} - value\_{2010})/value\_{2010}\)

\(trend\_{1year} = trend\_{15year}/15\)

The trend\_1year value was then multiplied by 5 to get the estimated proportional change in 5 years.

Missing data was gapfilled using a linear regression model with population as a predictor.

#### Units

trend

## 6.76 Region areas based on EEZ boundaries

#### rgn\_area

Area (km2) for each region’s EEZ-based boundary.

OHI offshore regions are based on exclusive economic zones (EEZ, VLIZ 2012). Unique country EEZs were typically used to define a region, except territorial regions were split from the administrative country. Many borders have been redrawn, such as the removal of UK claims around Cyprus. Gaps and extensions between this EEZ file and our land-sea mask were resolved through GIS operations (buffer, erase, and polygon neighbor analysis). Ocean area per region was calculated using geodesic area calculations on the region polygons in geographic coordinates. We exclude from regions the inland EEZs of the Caspian Sea and any disputed areas.

#### Units

km2

## 6.77 Regions

#### rgn\_labels

OHI region ids for eez (1-250) and fao high seas regions (260-278).

#### Units

label

## 6.78 Relative harvest tonnes

#### np\_harvest\_tonnes\_relative

See **Natural product harvest** layer for more information about these data.

Within each region, the harvest of each commodity was scaled relative to its maximum value across all years. The tonnes of each commodity is divided by the maximum value observed for the commodity in each region, with a 35% buffer applied to avoid penalizing yearly variation and to prevent overharvesting (i.e., max value times 0.65).

#### Units

proportion

## 6.79 Relative harvest value

#### np\_harvest\_product\_weight

See **Natural product harvest** layer for more information about these data.

Within each region and year, the value (USD) of harvest of each commodity relative to total harvest value of six marine commodities (coral, fish oil, seaweed and plants, shells, sponges, ornamental fish). Used to weight contribution of each product to final natural product status score.

#### Units

proportion

## 6.80 Revenue adjustment (GDP)

#### le\_revenue\_adj

National GDP data were obtained from the World Bank (http://data.worldbank.org/indicator/ NY.GDP.MKTP.CD). These data were used in economy subgoal to adjust revenue data. For the three EEZs that fall within the China region (China, Macau, and Hong Kong), we combined the values using a population-weighted average.

#### Units

USD

## 6.81 Revenue for each sector and year

#### le\_rev\_sector\_year

This layer includes yearly data for revenue in commercial fishing, aquarium trade fishing, mariculture, marine mammal watching, marine renewable energy, and, tourism. The data sources and methods for each sector are described below.

#### 6.81.0.1 Aquarium trade fishing

To approximate revenue from aquarium fishing we used export data from the FAO Global Commodities database for ‘Ornamental fish’ for all available years. We used data from two of the four subcategories listed, excluding the subcategory ‘Fish for culture including ova, fingerlings, etc.’ because it is not specific to ornamental fish, and the subcategory ‘Ornamental freshwater fish’ because it is not from marine systems.

#### 6.81.0.2 Commercial fishing

Revenue data for commercial fishing were obtained from FAO’s FishStat database, which provides yearly dollar values of commercial fisheries production for marine, brackish and freshwater species starting in 1950 and updated yearly. To isolate production values attributable to marine and brackish aquaculture, data pertaining to freshwater species were omitted. This species classification process was very time consuming as each species had to be queried individually per year. There was little year-to year variation, and thus data were extracted in 5 year increments, providing data for 1997, 2002 and 2007.

#### 6.81.0.3 Mariculture

Data on revenues from marine aquaculture were derived from FAO’s FishStat database, which includes country-level data on total production values for marine, brackish, and freshwater species beginning in 1984 and updated yearly. To isolate production values attributable to marine and brackish aquaculture, data pertaining to freshwater species were omitted. This species classification process was very time consuming as each species had to be queried individually per year. There was little year-to year variation, and thus data were extracted in 5 year increments, providing data for 1997, 2002 and 2007.

#### 6.81.0.4 Marine mammal watching

IFAW (see **Jobs for each sector and year** layer) provides time series (for at least four years between 1991 and 2008), country-level data on total expenditures (including direct and indirect) attributable to the whale watching industry (O’Connor et al. 2009). Here, total expenditures are used as a close proxy for total revenue. We used total expenditure data (direct and indirect expenditures) to avoid using a literature derived multiplier effect. When IFAW reported “minimal” revenue from whale watching, we converted this description to a 0 for lack of additional information. For countries with both marine and freshwater cetacean viewing, we adjusted by the proportion of marine revenue as described for the jobs dataset.

#### 6.81.0.5 Marine renewable energy

The United Nations Energy Statistics Database provides production data, in kilowatt-hours (KWh), for tidal and wave electricity. However, only two countries, France and Canada, have high enough levels of production to be reported in this data source. For Canada, production data were replaced with production data (Gross Megawatt hours per year from 1995-2010) provided directly from the Annapolis tidal power plant because the plant provided a longer time series (Ruth Thorbourne, personal communication, Aug 9, 2011). To convert production data into revenue, production values were multiplied by average yearly prices of electricity per KWh specific to Canada and France, provided by the US Energy Information Administration (http://www.eia.gov/emeu/international/elecprii.html; updated June 2010) after conversion to 2010 USD. Some of the production data could not be used because there were no available electricity price data to convert production into revenue, truncating our time series.

#### 6.81.0.6 Tourism

WTTC (see **Jobs for each sector and year** layer) reports time series data on the dollar values of visitor exports (spending by foreign visitors) and domestic travel and tourism spending; combining these two data sets creates a proxy for total travel and tourism revenues. WTTC was chosen as the source for tourism revenue data because of the near-complete country coverage, the yearly time series component starting in 1988 and updated yearly, and the inclusion of both foreign and domestic expenditures. This dataset lumps inland and coastal/marine revenues, and so was adjusted by the percent of a country’s population within a 25 mile inland coastal zone. We included no projected data. We used total contribution to GDP data (rather than direct contribution to GDP) to avoid the use of literature derived multiplier effects.

#### Units

2010 USD

## 6.82 Revenue, most current value

#### le\_rev\_cur\_base\_value

See **Revenue for each sector and year** layer for information about data and methods.

This layer describes revenue data (from the **Revenue for each sector and year** layer) for the most recent year of data (relative to the assessment year) for each sector/region.

#### Units

value

## 6.83 Revenue, reference value

#### le\_rev\_ref\_base\_value

See **Revenue for each sector and year** layer for information about data and methods.

This layer describes revenue data (from the **Revenue for each sector and year** layer) for the earliest year of data for each sector/region.

#### Units

value

## 6.84 Sea level rise

#### cc\_slr

*Pressure*

*Category: ecological*

*Subcategory: climate change*

The sea level rise pressure layer is derived from satellite altimetry data (http://www.aviso.altimetry.fr/en/data/products/sea-surface-height-products/global/msla-mean-climatology.html). Monthly mean sea level anomalies from 1993 through 2015 track changes in sea level (mm) compared to a reference period from 1993-2012. Raw monthly data are provided on a 0.25x0.25 degree grid. These data were clipped to within 3 nautical miles of the coast, and monthly data layers were aggregated and averaged across pixels to compute mean sea level anomalies. The 99.99th quantile of raster values from all years was used as the reference point to rescale the layer from 0 to 1. All negative values were set to zero (i.e., no negative pressure), such that only positive sea level rise values mattered. The mean value of the raster cells within each OHI region was calculated.

#### Units

scaled 0-1

## 6.85 Sea surface temperature

#### cc\_sst

*Pressure*

*Category: ecological*

*Subcategory: climate change*

Sea surface temperature (SST) data were obtained from the Coral Reef Temperature Anomaly Database (CoRTAD) (Casey et al. 2015), which is produced by the NOAA National Center for Environmental Information (NCEI) using 4.6 km (nominally 21 km2 at the equator) Advanced Very High Resolution Radiometer (AVHRR) Pathfinder Version 5.2 SST data (http://www.nodc.noaa.gov/sog/cortad/). Weekly SST data are used to compute the standard deviation (SD) of SST’s per pixel across all years. We define an anomaly as exceeding the standard deviation of SSTs from the climatology for that location (i.e., grid cell) and week of the year. The frequency of weekly anomalies was calculated for each year in the dataset. We then quantified the difference between the number of anomalies in the 5 most recent years and the 5 oldest years in the dataset. The 99.99th quantile of raster values from all years was used as the reference point to rescale the layer from 0 to 1, and the mean value of the raster cells within each OHI region was calculated.

Because SST measurements are less reliable where there is persistent ice, we created an ice mask to identify places near the poles that were almost always covered by significant sea ice. The ice mask was generated primarily from the OSI/SAF Global Daily Sea Ice Concentration Reprocessing Data Set (accession.nodc.noaa.gov/0068294), which was regridded and made available in the Pathfinder V5.2 dataset. In Pathfinder, when the OSI/SAF data are unavailable, the sea ice concentrations from the NCDC Daily OI SST data (Reynolds et al. 2007) are included. For each day of the climatological year (1 through 366), we read in the daily sea ice fraction for that day from all of the years and averaged them to create a daily, sea-ice fraction climatology. We then identified grid cells that always contained a sea ice fraction of greater than 0.15 and masked them out of the analysis.

#### Units

scaled 0-1

## 6.86 Sectors in each region

#### le\_sector\_weight

Describes which livelihood and economy sectors are present in each region.

#### Units

value

## 6.87 Social Progress Index

#### res\_spi

*Resilience*

*Category: social*

The Social Progress Index (http://www.socialprogressimperative.org/global-index/) includes several quality of life measures. The SPI score is the average of 3 dimensions, and each dimension is the average of 4 components. Each component includes several indicators that are scaled from 0 to 100. The SPI is not calculated for any region with 1 or more missing subcomponents. In these cases, we used a linear regression model of the subcomponents with data to estimate the missing subcomponent values. Regions without data were gapfilled with predicted values from a linear regression model that included UN geopolitical region and WGI values as predictor variables. Uninhabited regions received no score.

#### Units

scaled 0-1

## 6.88 Strength of governance

#### wgi\_all

*Resilience*

*Category: social*

The Worldwide Governance Indicator (WGI) is composed of six dimensions of governance: voice and accountability, political stability and absence of violence, government effectiveness, regulatory quality, rule of law, control of corruption. These 6 aggregate indicators combine data from a variety of survey institutes, think tanks, NGOs, and international organizations to report on the relative governance of 213 economies worldwide. The WGI combines individual indicators through an Unobserved Components Model to produce the 6 dimensions of governance that range in value from approximately -2.5 to 2.5, have a normal distribution, a mean of zero, and a standard deviation of 1. We take an average of the six dimension scores to produce a single governance score for each country. Social pressure is then calculated as one minus this average WGI score.

WGI scores are provided for China/Hong Kong/Macao and Puerto Rico/Virgin Island, which are combine OHI regions. These scores were averaged, weighted by population.

#### Units

scaled 0-1

## 6.89 Subtidal hardbottom habitat destruction

#### hd\_subtidal\_hb

*Pressure*

*Category: ecological*

*Subcategory: habitat destruction*

See **High bycatch due to artisanal fishing** for description of data and methods.

These data are from Reefs at Risk Revisited (Burke et al. 2007) and describe the prevalence of blast fishing.

#### Units

scaled 0-1

## 6.90 Subtidal soft bottom habitat destruction

#### hd\_subtidal\_sb

*Pressure*

*Category: ecological*

*Subcategory: habitat destruction*

See **Habitat condition** layer to get more information about this layer.

The Pressure score was calculated as one minus soft-bottom habitat condition.

#### Units

scaled 0-1

## 6.91 Targeted harvest of cetaceans and marine turtles

#### fp\_targetharvest

*Pressure*

*Category: ecological*

*Subcategory: fishing pressure*

This data layer describes the pressure on cetaceans and marine turtles for each country calculated using the FAO Global Capture Production Quantity dataset. We extracted all catch records from the FAO data for cetaceans or marine turtles and aggregated to create a total reported catch count for each region. The summed catch was rescaled from 0-1, using the 95th quantile across all years (including and prior to the assessment year) and regions (values > 1 were capped at 1).

#### Units

scaled 0-1

## 6.92 Total human population

#### le\_popn

Population data are from the World Bank (http://data.worldbank.org/indicator/SP.POP.TOTL). We gapfilled 59 regions with no reported data using Wikipedia. Those estimates were for a single year, so to fill missing years we calculated the average per-year change in population across all regions in the World Bank data, and then applied those percent changes to the single year data.

#### Units

number of people

## 6.93 Tourism sustainability index

#### tr\_sustainability

The Travel and Tourism Competitiveness Index is produced by the World Economic Forum and measures the factors and policies that make a country an attractive place to invest in the travel and tourism sector (WEF 2015, http://reports.weforum.org/travel-and-tourism-competitiveness-report-2015). The index analyzes 140 countries and scores each based on three sub-indices: human, cultural, and natural resources; business environment and infrastructure; and regulatory framework. These three sub-indices are in turn composed of 14 “pillars” of Travel & Tourism Competitiveness that are informed by a multitude of individual indicators based on the World Economic Forum’s annual Executive Opinion Survey and data from publically available sources: human, cultural, and natural resources (human resources, affinity for travel and tourism, natural resources, and cultural resources); business environment and infrastructure (air transport infrastructure, ground transport infrastructure, tourism infrastructure, ICT infrastructure, and price competitiveness in the industry); and regulatory framework (policy rules and regulations, environmental sustainability, safety and security, health and hygiene, and prioritization of travel and tourism). Because these indicators are meant to represent the overall quality and future potential of the tourism sector within a country, we assume they are representative of the long term sustainability of the tourism sector within each country. Values range from 1-6.

For countries not assessed, values were estimated using a linear regression model specific to each UN geopolitical region using per-capita GDP as a predictor variable.

#### Units

score

## 6.94 Unemployment

#### le\_unemployment

Unemployment data were from the World Bank (http://data.worldbank.org/indicator/SL.UEM.TOTL.ZS). These data are reported as the percent of the total labor force that is available to work and seeking employment but is without work. For the three EEZs that fall within the China region (China, Macau, and Hong Kong), we combined the values using a population-weighted average.

#### Units

proportion

## 6.95 UN geopolitical region classifications

#### rgn\_georegions

UN geopolitical region classifications (United Nations Statistics Division, 2016; http://unstats.un.org/unsd/methods/m49/m49regin.htm). These data are often used for gapfilling missing data. Level r0 is the most general classification, and all OHI regions have the same classification (World), r1 includes seven geopolitical classes (Africa, Americas, etc.), and r2 includes 22 geopolitical classes (Caribbean, Central America, etc.).

#### Units

UN regions

## 6.96 US State Department travel warnings

#### tr\_travelwarnings

Travel advisories are from the U.S. State Department (https://travel.state.gov/content/passports/en/alertswarnings.html). Penalty scores were based on the urgency of warning (‘risk’=0.25, ‘avoid unnecessary travel’=0.75, and ‘avoid all travel’ = 1). Penalties for region-specific warnings (within a country) were assessed at half the weight.

#### Units

score

## 6.97 UV radiation

#### cc\_uv

*Pressure*

*Category: ecological*

*Subcategory: climate change*

The ultraviolet radiation (UV) pressure layer is derived from daily Local Noon Erythemal UV Irradiance (mW/m2) data. The Aura/OMI satellite provides data at 1x1 degree resolution from September 2004 through present, spanning 180 degrees latitude and 360 degrees longitude. Raster data are provided in HDF5 format by the NASA Goddard Earth Sciences Data and Information Services Center (GESDISC, (http://disc.sci.gsfc.nasa.gov/Aura/data-holdings/OMI/omuvbd\_v003.shtml). Raw data was downloaded, translated to GeoTIFFs using R and aggregated to weekly means.

This pressure measures the number of times the weekly average of each 1 degree cell exceeds the climatological mean + 1 standard deviation, defined as an anomalous value. The frequency of weekly anomalies was calculated for each year in the dataset. We then quantified the difference between the number of anomalies in the 5 most recent years and the 5 oldest years in the dataset. The 99.99th quantile of raster values from all years was used as the reference point to rescale the layer from 0 to 1, and the mean value of the raster cells within each OHI region was calculated.

#### Units

scaled 0-1

## 6.98 Wages, current value

#### le\_wage\_cur\_base\_value

See **Wages for each sector and year** layer for information about data and methods.

This layer describes the wage data (from the **Wages for each sector and year** layer) for the most recent year of data (relative to the assessment year) for each sector/region.

#### Units

value

## 6.99 Wages for each sector and year

#### le\_wage\_sector\_year

Note: These data are no longer supported. Consequently, this layer was last updated in 2013, and this goal will no longer be updated with these data.

We used the Occupational Wages around the World (OWW) database produced by Remco H. Oostendorp and Richard B. Freeman in 2005 (http://www.nber.org/oww/). These data were drawn from the International Labour Organization and subjected to a standardization process (for more information, see http://www.nber.org/oww/Technical \_document\_1983-2003\_standardizationv3.pdf). The database provides several different calibrations, and we use the “x3wl calibration”, described as a “country-specific and uniform calibration with lexicographic weighting,” and recommended as being the preferred calibration in most cases. Although significant gaps exist in this database, it contains country-specific information on average wages in many industries for more than 150 countries from 1983-2003. Data represent average monthly wages of a male worker. Wage data were divided by the inflation conversion factor for 2010 so that wage data across years would be comparable (http://oregonstate.edu/cla/polisci/sahr/sahr), and then multiplied by the purchasing power parity-adjusted per capita gdp (ppppcgdp, WorldBank). The adjusted wage data were then multiplied by 12 to get annual wages. We used the industry and occupation classifications reported in the OWW to estimate wages for marine-related sectors.

**Table 6.10. Occupation classification for wage data sectors**

| Sector | Occupation classifications |
| --- | --- |
| Commercial fishing | Industry: deep sea & coastal fishing;  Occupations: deep sea fisher; inshore (coastal) maritime fisherman |
| Ports & harbors | Industry: supporting services to maritime transport;   Occupation: dock worker |
| Ship & boat building | Industry: shipbuilding and repairing;   Occupation: ship plater |
| Tourism | Industry: restaurants and hotels;   Occupations: hotel receptionist; cook; waiter; room attendant or chambermaid.   These data are not specific to coastal/marine tourism jobs, and thus we assumed that wages in these jobs are equal in coastal and non-coastal areas |
| Transportation & shipping | Industry: maritime transport;   Occupations: ship’s chief engineer; ship’s passenger stewards; able seaman |

#### Units

2010 USD

## 6.100 Wages, reference value

#### le\_wage\_ref\_base\_value

See **Wages for each sector and year** layer for information about data and methods.

This layer describes the wage data (from the **Wages for each sector and year** layer) for the earliest year of data for each sector/region.

#### Units

value

## 6.101 Weakness of governance

#### ss\_wgi

*Pressure*

*Category: social*

See **Strength of governance** for description of this layer.

When used as a social pressure, 1 minus the WGI (Worldwide Governance Indicator) is used.

#### Units

scaled 0-1

## 6.102 Weakness of social progress

#### ss\_spi

*Pressure*

*Category: social*

See **Social Progress Index** for description of this layer.

When used as a social pressure, 1 minus the SPI (Social Progress Index) is used.

#### Units

scaled 0-1

# References

Allison, E.H. & Ellis, F. (2001). The livelihoods approach and management of small-scale fisheries. *Marine Policy*, **25**, 377–388.

Anderson, S.C., Cooper, A.B., Jensen, O.P., Minto, C., Thorson, J.T., Walsh, J.C., Afflerbach, J., Dickey-Collas, M., Kleisner, K.M., Longo, C., Osio, G.C., Ovando, D., Mosqueira, I., Rosenberg, A.A. & Selig, E.R. (2017). Improving estimates of population status and trend with superensemble models. *Fish and Fisheries*, n/a–n/a.

Attaran, M. (1986). Industrial diversity and economic performance in u.S. areas. *The Annals of Regional Science*, **20**, 44–54.

AVISO. (2016). Satellite alimetry data: Mean sea level rise. URL http://www.aviso.altimetry.fr/en/data/products/ocean-indicators-products/mean-sea-level.html [accessed 18 March 2016]

Barnosky, A.D., Matzke, N., Tomiya, S., Wogan, G.O.U., Swartz, B., Quental, T.B., Marshall, C., McGuire, J.L., Lindsey, E.L., Maguire, K.C., Mersey, B. & Ferrer, E.A. (2011). Has the earth’s sixth mass extinction already arrived? *Nature*, **471**, 51–57.

Behrenfeld, M.J. & Falkowski, P.G. (1997). Photosynthetic rates derived from satellite-based chlorophyll concentration. *Limnology and oceanography*, **42**, 1–20.

Bird species distribution maps of the world. (2015).

Borja, A., Bricker, S.B., Dauer, D.M., Demetriades, N.T., Ferreira, J.G., Forbes, A.T., Hutchings, P., Jia, X., Kenchington, R., Marques, J.C. & Zhu, C. (2008). Overview of integrative tools and methods in assessing ecological integrity in estuarine and coastal systems worldwide. *Marine Pollution Bulletin*, **56**, 1519–1537.

Bridgham, S.D., Megonigal, J.P., Keller, J.K., Bliss, N.B. & Trettin, C. (2006). The carbon balance of north american wetlands. *Wetlands*, **26**, 889–916.

Bruno, J.F. & Selig, E.R. (2007). Regional decline of coral cover in the indo-pacific: Timing, extent, and subregional comparisons (R. Freckleton, Ed.). *PLoS ONE*, **2**, e711.

Burgass, M.J., Halpern, B.S., Nicholson, E. & Milner-Gulland, E.J. (2017). Navigating uncertainty in environmental composite indicators. *Ecological Indicators*, **75**, 268–278.

Burke, L., Reytar, K., Spadling, M., Perry, A., Cooper, E., Kushner, B., Selig, E., Starkhouse, B., Teleki, K., Waite, R., Wilkinson, C. & Young, T. (2011). *Reefs at risk - revisited*. World Resources Institute, Washington, D.C.

Butchart, S.H., Resit Akçakaya, H., Chanson, J., Baillie, J.E., Collen, B., Quader, S., Turner, W.R., Amin, R., Stuart, S.N. & Hilton-Taylor, C. (2007). Improvements to the red list index (D. Lusseau, Ed.). *PLoS ONE*, **2**, e140.

Cavalieri, D.J., Parkinson, C.L., Gloersen, P. & Zwally, H.J. (1996). *Sea ice concentrations from nimbus-7 SMMR and DMSP SSM/i-SSMIS passive microwave data, version 1*. NASA National Snow; Ice Data Center Distributed Active Archive Center, Boulder, Colorado USA.

Cinner, J., Daw, T. & McCLANAHAN, T. (2009). Socioeconomic factors that affect artisanal fishers’ readiness to exit a declining fishery. *Conservation Biology*, **23**, 124–130.

CITES. (2015). Member countries. URL https://cites.org/eng/disc/parties/index.php

Claus, S., De Hauwere, N., Vanhoorne, B., Souza Dias, F., Oset Garcia, P., Hernandez, F. & Mees, J. (2012). Marine regions: Exclusive economic zones (v7). URL http://www.marineregions.org/

Convention on Biological Diversity. (2005). 3rd national report, survey results. URL https://www.cbd.int/convention/ [accessed 13 April 2016]

Costello, C., Ovando, D., Clavelle, T., Strauss, C.K., Hilborn, R., Melnychuk, M.C., Branch, T.A., Gaines, S.D., Szuwalski, C.S., Cabral, R.B., Rader, D.N. & Leland, A. (2016). Global fishery prospects under contrasting management regimes. *Proceedings of the National Academy of Sciences*, **113**, 5125–5129.

Costello, C., Ovando, D., Hilborn, R., Gaines, S.D., Deschenes, O. & Lester, S.E. (2012). Status and solutions for the world’s unassessed fisheries. *Science*, **338**, 517–520.

Crotti, R. & Misrahi, T. (Eds.). (2015). *World Economic Forum: The travel & tourism competitiveness report*.

Dahl, T.E. (2011). *Status and trends of wetlands in the conterminous united states 2004 to 2009*. US Department of the Interior, US Fish; Wildlife Service, Fisheries; Habitat Conservation.

Donato, D.C., Kauffman, J.B., Murdiyarso, D., Kurnianto, S., Stidham, M. & Kanninen, M. (2011). Mangroves among the most carbon-rich forests in the tropics. *Nature Geoscience*, **4**, 293–297.

Duarte, C.M. (2000). Marine biodiversity and ecosystem services: An elusive link. *Journal of Experimental Marine Biology and Ecology*, **250**, 117–131.

Eionet, E.E.A.(. (2008). EU habitats directive article 17 reporting, 2001-2006. URL http://bd.eionet.europa.eu/activities/Reporting/Article\_17/reference\_portal

Eriksen, M., Lebreton, L.C.M., Carson, H.S., Thiel, M., Moore, C.J., Borerro, J.C., Galgani, F., Ryan, P.G. & Reisser, J. (2014). Plastic pollution in the world’s oceans: More than 5 trillion plastic pieces weighing over 250,000 tons afloat at sea. *PLoS ONE*, **9**, e111913.

ESRI. (2010). Esri data and maps.

FAO Fisheries and Aquaculture Department. (2015). CWP handbook of fishery statistical standards. section h: FISHING AREAS FOR STATISTICAL PURPOSES.

Feely, R., Doney, S. & Cooley, S. (2009). Ocean acidification: Present conditions and future changes in a high-CO2 world. *Oceanography*, **22**, 36–47.

France, E. (2011). Électricité de france. URL https://www.edf.fr/

Frazier, M., Longo, C. & Halpern, B.S. (2016). Mapping uncertainty due to missing data in the global ocean health index. *PLOS ONE*, **11**, e0160377.

Froese, R., Branch, T.A., Proelß, A., Quaas, M., Sainsbury, K. & Zimmermann, C. (2011). Generic harvest control rules for european fisheries: Generic harvest control rules. *Fish and Fisheries*, **12**, 340–351.

Giri, C., Ochieng, E., Tieszen, L.L., Zhu, Z., Singh, A., Loveland, T., Masek, J. & Duke, N. (2011). Status and distribution of mangrove forests of the world using earth observation satellite data: Status and distributions of global mangroves. *Global Ecology and Biogeography*, **20**, 154–159.

Halpern, B.S., Frazier, M., Potapenko, J., Casey, K.S., Koenig, K., Longo, C., Lowndes, J.S., Rockwood, R.C., Selig, E.R., Selkoe, K.A. & Walbridge, S. (2015a). Spatial and temporal changes in cumulative human impacts on the world’s ocean. *Nature Communications*, **6**, 7615.

Halpern, B.S., Longo, C., Hardy, D., McLeod, K.L., Samhouri, J.F., Katona, S.K., Kleisner, K., Lester, S.E., O’Leary, J., Ranelletti, M., Rosenberg, A.A., Scarborough, C., Selig, E.R., Best, B.D., Brumbaugh, D.R., Chapin, F.S., Crowder, L.B., Daly, K.L., Doney, S.C., Elfes, C., Fogarty, M.J., Gaines, S.D., Jacobsen, K.I., Karrer, L.B., Leslie, H.M., Neeley, E., Pauly, D., Polasky, S., Ris, B., Martin, K.S., Stone, G.S., Sumaila, U.R. & Zeller, D. (2012). An index to assess the health and benefits of the global ocean. *Nature*.

Halpern, B.S., Longo, C., Lowndes, J.S.S., Best, B.D., Frazier, M., Katona, S.K., Kleisner, K.M., Rosenberg, A.A., Scarborough, C. & Selig, E.R. (2015b). Patterns and emerging trends in global ocean health. *PLoS ONE*, **10**, e0117863.

Halpern, B.S., Longo, C., Scarborough, C., Hardy, D., Best, B.D., Doney, S.C., Katona, S.K., McLeod, K.L., Rosenberg, A.A. & Samhouri, J.F. (2014). Assessing the health of the u.S. west coast with a regional-scale application of the ocean health index. *PLoS ONE*, **9**, e98995.

Halpern, B.S., Walbridge, S., Selkoe, K.A., Kappel, C.V., Micheli, F., D’Agrosa, C., Bruno, J.F., Casey, K.S., Ebert, C., Fox, H.E., Fujita, R., Heinemann, D., Lenihan, H.S., Madin, E.M.P., Perry, M.T., Selig, E.R., Spalding, M., Steneck, R. & Watson, R. (2008). A global map of human impact on marine ecosystems. *Science*, **319**, 948–952.

Hamilton, S. & Casey, D. (2016). Creation of a high spatiotemporal resolution global database of continuous mangrove forest cover for the 21st century (CGMFC-21). *Global Ecology and Biogeography*.

Hoffmann, M., Hilton-Taylor, C., Angulo, A., Bohm, M., Brooks, T.M., Butchart, S.H.M., Carpenter, K.E., Chanson, J., Collen, B., Cox, N.A., Darwall, W.R.T., Dulvy, N.K., Harrison, L.R., Katariya, V., Pollock, C.M., Quader, S., Richman, N.I., Rodrigues, A.S.L., Tognelli, M.F., Vie, J.-C., Aguiar, J.M., Allen, D.J., Allen, G.R., Amori, G., Ananjeva, N.B., Andreone, F., Andrew, P., Ortiz, A.L.A., Baillie, J.E.M., Baldi, R., Bell, B.D., Biju, S.D., Bird, J.P., Black-Decima, P., Blanc, J.J., Bolanos, F., Bolivar-G., W., Burfield, I.J., Burton, J.A., Capper, D.R., Castro, F., Catullo, G., Cavanagh, R.D., Channing, A., Chao, N.L., Chenery, A.M., Chiozza, F., Clausnitzer, V., Collar, N.J., Collett, L.C., Collette, B.B., Fernandez, C.F.C., Craig, M.T., Crosby, M.J., Cumberlidge, N., Cuttelod, A., Derocher, A.E., Diesmos, A.C., Donaldson, J.S., Duckworth, J.W., Dutson, G., Dutta, S.K., Emslie, R.H., Farjon, A., Fowler, S., Freyhof, J., Garshelis, D.L., Gerlach, J., Gower, D.J., Grant, T.D., Hammerson, G.A., Harris, R.B., Heaney, L.R., Hedges, S.B., Hero, J.-M., Hughes, B., Hussain, S.A., Icochea M., J., Inger, R.F., Ishii, N., Iskandar, D.T., Jenkins, R.K.B., Kaneko, Y., Kottelat, M., Kovacs, K.M., Kuzmin, S.L., La Marca, E., Lamoreux, J.F., Lau, M.W.N., Lavilla, E.O., Leus, K., Lewison, R.L., Lichtenstein, G., Livingstone, S.R., Lukoschek, V., Mallon, D.P., McGowan, P.J.K., McIvor, A., Moehlman, P.D., Molur, S., Alonso, A.M., Musick, J.A., Nowell, K., Nussbaum, R.A., Olech, W., Orlov, N.L., Papenfuss, T.J., Parra-Olea, G., Perrin, W.F., Polidoro, B.A., Pourkazemi, M., Racey, P.A., Ragle, J.S., Ram, M., Rathbun, G., Reynolds, R.P., Rhodin, A.G.J., Richards, S.J., Rodriguez, L.O., Ron, S.R., Rondinini, C., Rylands, A.B., Sadovy de Mitcheson, Y., Sanciangco, J.C., Sanders, K.L., Santos-Barrera, G., Schipper, J., Self-Sullivan, C., Shi, Y., Shoemaker, A., Short, F.T., Sillero-Zubiri, C., Silvano, D.L., Smith, K.G., Smith, A.T., Snoeks, J., Stattersfield, A.J., Symes, A.J., Taber, A.B., Talukdar, B.K., Temple, H.J., Timmins, R., Tobias, J.A., Tsytsulina, K., Tweddle, D., Ubeda, C., Valenti, S.V., Paul van Dijk, P., Veiga, L.M., Veloso, A., Wege, D.C., Wilkinson, M., Williamson, E.A., Xie, F., Young, B.E., Akcakaya, H.R., Bennun, L., Blackburn, T.M., Boitani, L., Dublin, H.T., Fonseca, G.A.B. da, Gascon, C., Lacher, T.E., Mace, G.M., Mainka, S.A., McNeely, J.A., Mittermeier, R.A., Reid, G.M., Rodriguez, J.P., Rosenberg, A.A., Samways, M.J., Smart, J., Stein, B.A. & Stuart, S.N. (2010). The impact of conservation on the status of the world’s vertebrates. *Science*, **330**, 1503–1509.

Homer, C., Huang, C., Yang, L., Wylie, B. & Coan, M. (2004). Development of a 2001 national land-cover database for the united states. *Photogrammetric Engineering & Remote Sensing*, **70**, 829–840.

Hughes, T.P. (2003). Climate change, human impacts, and the resilience of coral reefs. *Science*, **301**, 929–933.

International Earth Science Information Network (CIESIN), C. for, Columbia University, Agriculture Programme (FAO)), U.F. {and} & Agricultura Tropical (CIAT), C.I. de. (2005). *Gridded population of the world, version 3 (GPWv3): Population count grid future estimates*. Palisades, NY: NASA Socioeconomic Data; Applications Center (SEDAC).

IUCN. (2016a). *IUCN red list categories and criteria (version 2016-3)*. IUCN, Gland, Switzerland.

IUCN. (2016b). Red list of threatened species (version 2016-3): Range maps. URL http://www.iucnredlist.org/ [accessed 8 December 2016]

Jambeck, J.R., Geyer, R., Wilcox, C., Siegler, T.R., Perryman, M., Andrady, A., Narayan, R. & Law, K.L. (2015). Plastic waste inputs from land into the ocean. *Science*, **347**, 768–771.

Joint Nature Conservation Committee. (2004). Common standards monitoring guidance for saltmarsh habitats.

Joppa, L.N., O’Connor, B., Visconti, P., Smith, C., Geldmann, J., Hoffmann, M., Watson, J.E.M., Butchart, S.H.M., Virah-Sawmy, M., Halpern, B.S., Ahmed, S.E., Balmford, A., Sutherland, W.J., Harfoot, M., Hilton-Taylor, C., Foden, W., Minin, E.D., Pagad, S., Genovesi, P., Hutton, J. & Burgess, N.D. (2016). Filling in biodiversity threat gaps. *Science*, **352**, 416–418.

Kaschner, K., Kesner-Reyes, K., Garilao, C., Rius-Barile, J., Rees, T. & Froese, R. (2015). *AquaMaps: Predicted range maps for aquatic species*.

Kaufmann, D., Kraay, A. & Mastruzzi, M. (2010). *The worldwide governance indicators: Methodology and analytical issues*. Social Science Research Network, Rochester, NY.

Laffoley, D. & Grimsditch, G. (Eds.). (2009). *The management of natural coastal carbon sinks*. IUCN, Gland, Switzerland.

Le Quéré, C., Raupach, M.R., Canadell, J.G., Marland et al., G., Le Quéré et al., C., Le Quéré et al., C., Raupach, M.R., Canadell, J.G., Marland, G., Bopp, L., Ciais, P., Conway, T.J., Doney, S.C., Feely, R.A., Foster, P., Friedlingstein, P., Gurney, K., Houghton, R.A., House, J.I., Huntingford, C., Levy, P.E., Lomas, M.R., Majkut, J., Metzl, N., Ometto, J.P., Peters, G.P., Prentice, I.C., Randerson, J.T., Running, S.W., Sarmiento, J.L., Schuster, U., Sitch, S., Takahashi, T., Viovy, N., Werf, G.R. van der & Woodward, F.I. (2009). Trends in the sources and sinks of carbon dioxide. *Nature Geoscience*, **2**, 831–836.

Liou, S.-M., Lo, S.-L. & Wang, S.-H. (2004). A generalized water quality index for taiwan. *Environmental Monitoring and Assessment*, **96**, 35–52.

Lowndes, J.S.S., Pacheco, E.J., Best, B.D., Scarborough, C., Longo, C., Katona, S.K. & Halpern, B.S. (2015). Best practices for assessing ocean health in multiple contexts using tailorable frameworks. *PeerJ*, **3**, e1503.

Martell, S. & Froese, R. (2013). A simple method for estimating MSY from catch and resilience. *Fish and Fisheries*, **14**, 504–514.

McGoodwin, J., R. (2001). *Understanding the cultures of fishing communities: A key to fisheries management and food security*. FAO.

Molnar, J.L., Gamboa, R.L., Revenga, C. & Spalding, M.D. (2008). Assessing the global threat of invasive species to marine biodiversity. *Frontiers in Ecology and the Environment*, **6**, 485–492.

Mora, C., Myers, R.A., Coll, M., Libralato, S., Pitcher, T.J., Sumaila, R.U., Zeller, D., Watson, R., Gaston, K.J. & Worm, B. (2009). Management effectiveness of the world’s marine fisheries (C. Roberts, Ed.). *PLoS Biology*, **7**, e1000131.

Mora, C., Tittensor, D.P., Adl, S., Simpson, A.G.B. & Worm, B. (2011). How many species are there on earth and in the ocean? (G.M. Mace, Ed.). *PLoS Biology*, **9**, e1001127.

New Zealand Ministry for the Environment. (2007). *Environment new zealand 2007.* Ministry for the Environment, Wellington, N.Z.

NOAA. (2015). Coral reef temperature anomoly database (CoRTAD version 5). URL http://www.nodc.noaa.gov/sog/cortad/

Oostendorp, R. & Freeman, R. (2012). The occupational wages around the world (OWW) database: Update for 1983-2008. URL http://www.nber.org/oww/ [accessed 4 January 2017]

Orth, R.J., Carruthers, T.J.B., Dennison, W.C., Duarte, C.M., Fourqurean, J.W., Heck, K.L., Hughes, A.R., Kendrick, G.A., Kenworthy, W.J., Olyarnik, S., Short, F.T., Waycott, M. & Williams, S.L. (2006). A global crisis for seagrass ecosystems. *BioScience*, **56**, 987.

O’Connor, S., Campbell, R., Cortez, H., Knowles, T. & others. (2009). Whale watching worldwide: Tourism numbers, expenditures and expanding economic benefits, a special report from the international fund for animal welfare. *Yarmouth MA, USA, prepared by Economists at Large*, **228**.

Pauly, D. & Zeller, D. (Eds.). (2015). Sea around us concepts, design and data.

Pitcher, T., Kalikoski, D. & Pramod, G. (2006). *Evaluations of compliance with the FAO (UN) code of conduct for responsible fisheries*. Fisheries Centre. University of British Columbia.

Porter, M. (2014). Social progress index. *Washington, DC: Social Progress Imperative*.

Ricard, D., Minto, C., Jensen, O.P. & Baum, J.K. (2012). Examining the knowledge base and status of commercially exploited marine species with the RAM legacy stock assessment database: The RAM legacy stock assessment database. *Fish and Fisheries*, **13**, 380–398.

Rosenberg, A., Fogarty, M., Cooper, A., Dickey-Collas, M., Fulton, B., Gutiérrez, N., Hyde, K., Kleisner, K., Kristiansen, Longo, C., Minte-Vera, C., Minto, C., Mosqueira, I., Osio, G., Ovando, D., Selig, E. & Thorson, J. (2014). *Developing new approaches to 116 global stock status assessment and maximum sustainable production of the seas.*

Sabine, C.L. & Tanhua, T. (2010). Estimation of anthropogenic CO \(\_{\textrm{2}}\) inventories in the ocean. *Annual Review of Marine Science*, **2**, 175–198.

Schaefer, M. (1954). Some aspects of the dynamics of populations important to the management of commercial marine fisheries. *Bulletin of the International Americal Tropical Tuna Commission*, **1**, 26–56.

Schipper, J., Chanson, J.S., Chiozza, F., Cox, N.A., Hoffmann, M., Katariya, V., Lamoreux, J., Rodrigues, A.S.L., Stuart, S.N., Temple, H.J., Baillie, J., Boitani, L., Lacher, T.E., Mittermeier, R.A., Smith, A.T., Absolon, D., Aguiar, J.M., Amori, G., Bakkour, N., Baldi, R., Berridge, R.J., Bielby, J., Black, P.A., Blanc, J.J., Brooks, T.M., Burton, J.A., Butynski, T.M., Catullo, G., Chapman, R., Cokeliss, Z., Collen, B., Conroy, J., Cooke, J.G., Fonseca, G.A.B. da, Derocher, A.E., Dublin, H.T., Duckworth, J.W., Emmons, L., Emslie, R.H., Festa-Bianchet, M., Foster, M., Foster, S., Garshelis, D.L., Gates, C., Gimenez-Dixon, M., Gonzalez, S., Gonzalez-Maya, J.F., Good, T.C., Hammerson, G., Hammond, P.S., Happold, D., Happold, M., Hare, J., Harris, R.B., Hawkins, C.E., Haywood, M., Heaney, L.R., Hedges, S., Helgen, K.M., Hilton-Taylor, C., Hussain, S.A., Ishii, N., Jefferson, T.A., Jenkins, R.K.B., Johnston, C.H., Keith, M., Kingdon, J., Knox, D.H., Kovacs, K.M., Langhammer, P., Leus, K., Lewison, R., Lichtenstein, G., Lowry, L.F., Macavoy, Z., Mace, G.M., Mallon, D.P., Masi, M., McKnight, M.W., Medellin, R.A., Medici, P., Mills, G., Moehlman, P.D., Molur, S., Mora, A., Nowell, K., Oates, J.F., Olech, W., Oliver, W.R.L., Oprea, M., Patterson, B.D., Perrin, W.F., Polidoro, B.A., Pollock, C., Powel, A., Protas, Y., Racey, P., Ragle, J., Ramani, P., Rathbun, G., Reeves, R.R., Reilly, S.B., Reynolds, J.E., Rondinini, C., Rosell-Ambal, R.G., Rulli, M., Rylands, A.B., Savini, S., Schank, C.J., Sechrest, W., Self-Sullivan, C., Shoemaker, A., Sillero-Zubiri, C., De Silva, N., Smith, D.E., Srinivasulu, C., Stephenson, P.J., Strien, N. van, Talukdar, B.K., Taylor, B.L., Timmins, R., Tirira, D.G., Tognelli, M.F., Tsytsulina, K., Veiga, L.M., Vie, J.-C., Williamson, E.A., Wyatt, S.A., Xie, Y. & Young, B.E. (2008). The status of the world’s land and marine mammals: Diversity, threat, and knowledge. *Science*, **322**, 225–230.

Schutte, V., Selig, E. & Bruno, J. (2010). Regional spatio-temporal trends in caribbean coral reef benthic communities. *Marine Ecology Progress Series*, **402**, 115–122.

Short, F.T., Polidoro, B., Livingstone, S.R., Carpenter, K.E., Bandeira, S., Bujang, J.S., Calumpong, H.P., Carruthers, T.J., Coles, R.G., Dennison, W.C., Erftemeijer, P.L., Fortes, M.D., Freeman, A.S., Jagtap, T., Kamal, A.H.M., Kendrick, G.A., Judson Kenworthy, W., La Nafie, Y.A., Nasution, I.M., Orth, R.J., Prathep, A., Sanciangco, J.C., Tussenbroek, B. van, Vergara, S.G., Waycott, M. & Zieman, J.C. (2011). Extinction risk assessment of the world’s seagrass species. *Biological Conservation*, **144**, 1961–1971.

State, U.D. of. (2016). Travel alerts and warnings. URL https://travel.state.gov/content/passports/en/alertswarnings.html [accessed 16 July 2016]

Stern, S., Wares, A. & Hellman, T. (2016). *The social progress index methodology*.

Summerson, H.C. & Peterson, C.H. (1990). Recruitment failure of the bay scallop,Argopecten irradians concentricus, during the first red tide,Ptychodiscus brevis, outbreak recorded in north carolina. *Estuaries*, **13**, 322–331.

Tallis, H.T., Ricketts, T., Guerry, A., Wood, S., Sharp, R., Nelson, E., Ennaanay, D., Wolny, S., Olwero, N., Vigerstol, K. & others. (2011). *InVEST 2.2.1 user’s guide*. The Natural Capital Project, Stanford University.

Thaminnen, J. & Arola, A. (2013). Aura OMI global surface UVB data product-OMUVBd (version 003). NASA/GSFC, greenbelt, MD, USA, NASA goddard earth sciences data and information services center (GES DISC). URL http://disc.sci.gsfc.nasa.gov/Aura/data-holdings/OMI/omuvbd\_v003.shtml [accessed 15 August 2016]

Thorbourne, R. (2011). Annopolis royal plant in canada.

Thorson, J.T., Minto, C., Minte-Vera, C.V., Kleisner, K.M., Longo, C. & Jacobson, L. (2013). A new role for effort dynamics in the theory of harvested populations and data-poor stock assessment. *Canadian Journal of Fisheries and Aquatic Sciences*, **70**, 1829–1844.

TRC. (2004). *Inventory of coastal areas of local or regional significance in the taranaki region*. Taranaki Regional Council, Stratford.

Trujillo, P. (2008). Using a mariculture sustainability index to rank countries’ performance. *Fisheries centre research reports* (eds J. Alder & D. Pauly). Fisheries Centre Research Reports, University of British Columbia, Vancouver, Canada.

UNDP. (2010). *Human development report 2010 –the real wealth of nations: Pathways to human development*. United Nations Development Programme (UNDP).

UNEP. (2015). World database on protected areas (WDPA). URL https://www.iucn.org/theme/protected-areas/our-work/world-database-protected-areas [accessed 7 June 2016]

UNEP-WCMC. (2015). World database on protected areas user manual.

UNEP-WCMC & Short, F. (2005). *Global distribution of seagrasses (version 3). third update to the data layer used in green and short (2003), superseding version 2.* UNEP World Conservation Monitoring Centre, Cambridge, UK.

United Nations. (2016a). FAO fisheries & aquaculture - fishery statistical collections - fishery commodities and trade. URL http://www.fao.org/fishery/statistics/global-commodities-production/en [accessed 29 June 2016]

United Nations. (2013a). FAO fisheries & aquaculture - fishery statistical collections - fishery commodities and trade. URL http://www.fao.org/fishery/statistics/global-commodities-production/en

United Nations. (2016b). FAO fisheries & aquaculture - fishery statistical collections - global aquaculture production. URL http://www.fao.org/fishery/statistics/global-aquaculture-production/en [accessed 29 June 2016]

United Nations. (2013b). FAO fisheries & aquaculture - fishery statistical collections - global aquaculture production. URL http://www.fao.org/fishery/statistics/global-aquaculture-production/en

United Nations. (2016c). FAO fisheries & aquaculture - fishery statistical collections - global capture production. URL http://www.fao.org/fishery/statistics/global-capture-production/en [accessed 29 June 2016]

United Nations. (2013c). FAO fisheries & aquaculture - fishery statistical collections - global capture production. URL http://www.fao.org/fishery/statistics/global-capture-production/en

United Nations. (2016d). FAO statistics division - inputs. URL http://faostat3.fao.org/browse/R/\*/E

United Nations. (2010). FAOSTAT. URL http://www.fao.org/faostat/en/#home

United Nations. (2012). Statistics division - energy statistics. URL http://unstats.un.org/unsd/energy/edbase.htm

United Nations. (2013d). Statistics division - standard country and area codes classifications (m49). URL http://unstats.un.org/unsd/methods/m49/m49regin.htm [accessed 13 April 2016]

United Nations personal communication. (2011). FAO fisheries & aquaculture - fishery statistical collections - global number of fishers. URL http://www.fao.org/fishery/statistics/global-fishers/en

Waycott, M., Duarte, C.M., Carruthers, T.J.B., Orth, R.J., Dennison, W.C., Olyarnik, S., Calladine, A., Fourqurean, J.W., Heck, K.L., Hughes, A.R., Kendrick, G.A., Kenworthy, W.J., Short, F.T. & Williams, S.L. (2009). Accelerating loss of seagrasses across the globe threatens coastal ecosystems. *Proceedings of the National Academy of Sciences*, **106**, 12377–12381.

WHO-UNICEF. (2015). Joint monitoring programme (JMP) for water supply and sanitation. URL http://www.wssinfo.org/

World Bank. (2016). GDP per capita, PPP (constant 2011 international $). URL http://data.worldbank.org/indicator/NY.GDP.PCAP.PP.KD [accessed 13 September 2016]

World Bank. (2014a). Labor force, total. URL http://data.worldbank.org/indicator/SL.TLF.TOTL.IN

World Bank. (2014b). Unemployment, total (% of total labor force) (modeled ILO estimate). URL http://data.worldbank.org/indicator/SL.UEM.TOTL.ZS

WTTC. (2013). World travel and tourism council. URL www.wttc.org/research/economicimpact-research/methodology/

WTTC. (2016). World travel and tourism council. URL www.wttc.org/research/economicimpact-research/methodology/ [accessed 14 July 2016]

Zeller, D., Palomares, M.L.D., Tavakolie, A., Ang, M., Belhabib, D., Cheung, W.W.L., Lam, V.W.Y., Sy, E., Tsui, G., Zylich, K. & Pauly, D. (2016). Still catching attention: Sea around us reconstructed global catch data, their spatial expression and public accessibility. *Marine Policy*, **70**, 145–152.
